# Formation of an invasion-permissive matrix requires TGFβ/SNAIL1-regulated alternative splicing of fibronectin

**DOI:** 10.1186/s13058-023-01736-y

**Published:** 2023-11-14

**Authors:** Héctor Franco-Valls, Elsa Tusquets-Uxó, Laura Sala, Maria Val, Raúl Peña, Alessandra Iaconcig, Álvaro Villarino, Martín Jiménez-Arriola, Pere Massó, Juan L. Trincado, Eduardo Eyras, Andrés F. Muro, Jorge Otero, Antonio García de Herreros, Josep Baulida

**Affiliations:** 1 Programa de Recerca en Càncer, Hospital del Mar Research Institute (IMIM), Dr. Aiguader, 88, 08003, Barcelona, Spain; 2 International Centre for Genetic Engineering and Biotechnology (ICGEB), Trieste, Italy; 3 Unitat Biofísica i Bioenginyeria, Facultat de Medicina i Ciències de la Salut, Universitat de Barcelona, Barcelona, Spain; 4 CIBER de Enfermedades Respiratorias, Madrid, Spain; 5 Research Program of Biomedical Informatics, Hospital del Mar Research Institute (IMIM), Barcelona, Spain; 6 John Curtin School of Medical Research, Australian National University, Canberra, Australia; 7 Departament de Medicina i Ciències de la Vida, Universitat Pompeu Fabra, Barcelona, Spain; 8 Present Address: Institute for Research in Biomedicine, Barcelona, Spain; 9 Present Address: National Institutes of Health: Intramural Research Program, Bethesda, MD USA; 10 Present Address: Vall Hebron Institute of Research, Barcelona, Spain; 11 Present Address: Josep Carreras Leukaemia Research Institute, Barcelona, Spain

**Keywords:** Extracellular matrix, Matrix architecture, TGFβ, SNAIL1, EDA+ Fibronectin, Metastasis, Matrix rigidity, Breast cancer, Myofibroblasts

## Abstract

**Background:**

As in most solid cancers, the emergence of cells with oncogenic mutations in the mammary epithelium alters the tissue homeostasis. Some soluble factors, such as TGFβ, potently modify the behavior of healthy stromal cells. A subpopulation of cancer-associated fibroblasts expressing a TGFβ target, the SNAIL1 transcription factor, display myofibroblastic abilities that rearrange the stromal architecture. Breast tumors with the presence of SNAIL1 in the stromal compartment, and with aligned extracellular fiber, are associated with poor survival prognoses.

**Methods:**

We used deep RNA sequencing and biochemical techniques to study alternative splicing and human tumor databases to test for associations (correlation t-test) between SNAIL1 and fibronectin isoforms. Three-dimensional extracellular matrices generated from fibroblasts were used to study the mechanical properties and actions of the extracellular matrices on tumor cell and fibroblast behaviors. A metastatic mouse model of breast cancer was used to test the action of fibronectin isoforms on lung metastasis.

**Results:**

In silico studies showed that SNAIL1 correlates with the expression of the extra domain A (EDA)-containing (EDA+) fibronectin in advanced human breast cancer and other types of epithelial cancers. In TGFβ-activated fibroblasts, alternative splicing of fibronectin as well as of 500 other genes was modified by eliminating SNAIL1. Biochemical analyses demonstrated that SNAIL1 favors the inclusion of the EDA exon by modulating the activity of the SRSF1 splicing factor. Similar to *Snai1* knockout fibroblasts, EDA- fibronectin fibroblasts produce an extracellular matrix  that does not sustain TGFβ-induced fiber organization, rigidity, fibroblast activation, or tumor cell invasion. The presence of EDA+ fibronectin changes the action of metalloproteinases on fibronectin fibers. Critically, in an mouse orthotopic breast cancer model, the absence of the fibronectin EDA domain completely prevents lung metastasis.

**Conclusions:**

Our results support the requirement of EDA+ fibronectin in the generation of a metastasis permissive stromal architecture in breast cancers and its molecular control by SNAIL1. From a pharmacological point of view, specifically blocking EDA+ fibronectin deposition could be included in studies to reduce the formation of a pro-metastatic environment.

**Supplementary Information:**

The online version contains supplementary material available at 10.1186/s13058-023-01736-y.

## Background

Epithelial tumor formation and progression toward malignant stages are sustained by the acquisition of aberrant and extemporary cell behaviors. An initial exacerbated cell proliferation induced by oncogenic mutations is followed by reactivation of inappropriate cell plasticity programs, providing tumor cells with motility, invasiveness, regenerative potential, immune evasion and resistance to anoikis and pharmacological insults [1, 2]. These programs are largely influenced by biochemical and biophysical signaling from the tumor-destabilized microenvironment; therefore, certain cancer stroma conditions can cause malignant events, such as tumor invasion, relapse and therapeutic resistance.

Of the tumor microenvironment cells, fibroblasts are primarily responsible for regulating the architectural features. While stroma structure generated by normal fibroblasts restricts cell movements and maintains epithelial tissue homeostasis, structures generated by the excessive activity of cancer-associated fibroblasts (CAFs) on the extracellular architecture favor plasticity programs fueling tumor progression [3]. For instance, the presence of directionally aligned collagen around the tumor mass was found to be a signature for poor prognostics in human breast carcinoma [4], and evidence of central fibrosis in triple-negative breast cancer correlates with the highest propensity for developing distant metastases [5].

CAF are heterogenous, and while myofibroblastic CAF (myoCAF) strongly remodel the extracellular matrix (ECM), immunostimulatory CAFs (iCAFs) modulate the immune system. In triple-negative breast tumors, the presence of CAFs enriched for high expression of immune-related genes, but not for that of ECM-regulating genes, is associated with longer overall survival [6]. Therefore, characterizing the molecular mechanisms sustaining the pro-metastatic myoCAFs presents a major challenge to design effective antitumor strategies. Strategies targeting no particular CAFs in pancreatic tumors do not have sufficient therapeutic efficacy or, in some contexts, even lead to shortened patient survival in clinical trials [7].

The transforming growth factor beta (TGFβ) is a secreted cytokine that promotes a fibroblast-to-myofibroblast transition [8]. TGFβ-activated fibroblasts, including mouse embryonic fibroblasts (MEF), secrete a myofibroblast-specific ECM with anisotropic fibers and elevated rigidity [9]. The TGFβ action on the ECM properties and the subsequent malignant effects on tumor cells (such as increased directional migration and invasiveness) are dependent on the transcription factor SNAIL1 [10]. SNAIL1 levels in CAF lines correlate with the extracellular anisotropy they produce, and SNAIL1 protein expression in colon and breast cancers is observed mostly in the stromal compartment associated with aligned fibronectin fibers and poor prognosis [9–12].

Cellular fibronectin is a glycoprotein assembled mainly by fibroblasts into extracellular fibers, where it acts as a template for the directional polymerization of other ECM fibers [13]. The extracellular fibronectin fibrillogenesis is a cell-mediated assembly process in which dimers of fibronectin secreted or recruited to the external cell membrane receive tension from receptors coupled to intracellular stress fibers [14]. Fibronectin is encoded by a single gene (*FN1*) that generates multiple isoforms by alternative splicing. Extra domain A isoforms (EDA+) result from the inclusion of exon 33 [15]. Heterodimers can form from different fibronectin isoforms, and all isoforms can be incorporated into the ECM fibers [16]. Myofibroblasts in embryogenesis, wound healing and fibrosis [17–19], but not fibroblasts in adult tissues, express EDA+ fibronectin variants [20]. Additionally, EDA+ fibronectin has also been detected in many cancers [21–27].

Most seminal studies on the role of EDA in activating epithelia [28–30], fibroblasts [31, 32] and inflammatory cells [33], and well as on its interaction with other proteins [33], have used recombinant, non-polymerized full-length isoforms or fibronectin fragments. Thus, despite our knowledge about its cell signaling actions, we still lack a full understanding of the structural functions of polymerized EDA+ fibronectin in the ECM. TGFβ promotes EDA inclusion in part through a mechanism that facilitates mobilization of the splicing factor SRSF1, which activates the splicing machinery [34, 35]. The direct involvement of SNAIL1 in alternative splicing regulation has not been described.

In this article, we unveil a TGFβ/SNAIL1-dependent molecular mechanism that controls EDA inclusion by SRSF1. We show a correlation between the expression of SNAIL1 and EDA+ fibronectin in advanced breast tumors. Further, we show that EDA+ fibronectin mimics the action of SNAIL1 in fibroblasts by fine-tuning the architectural parameters of the ECM and influencing the tumor cell invasiveness. This contribution toward understanding the molecular mechanisms underlying myofibroblast activity is a step forward in the development of strategies aimed at blocking CAF subtypes with pro-metastatic activity.

## Materials and methods

### Reagents, cell lines and patient-derived xenografts

Cells were grown in standard medium and conditions. MDA-MB-231, MCF7, HT-29 M6 and NIH3T3 cells were acquired from the repository stock at the Institut Hospital del Mar d’Investigacions Mèdiques (IMIM). Control and *Snai1* KO mouse embryonic fibroblasts (MEF), mesenchymal stem cells (MSC) [36] and control and *Snai1* KO breast cancer-associated fibroblasts (CAF) [43] were previously established in our laboratory. MSC are isolated non-transformed fibroblasts that are recruited by tumors in vivo. The MEF lines expressing EDA- or EDA+ fibronectin or control unmodified MEF were prepared at the ICGEB (Trieste, Italy). BJ human fibroblasts were kindly gifted by Dr. Cristina Peña (Hospital Universitario Puerta de Hierro). The EpRas tumor cell line was kindly provided by Dr. Antoni Celià lab (IMIM) and were originally generated by Dr. Robert Weinberg (Whitehead Institute for Biomedical Research). The AT-3 tumor cell line was kindly gifted by the Dr. José Yelamos Lab (IMIM). Patient-derived xenografts (PDX) were provided by Dr. Joaquín Arribas (IMIM) and previously analyzed in our laboratory [37]. See Additional file 1 Materials and Methods for treatment reagents and transfected siRNAs.

### Human tumor database information

Fibronectin RNA expression and splicing data were obtained from TSVdb [38], and the SNAIL1 protein levels for the same cohort of patients, from cBioPortal. The characteristics of each patient were also downloaded from the cBioPortal database. Thus, all procedures performed in studies involving human participants were in accordance with the ethical standards of the institutional and/or national research committee and with the 1964 Helsinki Declaration and its later amendments or comparable ethical standards.

**Fig. 1:**
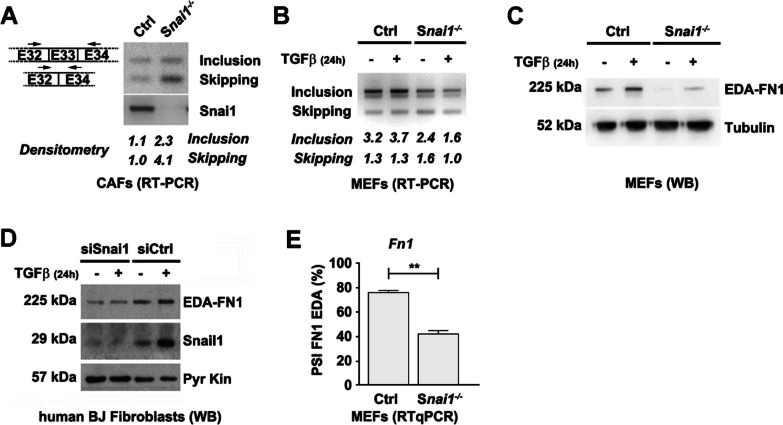
SNAIL1 expression controls fibronectin EDA inclusion. **A** Relative RNA amount of EDA+ fibronectin isoforms in control and *Snai1* KO CAFs. RNA obtained from indicated CAFs was retrotranscribed and amplified by PCR using *Fn1* (depicted on the left) or *Snai1* primers. Resulting DNA was visualized by electrophoresis on a 2% agarose gel. A representative experiment from the three performed is shown. **B** Relative RNA amount of EDA+ fibronectin isoforms in control and *Snai1* KO MEFs treated with TGFβ_1_. RNA from the indicated MEFs was untreated or treated for 24 h with 5 ng/mL of TGFβ_1_ and then analyzed as in **A**. **C** Expression of the EDA+ fibronectin protein in MEFs. Control and *Snai1* KO MEFs were treated or not with 5 ng/mL of TGFβ_1_ for 24 h and lysed in SDS buffer. Levels of the indicated proteins were analyzed by Western blotting. **D** Expression of EDA+ fibronectin in BJ fibroblasts. Human BJ fibroblasts were transfected with siRNA anti-SNAIL1 or a control siRNA and then treated or not with TGFβ_1_ for 24 h. Cells were lysed in SDS buffer, and protein levels were analyzed by Western blotting. **E** Quantification of the EDA+ fibronectin ratio by RNA-seq. Deep sequencing of RNA from control or *Snai1* KO MEFs treated with 5 ng/mL TGFβ_1_ for 3 h was performed. The percent spliced-in (PSI) for FN1-EDA in each condition was calculated from the number of inclusion and exclusion sequencing reads

For each cancer type studied (lung adenocarcinomas, skin melanomas, breast adenocarcinomas and kidney renal cell carcinomas), tumor data were analyzed in two groups: i) stages I and II, and ii) stages III and IV. The number of patients per group ranged from 69 to 590 (Fig. 1). Each tumor was classified according to its relative SNAIL1 protein levels and the percentage of EDA inclusion. Only full-length fibronectin isoforms, including or excluding exon 33, were considered. The cutoff value to discriminate between low and high expression was set in relation with the average value for the EDA percentage in each cancer type, ranging between 68 and 96%; the value was set at 80% for kidney cancer, 85% for breast cancer, 90% for lung cancer, and 99% for skin cancer. Normal tissue data were analyzed with these values, and only 3–8% of specimens expressed high EDA (Fig. 1). Normal tissue data for skin cutaneous melanoma were not available. As SNAIL1 protein stability is tightly regulated post-translationally [39], protein but not RNA levels were analyzed. A cutoff value of 1 was set by SNAIL1 expression data from Reverse-Phase Protein Array, normalized by z-score. For lung cancer, no normalized data were available, and the cutoff was set at 0.25 as the average value of the collection was –0.22. With these settings, advanced tumors (stages III and IV) concomitantly expressing high levels of SNAIL1 and fibronectin EDA represented 11.1%, 2.9%, 2.7% and 2.1% of the lung, skin, breast and kidney cancers, respectively. For PDXs, the SNAIL1 and EDA+ fibronectin cutoff values corresponded to 80% of the band intensity obtained in a positive control sample from activated EDA+ MEFs. Seven of 29 PDXs (24%) expressed high levels of SNAIL1 and EDA+ fibronectin. For statistical analysis, percentages of elevated EDA inclusion in high versus low SNAIL1 tumors were compared.

### RNA immunoprecipitation

Cells on cell culture dishes were grown to 80% confluence, washed with warm PBS, trypsinized and recovered by centrifugation. Cells were then resuspended in 300 µL lysis buffer (100 mM KCl, 5 mM MgCl_2_, 10 mM HEPES pH 7, 0.5% NP-40, 1 mM DTT, 1 × RNase inhibitor, 1 × protease inhibitor) for each 15-cm-diameter plate, incubated 5 min on ice and snap-frozen in liquid nitrogen. Thawed samples were sonicated in a Bioruptor Pico Sonicator (Diagenode) for 15 cycles of 30 s ON/OFF at 4 °C. Samples were centrifuged at 16,000 g for 10 min at 4 °C, the supernatants were recovered and the protein content was quantified. In parallel, antibody-complexed beads were prepared. Per sample, 30 μL of Gammabind G Sepharose beads was washed in NET buffer (50 mM Tris pH 7.5, 150 mM NaCl, 0.1% NP-40, 1 mM EDTA); samples were blocked with 20 μg tRNA, washed again and incubated for 2 h at 4 °C with rotation after adding the primary antibody or Irrelevant IgG. All centrifugation steps were done at 350 g and 4 °C. Protein samples were precleared with unblocked beads for 30 min at 4 °C with rotation and centrifuged for 2 min at 350 g, and the supernatant was recovered. A total of 6 mg of protein was mixed with the previously blocked and antibody-complexed beads and incubated for 2 h at 4 °C with rotation. The mix was centrifuged, and beads were washed 4 times with NET buffer. Finally, beads were resuspended in 50 µL NET buffer with 2 µL of blue glycogen and 150 µL TRIzol, and RNA extraction was carried out as described.

### Measurement of the micromechanical properties by atomic force microscopy

Micromechanics of the matrices were measured using a custom-built atomic force microscope mounted on an inverted optical microscope (TE2000; Nikon, Tokyo, Japan). All experiments were performed in PBS buffer with a pH of 7.4 at 37 °C. Measurements were performed by using force–displacement curves on the surface of the sample with V-shaped silicon nitride microfabricated cantilevers (0.012 N/m of nominal spring constant) ended with a 2.5 μm radius spherical glass bead (Novascan Technologies, Ames, IA, US). The vertical position of the cantilever was controlled with a piezoelectric actuator and measured with a strain gauge sensor (Physik Instrumente, Karlsruhe, Germany). A four-quadrant photodiode (S4349, Hamamatsu, Japan) was used to measure the deflection of the cantilever. The elastic modulus was calculated from the force–displacement curves by fitting them to the Hertz model for sphere-plane contact, as described in [40], and computed at an indentation of 0.5 μm. Samples were probed in five randomly selected zones, with five different points probed in each zone (separated by at least 10 µm in the *XY* plane), for a total of twenty-five elastic modulus measurements in each sample.

### Statistical analysis

All results shown are representative of at least three independent experiments. Data are represented as the mean ± SEM. When appropriate, statistical analyses were conducted using GraphPad Prism software (GraphPad, La Jolla, CA, USA), and data were analyzed for significance using unpaired t-tests and chi-squared tests. Values of *p* < 0.05 are marked with one asterisk and of *p* < 0.01 with two asterisks.

See Additional file 1 Materials and Methods for standard protocols and specific reagents for RNA extraction, reverse transcription and PCR, RNA-seq and alternative splicing analysis, Western blotting, immunofluorescence analysis, immunohistochemistry, collagen imaging, immunoprecipitation assay, chromatin immunoprecipitation, fibroblast activity, deposition of three-dimensional extracellular matrices, invasion assays and statistical analysis. Uncropped gel and blot images are provided with the Additional file 1.

All animal procedures were approved by the Animal Research Ethical Committee from the Parc de Recerca Biomèdica de Barcelona (Barcelona, Spain) and by the Generalitat de Catalunya.

## Results

### Depletion of fibroblastic *Snai1* decreases the EDA exon inclusion

Expression of both the transcription factor SNAIL1 and the fibronectin isoform including the EDA domain in myofibroblasts has been described [20, 39]. To determine whether SNAIL1 is involved in the controlling EDA exon inclusion, we first used RT-PCR to visualize the relative amount of skipping/inclusion isoforms in RNA from control or *Snai1*-deficient fibroblasts, including CAFs, MEFs and human BJ fibroblasts. Oligonucleotide primers annealing to the flanking EDA exons were used at not saturating cycles. We observed a qualitative switch toward EDA-skipped RNA, confirmed by densitometric quantification of the bands, in *Snai1* KO relative to control CAFs (Fig. 1A). As described, we detected that the cytokine TGFβ promoted EDA+ fibronectin RNA expression and that the increase was SNAIL1 dependent (Fig. 1B; Additional file 1: Fig. S1). By analyzing EDA+ fibronectin expression at the protein level using a specific monoclonal antibody, we found that it was only faintly detected in *Snai1* KO fibroblasts as compared to control cells (Fig. 1C). In human BJ fibroblasts, depletion of SNAIL1 using a specific siRNA downregulated EDA+ fibronectin levels in both untreated and TGFβ-treated cells (Fig. 1D) indicates that the *Snai1-*depletion effect was not limited to murine MEFs and CAFs or to the constitutive removal of the factor.

To elucidate whether SNAIL1 extensively controls alternative splicing, we evaluated splicing events by deep sequencing of mRNA from control and *Snai1* KO MEFs treated for 3 h with TGFβ. The SUPPA2 and SANJUAN pipelines revealed 674 and 299 significantly different splicing events, respectively, that affected more than 500 genes (Additional file 1: Fig. S2A, Tables S1 and S2). Among these, we found several genes involved in actin cytoskeleton regulation, such as *Myl6*, *AnIn*, *Macf1*, *Tpm1*, *Tpm2*, *PP1R12A*, *FlnC* and *FlnB*, as well as ECM genes, such as *Col5α1* and *FN1*; we confirmed some of these genes by RT-PCR (Additional file 1: Fig. S2B). For fibronectin, the number of RNA-seq reads for the events including and excluding EDA allowed us to quantify the relative amount of EDA+ fibronectin in each sample, which was over 75% in control MEFs but was reduced by approximately half in *Snai1* KO cells (Fig. 1E).

### SNAIL1 protein levels correlate with EDA+ fibronectin RNA expression in advanced cancers

**Fig. 2:**
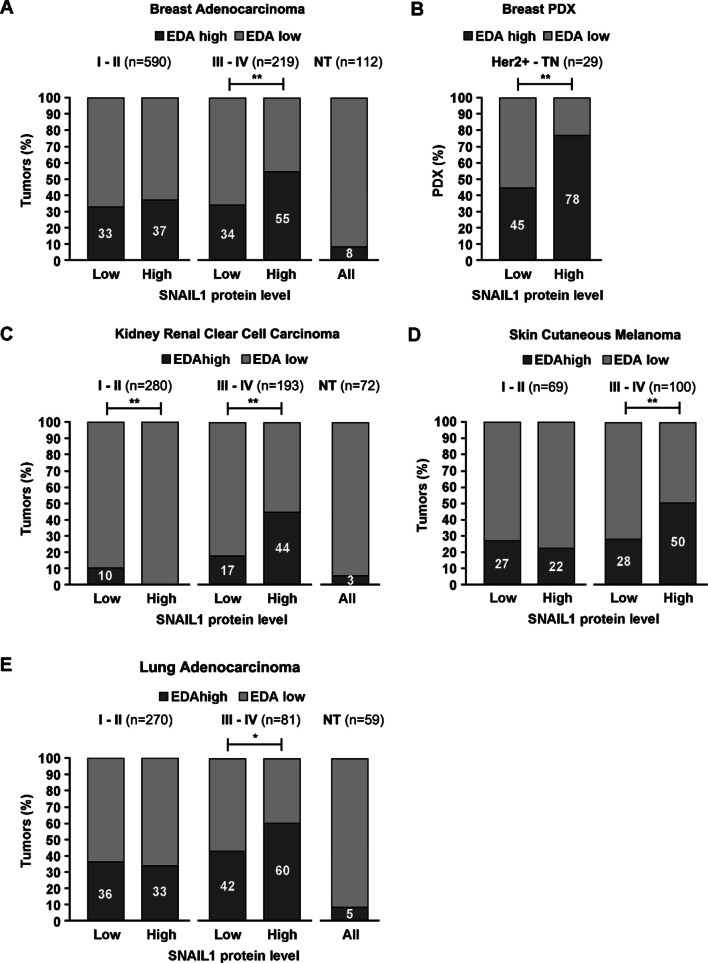
Elevated percentages of EDA+ fibronectin correlate with high SNAIL1 levels in advanced human tumors.** A** Breast adenocarcinoma; **C** kidney renal clear cell carcinoma; **D** skin cutaneous melanoma; and **E** lung adenocarcinoma. The percentage of EDA+ fibronectin RNA and the SNAIL1 protein level in each specimen were obtained from the TSVdb and cBioPortal databases, respectively. Tumors were classified according to the levels (low or high) of SNAIL1 and EDA+ fibronectin (see Materials and Methods), and the percentages of high or low EDA+ fibronectin tumors were plotted for each SNAIL1-expressing category. Tumors at the initial (I and II) or advanced (III and IV) stages were analyzed separately (see Materials and Methods). Numbers within the bars indicate the percentage of tumors with high levels of EDA+ fibronectin. When available, data for normal tissue (NT) are also shown. n, number of tumors per group. **B** Relative protein levels of EDA+ fibronectin and SNAIL1 in PDXs. EDA+ fibronectin and SNAIL1 levels in 29 PDX protein extracts from HER2+ or triple-negative breast neoplasms were densitometrically estimated from Western blots (see Materials and Methods). PDXs were classified according to their levels (low or high) of SNAIL1 and EDA+ fibronectin (see Materials and Methods) and analyzed as in A. n, number of PDXs

In primary breast tumors, the presence of myofibroblatic CAFs has been associated with tumor progression [5, 6, 10, 41], and the expression of fibroblastic SNAIL1, with a poor prognosis [10]. Here, we analyzed whether the simultaneous expression of SNAIL1 and EDA+ fibronectin correlates with advanced breast tumors. We collected available SNAIL1 protein data (cBioportal; TCGA, Firehose Legacy) and calculated the percentage of EDA + fibronectin RNA (using the web tool TSVdb for the TCGA splicing variants) from 809 invasive breast carcinomas. We categorized tumors according to their stage (I-II or III-IV) and calculated the relative levels for each molecule using a cutoff value (Material and Methods). Overall, 55% of advanced tumors (stages III and IV) expressing high levels of SNAIL1 also expressed high levels of EDA+ FN1. This percentage decreased to 33–37% in the remaining categories (Fig. 2A). In protein extracts from patient-derived xenografts (PDX) corresponding to HER2 + or triple-negative breast neoplasms, the percentage of tumors with high EDA+ fibronectin expression was increased in the PDXs expressing high levels of SNAIL1 (Fig. 2B). Remarkably, we found similar associations when we analyzed available data from other solid tumors in which the stromal component has been shown to be relevant for tumor progression, such as skin cutaneous melanoma [42], lung adenocarcinoma [43, 44] and kidney renal clear cell carcinoma [45] (Fig. 2B–D). Even though available data do not discriminate between tumor and stroma expression, our analysis shows that the expression of two myofibroblast-associated proteins mostly present in the stroma of colon and breast tumors was associated with advanced tumors.

### SRSF1 binding to exon 33 RNA of fibronectin is SNAIL1-dependent

**Fig. 3:**
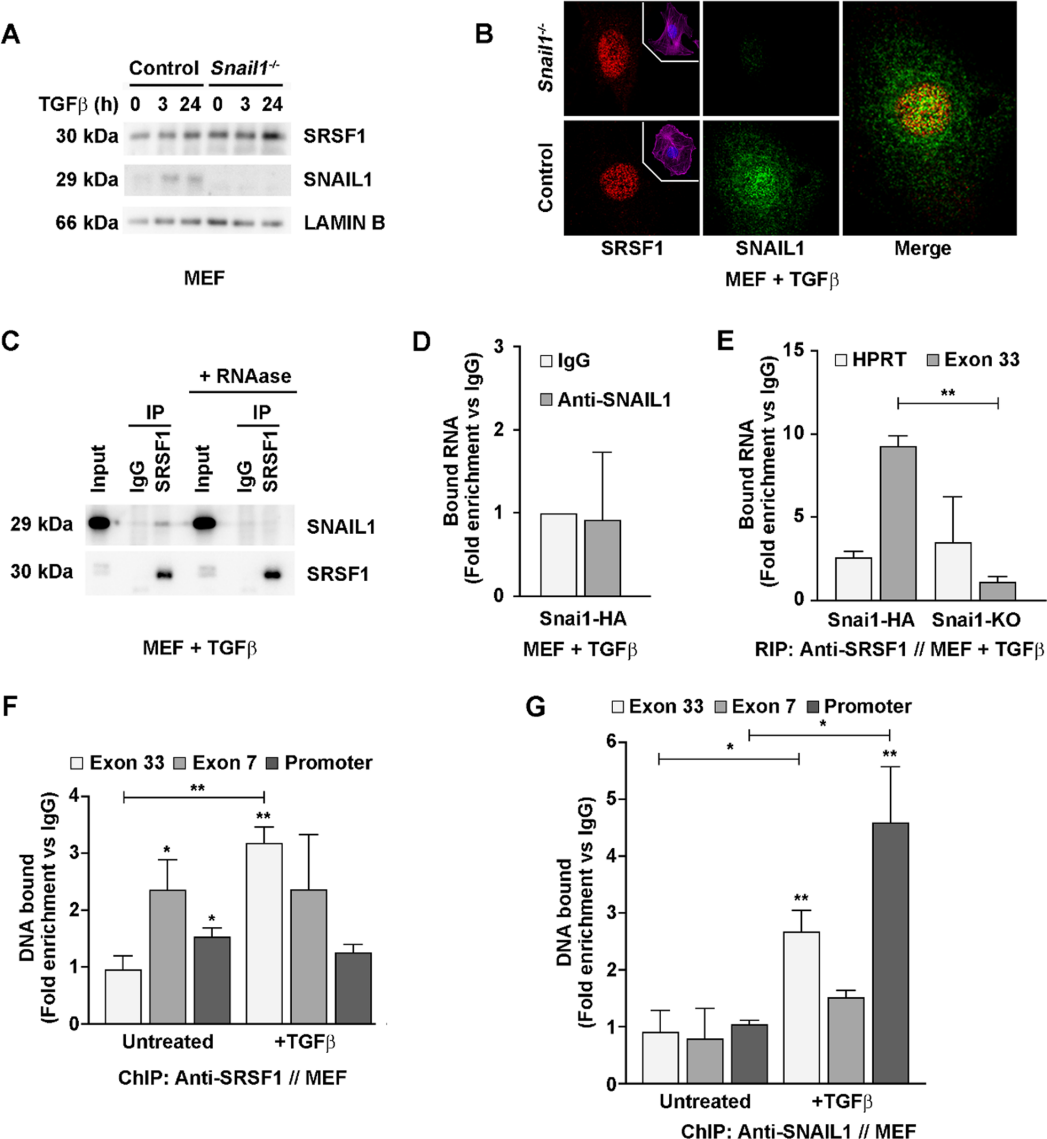
SRSF1 interactions with the fibronectin EDA exon is regulated by SNAIL1. **A** SRSF1 protein amount in *Snai1* KO MEFs. Control and *Snai1* KO MEFs were lysed in SDS buffer after the indicated times of TGFβ_1_ treatment (5 ng/mL), and protein levels were analyzed by Western blotting. **B** SNAIL1 and SRSF1 colocalize in the nucleus of MEFs. Control and *Snai1* KO MEFs were grown on glass coverslips, treated with TGFβ_1_ for 24 h and fixed with 4% PFA. The cellular distributions of SNAIL1 and SRSF1 were analyzed by immunofluorescence with specific antibodies. Images were obtained by confocal microscopy. Phalloidin (pink) and DAPI (blue) staining corresponding to depicted cells are shown into a box. Merge images in control MEFs were produced with ImageJ, and colocalization is shown in yellow. **C** The SNAIL1 and SRSF1 interaction is RNA dependent. Extracts of MEFs treated with TGFβ_1_ for 3 h were obtained in RIPA buffer, and half of the sample was treated with 400 µg/mL RNase A. RT-qPCR for total fibronectin confirmed the complete elimination of RNA in the samples. Immunoprecipitation was performed using an antibody specific for SRSF1 and agarose beads. Immunoprecipitated proteins were analyzed by Western blotting. **D** SNAIL1 does not bind to the fibronectin exon 33 RNA. RNA immunoprecipitation (RIP) was performed using an antibody specific for SNAIL1 or an unspecific IgG in samples of MEFs transfected to overexpress *Snai1-HA* (Additional file 1: Fig. S3) and treated with 5 ng/mL TGFβ_1_ for 3 h. RNA enrichment in the immunoprecipitates was analyzed by RT-qPCR using primers for exon 33. Bars show binding enrichment as compared to immunoprecipitation using IgG. **E** SRSF1 binds to the fibronectin exon 33 RNA in a SNAIL1-dependent manner. RIP was performed using an antibody specific for SRSF1 in samples of MEFs transfected to overexpress *Snai1-HA* (Additional file 1: Fig. S3), or of MEFs KO for *Snai1* treated with 5 ng/mL TGFβ_1_ for 3 h. RNA enrichment in the immunoprecipitates was analyzed by RT-qPCR using primers for exon 33 or HPRT (as a control). Bars show binding enrichment compared to immunoprecipitation using unspecific control IgG. At least three replicates were performed for each immunoprecipitation. **F** and **G** SRSF1 and SNAIL1 bind to the EDA coding region in a TGFβ-dependent manner. ChIP was performed with an antibody specific for SRSF1 (**F**) or SNAIL1 (**G**) in samples of MEFs transfected to overexpress SNAIL-HA that were treated or not with TGFβ_1_ for 3 h. Precipitated DNA was analyzed by qPCR using primers targeting *Fn1* promoter (+ 116/ + 265), *Fn1* exon 7 and *Fn1* exon 33 (EDA). Bars show binding enrichment as compared to immunoprecipitation using unspecific IgG. At least three replicates were performed for each immunoprecipitation

To further analyze how SNAIL1 controls the EDA inclusion, we focused on SRSF1, a splicing factor involved in controlling this event [34, 35]. Although SRSF1-mediated splicing can be regulated by factor availability [34], we did not detect decreased SRSF1 protein levels or changes in its subcellular localization in TGFβ-activated *Snai1* KO relative to control MEFs (Fig. 3A,B). In contrast, we observed that SRSF1 and SNAIL1 colocalized in nuclear granules (Fig. 3B) and co-immunoprecipitated (Fig. 3C), suggesting a more direct molecular connection.

As SRSF1 was described to bind EDA RNA [46], we tested whether SNAIL1 influences SRSF1 binding to exon 33 RNA using RNA immunoprecipitation (RIP) assays in *Snai1* KO MEFs. SRSF1 or SNAIL1 was immunoprecipitated with specific antibodies, and the co-precipitating RNA was analyzed. Fibronectin RNA did not significantly co-precipitate with anti-SNAIL1, even in MEFs overexpressing ectopic SNAIL1-HA treated with TGFβ (Fig. 3D). RIP using anti-SRSF1 in these cells confirmed that SRSF1 interacts with EDA RNA, as previously reported (Fig. 3E). Remarkably, the binding of SRSF1 to the EDA RNA region (but not to a control irrelevant region, HPRT) was undetected in *Snai1* KO MEFs (Fig. 3E). This result indicates that SNAIL1 is required for the specific binding of SRSF1 to the exon 33 RNA.

Splicing factors of the SRSF family are involved in coupling RNA Pol II transcription to pre-mRNA splicing [47] and precipitate genomic DNA in the presence of crosslinking agents [48]. Therefore, we used ChIP to test whether SRSF1 interacts with the genomic DNA at exon 33. We detected that TGFβ promoted precipitation of this region, but not of the fibronectin proximal promoter or exon 7 regions, with SRSF1 (Fig. 3F), indicating that the splicing factor is likely involved in a co-transcriptional complex. As SNAIL1 interacts with SRSF1, we tested whether SNAIL1 also interacts with the exon 33 genomic region. In contrast to the lack of its binding to RNA, we found that SNAIL1 interacted with the exon 33 in a TGFβ-dependent manner, as well as with the proximal FN1 promoter (as previously described; [49]) (Fig. 3G). Lack of binding to the fibronectin exon 7 region supported the specificity of these ChIP interactions.

Altogether, our data suggest that TGFβ induces localization of SNAIL1 at the alternative splicing region, which is required for the formation of a co-transcriptional complex that includes SRSF1 bound to the EDA RNA. In this case, we would expect that the DNA and RNA binding machineries (including SNAIL1 and SRSF1, respectively) connected by nascent RNA, would be separated by an RNase treatment. Indeed, co-immunoprecipitation of SNAIL1 and SRSF1 was disrupted when input lysates were treated with RNase (Fig. 3C).

As the reduction of the EDA-containing isoform in untreated *Snai1* KO MEFs (Fig. 1A–C) could be independent of SRSF1, we tested the levels of other splicing factors involved in the regulation of EDA splicing, such as SFSR3, SRSF5 [20, 50] and QKI [51]. While the levels of the splicing silencer QKI increased in the KO MEFs (Additional file 1: Fig. S4A), no clear changes were observed for SRSF1 (Fig. 3A), SRSF3 or SRSF5 (Additional file 1: Fig. S4B). In contrast, in epithelial cells that did not express endogenous SNAIL1 or EDA+ fibronectin, exogenous expression of SNAIL1-HA promoted the increase in EDA+ fibronectin, as well as of SRSF5 and (to a lesser extent) SRSF3 (Additional file 1: Fig. S5A and B).

### Fibronectin EDA sustains structural ECM properties regulated by SNAIL1

**Fig. 4:**
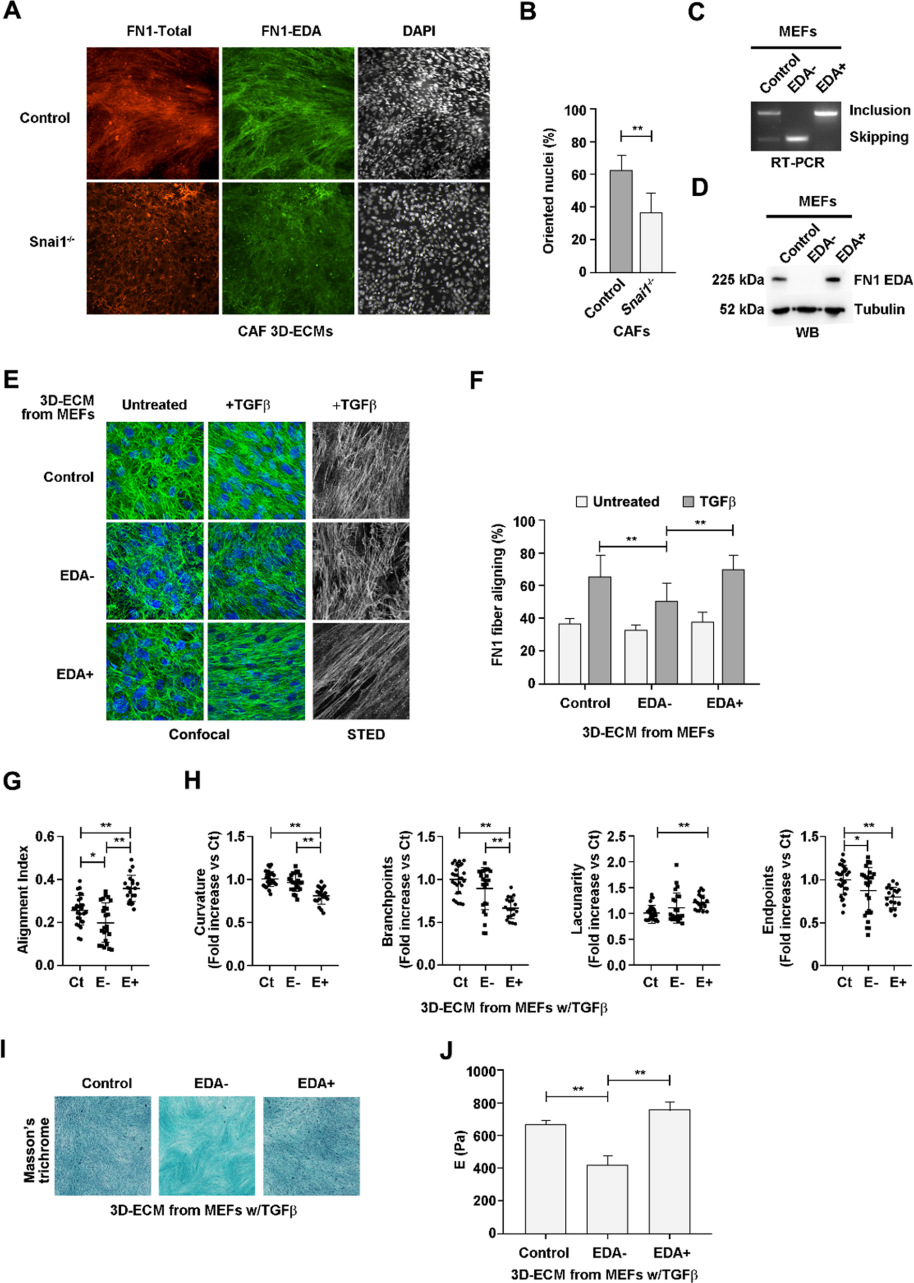
Fibronectin EDA determines topological and mechanical properties of myofibroblastic matrices. **A** CAF-derived 3D ECMs. Indicated CAFs were seeded on coverslips and allowed to produce ECM for 6 days. Cell cultures were then fixed and analyzed by immunofluorescence (IF) with an anti-fibronectin (red), anti-fibronectin EDA (green) and DAPI (white). **B** Quantification of the oriented CAF nuclei within 3D ECMs. Orientation angles of the DAPI-stained nuclei were calculated using the ImageJ analysis particles tool. Percentage of nuclei orientated toward the most frequent angle (up to 21° deviation) is shown. **C** Relative RNA amount of EDA+ fibronectin isoforms in genetically modified MEF lines. RNA from control, EDA– and EDA+ MEFs treated with 5 ng/mL of TGFβ_1_ for 3 h was retrotranscribed and amplified using primers flanking exon 33 of *Fn1* (as described in Fig. 1) and visualized by DNA-electrophoresis. **D** Relative protein amount of EDA+ fibronectin isoforms in genetically modified MEF lines. Indicated MEFs treated as in **C** were lysed in SDS buffer, and the levels of the indicated proteins were analyzed by Western blot. **E** Fibronectin fibers in 3D ECMs. Indicated MEFs seeded on coverslips were allowed to produce extracellular matrix for 6 days in the presence or absence of 5 ng/mL TGFβ_1_. Cell cultures were then fixed and analyzed by IF with an anti-fibronectin (green) and DAPI. Confocal and STED microscopy were used to obtain images. **F** Quantification of fibronectin fiber alignment in 3D ECMs. The fiber angles were calculated using the ImageJ plugin OrientationJ. The percentage of fibers aligned toward the same direction (up to 21° deviation from the mode) is shown. **G** Quantification of fibronectin fiber alignment index through TWOMBLI. Fibronectin fiber images obtained as in **E** were analyzed using the ImageJ macro TWOMBLI. All obtained data are plotted, showing all individual measurements, mean and SEM. **H** Quantification of fibronectin fiber parameters through TWOMBLI. The indicated parameters were analyzed from images used in **E**. Arbitrary units provided by the plugin are expressed relative to wild-type MEFs. **I** Visualization of collagen deposition from in vivo–like extracellular matrices**.** 3D ECMs were produced as in **C** fixed with 4% PFA and stained with Masson's trichrome. **J**, Quantification of the stiffness of in vivo–like extracellular matrices. 3D ECMs generated as in **E** were decellularized, and the elastic modulus was calculated from atomic force-curve measurements

Our previous research has shown that the extracellular anisotropy generated by CAF lines correlates with their SNAIL1 levels and that TGFβ-treated fibroblasts produce anisotropic matrices in a SNAIL1-dependent manner [10]. Analogously, we found here that our CAF line but not the *Snai1* KO one generates anisotropic three-dimensional extracellular matrices (3D ECMs). In detail, IF analysis showed aligned fibronectin fibers that decrease in *Snai1* KO ECMs (Fig. 4A) and angle measurement of DAPI-stained nuclei indicated that the high proportion of oriented nuclei were decreased by half (Fig. 4B).

Moreover, we observed a reduction of the presence of EDA+ linear fibronectin fibers in ECMs from KO CAFs (Fig. 4A). Therefore, we analyzed the contribution of the EDA+ fibronectin on the extracellular topology using TGFβ-activated MEFs derived from two genetically engineered mouse strains that exclusively expressed either EDA-including (EDA+) or -excluding (EDA–) fibronectin isoforms [19]. RNA analysis confirmed the expression of the corresponding isoforms in these cell lines (Fig. 4C). In control MEFs under standard cell culture conditions, the expression of EDA+ fibronectin was predominant (Figs. 1A,B,E and 4C). Proteins corresponding to EDA+ fibronectin were detected only in the control and EDA+ MEFs (Fig. 4D). 3D ECMs from these MEF lines were produced and analyzed by immunofluorescence with an anti-fibronectin antibody recognizing both EDA+ and EDA– isoforms. The absence of EDA+ fibronectin in EDA– 3D ECMs was confirmed by immunofluorescence (Additional file 1: Fig. S6). In the absence of TGFβ, all lines produced randomly oriented fibronectin fibers (Fig. 4E). Fiber orientation was estimated to be over 33% with the OrientationJ plugin (Fig. 4F), as reported for unaligned fibers deposited by control fibroblasts [9, 10]. In the presence of TGFβ, the increase of oriented fibronectin fibers was significantly lower in matrices produced by EDA– MEFs (Fig. 4E,F), indicating that the presence of the EDA within the matrix favors TGFβ-induced aligned polymerization of fibronectin fibers. High-resolution images obtained by STED (stimulated emission depletion) microscopy illustrated the differences in the fiber nets between the three matrices (Fig. 4E).

A higher fiber alignment in EDA+ relative to EDA– matrices (Fig. 4G), as well as a lower curvature and branch points (Fig. 4H), was confirmed with the TWOMBLI plugin for FIJI [52]. Additionally, differences between the control and EDA+ matrices affecting alignment, curvature, branch points, lacunarity and endpoints were also detected (Fig. 4H). As extracellular fibronectin acts as a template to guide the polymerization of other extracellular fibers, we examined whether collagen deposition was dependent on the fibronectin isoforms. At a glance, the collagen pattern was clearly different in the EDA– matrices (Fig. 4I). As collagen organization and crosslinking determine the rigidity of the ECM, we used atomic force microscopy (AFM) to measure the micromechanical properties of decellularized matrices generated by TGFβ-treated MEFs; this revealed a significantly lower elastic modulus in matrices derived from EDA– fibroblasts (Fig. 4J). Therefore, we conclude that the presence of the EDA in fibronectin is necessary for activated fibroblasts to generate matrices with high alignment and rigidity.

### Deposition of fibronectin fibers is sensitive to the action of metalloproteinases in the absence of EDA

**Fig. 5:**
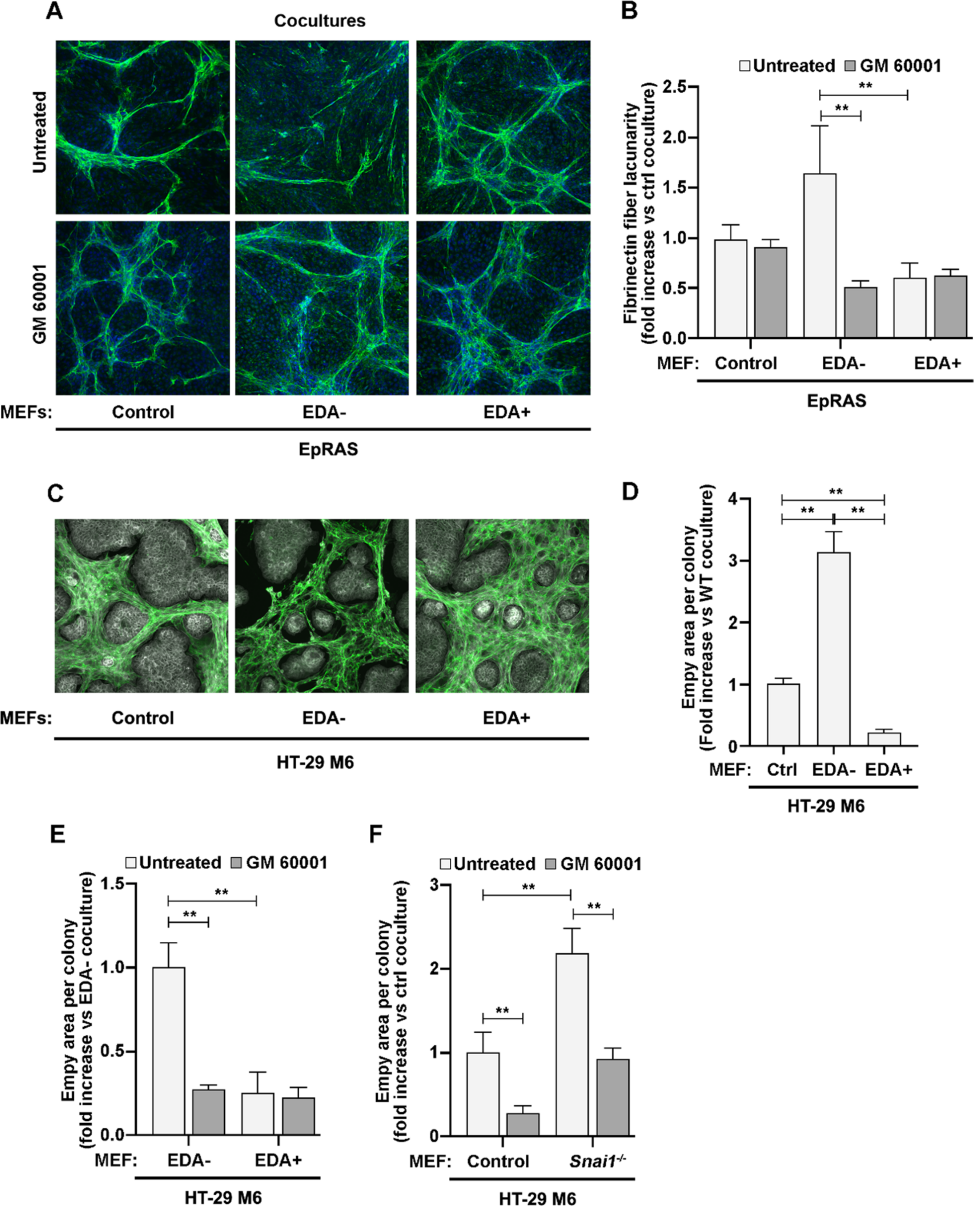
Matrices deposited from both EDA– fibronectin and *Snai1* KO MEFs are sensitive to metalloproteinases. **A** Fibronectin fiber organization in EpRas cells co-cultured with fibroblasts. EpRAs cells and the indicated MEFs were co-cultured on glass coverslips in the presence or absence of 25 µM GM6001 for 3 days. Co-cultures were analyzed by IF with anti-fibronectin (green) and DAPI (blue). Microscopy images are shown. **B** Fibronectin fiber lacunarity in EpRas co-cultured with fibroblasts is EDA and metalloproteinase dependent. Lacunary in fibronectin images obtained as in **A** was quantified using the TWOMBLI plugin of ImageJ software. The fold-increase with respect to values in untreated control MEF co-cultures is shown. **C** Fibronectin fiber organization in HT-29 M6 co-cultured with fibroblasts. Tumor cells and the indicated MEFs were co-cultured on glass coverslips for 6 days. Co-cultures were analyzed by IF with anti-fibronectin (green) and phalloidin (white). Microscopy images are shown. **D** HT-29 M6 colonies co-cultured with fibroblasts control the presence fibronectin around them in an EDA-dependent manner. For each HT 29 M6 colony, the perimeter and associated empty area (black surface) were quantified (ImageJ software) from images obtained as in **A**. The fold-increase of the “black area/perimeter” in each co-culture with respect to values in control MEF co-cultures is shown. **E** The metalloproteinase inhibitor GM6001 rescues the EDA-lacking fibronectin deposition around HT-29 M6 colonies**.** Cocultures of HT-29 M6 and indicated MEFs were carried out and imaged as in **C** in the presence or absence of the 25 μM GM6001. Black area measurements and plotting were carried out as in B. Fold increase with respect to values in untreated EDA– MEF co-cultures is shown. **F** The metalloproteinase inhibitor GM6001 rescues the lack of fibronectin accumulation around HT-29 M6 colonies co-cultured with *Snai1* KO MEFs. Co-cultures with indicated cells were established, treated and analyzed as in **C** and **D**. The fold-increase with respect to values in untreated control MEF co-cultures is shown

The ECM in tumors is deposited in the presence of tumor cells, and matrix-remodeling enzymes, such as metalloproteinases, are activated by signaling resulting from the interactions between tumor cells and fibroblasts [53]. Thus, we evaluated the fibronectin fiber organization in co-cultures of tumor cells and fibroblasts expressing different fibronectin isoforms. In the presence of pre-seeded, Ras-transformed EpH4 breast cells (EpRas), MEFs deposited fibronectin fiber around tumor cell patches visualized as a net of fibers in a fibronectin staining (Fig. 5A). Lacunas between fibers measured by TWOMBLI were larger in co-cultures with MEFs expressing EDA– fibronectin than with the other MEF lines (Fig. 5B). Similar differences were observed in co-cultures with other tumor cells, such as MCF7 (breast) or HepG2 (liver) (Additional file 1: Fig. S7A and S7B). TWOMBLI analyses also detected differences in fiber branchpoints between co-cultures (Additional file 1: Fig. S7C and S7D). Notably, addition of the metalloproteinase inhibitor GM6001 reverted the lacunarity and branchpoints parameters in co-cultures with EDA– fibronectin (Fig. 5A,B, Additional file 1: Fig. S7C).

We also analyzed co-cultures of the colon cancer cells, HT-29 M6, which (unlike other co-cultures) grow in highly compacted colonies that are isolated from fibroblasts. In co-cultures with EDA+ MEFs, colonies of tumor cells were mostly fully encircled by fibroblasts and their fibronectin fibers. In contrast, colonies in co-cultures with EDA– MEFs were surrounded by unoccupied spaces (Fig. 5C), which were estimated with ImageJ to be eight times larger than in colonies from EDA+ MEFs co-cultures (Fig. 5D). EDA– MEFs co-cultures had broken fibronectin fibers that were clearly visualized in three-dimensional reconstructions generated from confocal images (Additional file 1: Fig. S7E), and the empty area was strongly reduced when the metalloproteinase inhibitor GM6001 was included (Fig. 5E). Altogether, these observations indicate that the activity of metalloproteinases affects fibronectin fiber organization in an EDA-dependent manner. Correlating with the fact that *Snai1* KO MEFs express lower proportions of EDA+ fibronectin isoforms (Fig. 1), these MEFs also left an empty space around M6 colonies that was rescued by the MMP inhibitor when co-cultured (Fig. 5F).

### Fibronectin EDA facilitates tumor cell invasion

**Fig. 6:**
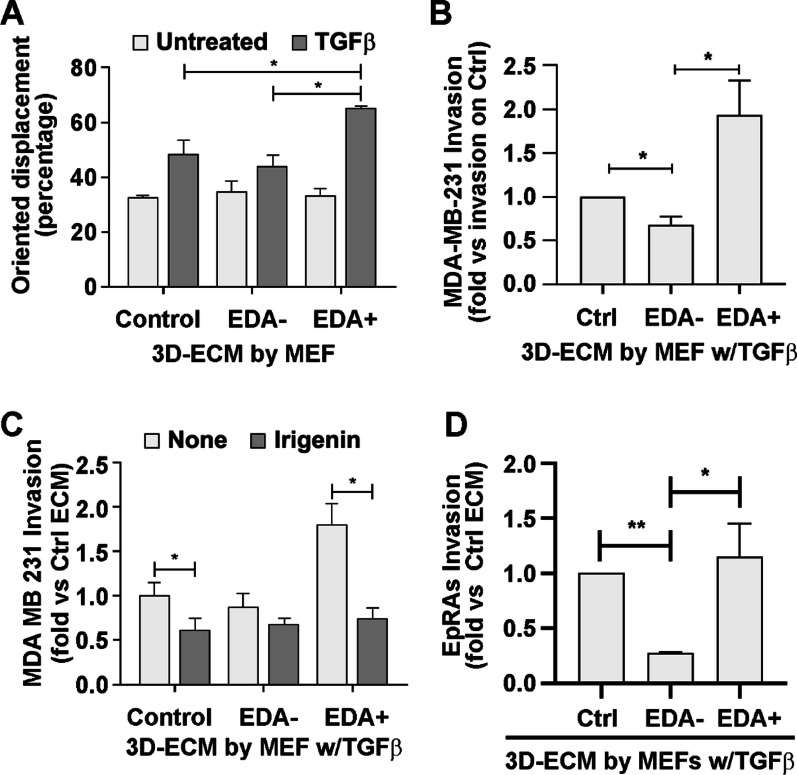
Tumor cell invasion is favored by the presence of EDA+ fibronectin in the 3D ECM.** A** MDA-MB-231 cell oriented migration depends on the presence of EDA+ fibronectin in the 3D ECM. Cell-tracker labeled MDA-MB-231 tumor cells were seeded on top of decellularized 3D ECMs generated by the indicated MEFs in the absence or presence of 5 ng/ml TGFβ_1_. Cell migration was recorded overnight by taking IF images every 15 min using life microscopy (Additional file 1: Fig. S8A). MDA cell movement was tracked using ImageJ software, and displacement features, such as the angle of each displacement, were measured. Oriented migration was plotted as the percentage of cell movements in the maximum orientation (up to 21˚ deviation from the mode). **B** MDA-MB-231 cell invasion is increased on 3D ECMs with EDA+ fibronectin. The indicated MEF lines were allowed to produce 3D ECMs in the presence of 5 ng/ml TGFβ_1_ on invasion-insert membranes. ECMs were decellularized, and MDA cells (in DMEM with 0.1% FBS) were seeded on top. DMEM with 10% FBS was added to the lower chamber as a chemoattractant. Cells were allowed to invade for 16 h and fixed with 4% PFA. Invading cells were stained with DAPI and quantified. **C** MDA-MB-231 cell invasion through EDA+ fibronectin matrices is interfered by irigenin treatment during matrix formation. MDA invasion through decellularized 3D ECMs produced by the indicated MEF lines activated with 5 ng/ml TGFβ_1_ and either treated or not with 50 μM irigenin, was quantified as in B. **D** EpRas invasion is increased on 3D ECMs containing EDA+ fibronectin. Invasion insert membranes were covered with indicated 3D ECMs as described in **B** and EpRas were induced to invade decellularized ECMs for 48 h and quantified as in **B**

We next took advantage that our 3D ECM can be decellularized to evaluate the activity of polymerized fibronectin fibers with or without EDA on tumor cell migration and invasion. We used MDA-MB-231 breast tumor cells, which move individually in culture (Additional file 1: Fig. S8A); we found that a higher percentage of these cells achieved oriented displacements on matrices generated by TGFβ-treated EDA+ MEFs as compared to matrices generated by TGFβ-treated EDA– MEFs or non-treated MEFs lines (Fig. 6A). Oriented movements recapitulate the alignment index of the matrices (Fig. 4G). Remarkably, an equivalent trend was obtained in invasion experiments through matrices deposited by these two TGFβ-treated MEF lines (Fig. 6B). Intermediate levels of oriented movements and invading MDA cells were obtained in matrices generated by TGFβ-treated control MEFs (Fig. 6A,B). The EDA requirement was challenged with irigenin, a small molecule that selectively binds to and blocks the EDA-integrin interaction [54]. We found that matrices generated in the presence of irigenin had a low tumor cell invasion, similar to that generated by EDA– MEFs (Fig. 6C). Irigenin treatment interfered with the orientation of the fibronectin fibers and the lacunarity of TGFβ-treated EDA+ matrices, as well with the oriented displacement of MDA cells on these matrices (Additional file 1: Fig. S8B).

We also tested EpRas cells, which formed colonies that moved collectively on decellularized matrices (Additional file 1: Fig. S8C). Of note, colonies on TGFβ-activated EDA+ matrices moved more compactly and coordinately than those on activated EDA– matrices (Additional files 2, 3, 4: S1, S2, and S3). Immunofluorescence analysis also illustrated the difference in compactness between EpRas colonies growing on each substrate (Additional file 1: Fig. S8D), and invasion assays showed that the coordinated collective movement of EpRas colonies on aligned and rigid fibronectin EDA+ matrices was accompanied by a higher capacity to invade the matrix (Fig. 6D).

### Matrix remodeling by TGFβ is required to stimulate fibroblast

**Fig. 7:**
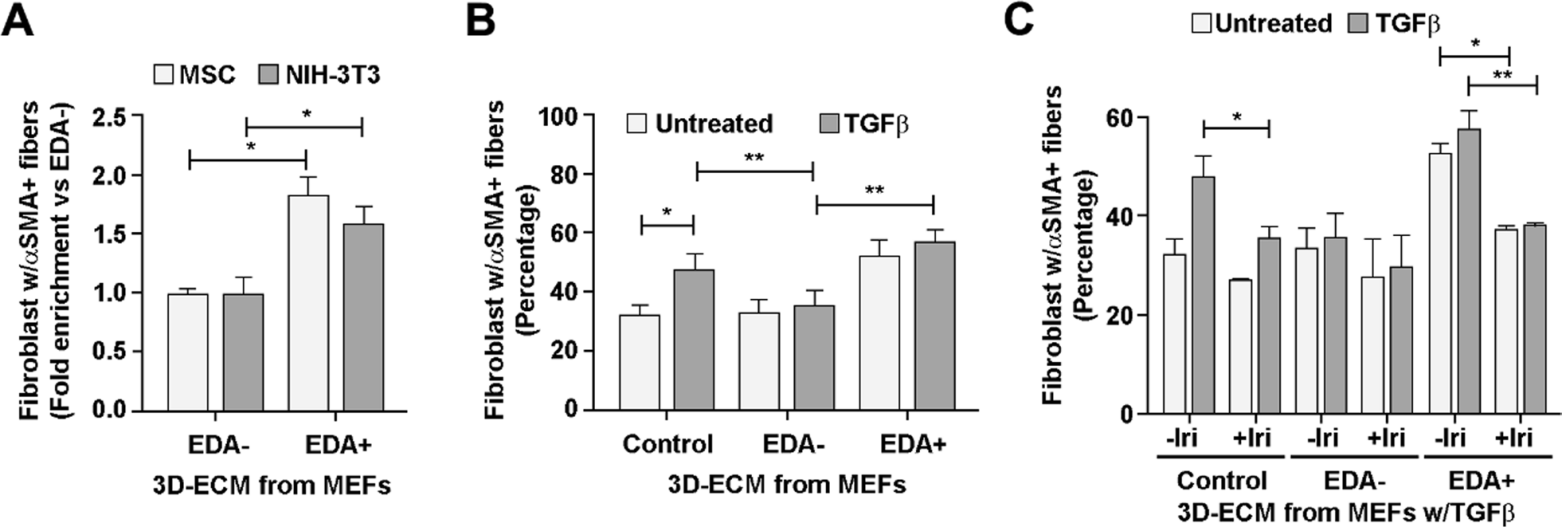
Matrices with EDA+ fibronectin induce the assemblage of α-SMA fibers in naïve fibroblasts. **A** MSC or NIH3T3 fibroblasts are induced to assemble α-SMA fibers by EDA+ matrices. MSC or NIH3T3 were grown 24 h on decellularized 3D ECMs generated by EDA– or EDA+ MEFs before detecting α-SMA and nuclei by IF. Images obtained through fluorescence microscopy were used to quantify the percentage of cells presenting α-SMA positive stress fibers. **B** NIH3T3 fibroblasts are induced to assemble α-SMA fibers by control and EDA matrices. NIH3T3 fibroblasts were grown 24 h on decellularized 3D ECMs generated by the indicated MEFs in the presence or absence of 5 ng/mL TGFβ_1_. The percentage of fibroblasts presenting α-SMA positive stress fibers was quantified as in **A**. **C** Irigenin interferes with fibroblast activation by EDA+ matrices. NIH3T3 fibroblasts were grown for 24 h on decellularized 3D ECMs generated by the indicated MEFs in the presence or absence of 5 ng/mL TGFβ_1_, and the presence or absence of 50 μM irigenin. The percentage of fibroblasts presenting α-SMA positive stress fibers was quantified as in **A**

Fibroblasts are activated by recombinant EDA fragments [31]. Here, we tested whether EDA in extracellular-deposited fibronectin fibers was also effective. Naïve fibroblasts were seeded on decellularized 3D ECMs, and the presence of cytosolic α-SMA stress fibers (a marker of fibroblast activation) was analyzed 16 h later. For both mouse mesenchymal stem cells (MSC) and NIH3T3 fibroblasts, the percentage of cells with α-SMA–positive fibers was higher on matrices derived from EDA+ MEFs than EDA– MEFs (Fig. 7A; Additional file 1: Fig. S9).

Matrices deposited by MEF EDA+ in either the presence or absence of TGFβ stimulated NIH3T3 (Fig. 7B); however, matrices deposited by control MEFs stimulated fibroblasts only if generated in the presence of the TGFβ cytokine (Fig. 7B). Given the elevated EDA+ fibronectin ratio generated and deposited by control MEFs (Figs. 1 and 4), our result suggests a TGFβ-induced conformational change that exposes polymerized EDA to fibroblasts.

To validate that stimulation of naïve fibroblasts depends on EDA in our experimental approach, fibroblast stimulation was challenged with irigenin. Addition of this compound prevented the formation of α-SMA–positive fibers on matrices deposited by TGFβ-treated EDA+ or control MEFs (Fig. 7C), further supporting the idea of an EDA-dependent stimulatory action on fibroblasts.

### The absence of fibroblastic EDA+ fibronectin expression in primary tumors prevents the formation of metastases

**Fig. 8:**
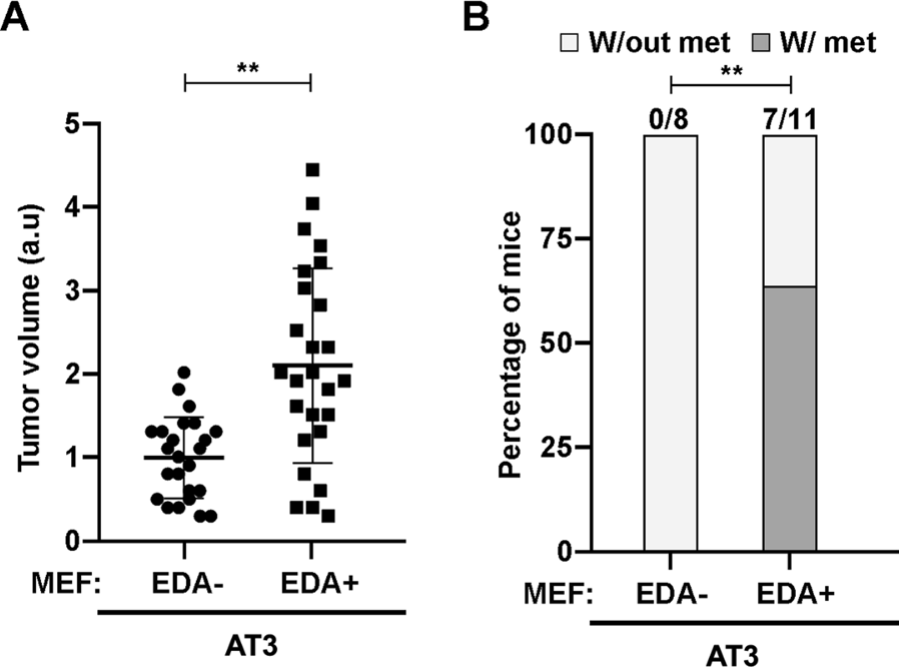
Fibroblasts lacking EDA fibronectin prevent metastasis formation. **A** AT3 coinjected with EDA+ MEFs generate bigger tumors than those with EDA– MEFs. Orthotopic tumors were generated in NOD-SCID gamma mice (Additional file 1: Fig. S10A). After resection, the three main dimensions of primary tumors were measured to calculate their volumes. The volume of each tumor relative to the average volume of EDA– MEF co-injected tumors is plotted. **B** Lung metastasis are absent in EDA– co-injected tumors. Lungs from injected mice in **A** were extracted 7 weeks after resection of primary tumors, fixed in 4% PFA and embedded in paraffin. H&E staining of lung slices to visualize metastasis are shown in the Additional file 1: Fig. S10B. The plot shows a quantification of the presence of metastatic foci obtained from lung H&E staining. Lungs with at least one metastasis are indicated as positive

Our results indicated that the EDA+ fibronectin is a critical structural element for the assembly of an invasion-permissive extracellular matrix. Therefore, we evaluated the action of EDA+ fibronectin in an orthotopic model of metastatic breast cancer. We injected AT3 tumor cells and EDA– or EDA+ MEFs into the inguinal mammary fat pads of NOD-SCID gamma mice. Tumors were monitored and resected simultaneously when they reached 0.2–0.4 cm of diameter. The volume of extracted tumors was accurately measured, and the tumors were then fixed for immunohistological analyses (Additional file 1: Fig. S10A). Tumors generated in the presence of EDA+ fibronectin–expressing MEFs were larger than those generated in the presence of EDA– fibronectin–expressing MEFs (Fig. 8A; Additional file 1: Fig. S10B).

After surgical resection of the primary tumors, mice were kept alive for 7 weeks to allow for the growth of lung metastasis. The lungs were then extracted, and hematoxylin and eosin staining was performed to study the presence of metastatic foci (Additional file 1: Fig. S10B). While over 60% of the animals injected with AT3 plus EDA+ MEFs developed metastatic foci, lungs of mice injected with AT3 plus EDA– MEFs were free of metastasis (Fig. 8B), clearly demonstrating the critical role of the fibronectin EDA in tumor malignant progression.

## Discussion

Expression of EDA+ fibronectin has been reported in advanced-stage tumors, including breast tumors; however, its direct role in determining stromal architecture and tumor progression remains partially unknown. For breast cancers, Femel and colleagues [55] were able to attenuate PYMT-induced breast cancer progression with a vaccination strategy against EDA+ fibronectin; however, the action of this treatment on the ECM architecture was not analyzed. Our data from three-dimensional matrices generated by TGFβ-activated MEFs, which were shown to mimic metastasis permissive matrices generated by myoCAF lines, indicate that the presence of EDA+ fibronectin favors the creation of an extracellular topology analogous to the permissive one. In contrast, EDA– fibronectin favors a restrictive extracellular net.

According to our observation, drugs targeting myoCAFs should block the incorporation of EDA+ fibronectin, but not of isoforms lacking EDA, into the matrix. Thus, considering that stabilized antisense oligonucleotides are clinically effective for diseases caused by defined splicing defects [56], the design of oligonucleotides that block the EDA inclusion event is an attractive new antitumor approach to address. However, information about which subtypes of breast tumors would be more sensitive is still missing, and interfering with fibronectin EDA splicing should be achieved before a permissive ECM is generated in the tumor stroma. Therefore, this approach may be appropriate for reducing the formation of a pro-metastatic environment in breast tumors expected to recur, for instance, in patients after either surgery or a first effective chemotherapeutic treatment, but that it may be inappropriate for treating patients with detected metastases. These observations are relevant as most clinical trials are conducted with patients with metastasis who would not benefit from this treatment, and because other therapeutic attempts to block CAF activity do not assess whether the treatment alters the deposition of restrictive fibronectin isoforms.

As fibrosis processes and the acquisition of chemoresistance have also been related to myofibroblast activity and ECM properties [57–59], our contribution to the differential activity of fibronectin isoforms and their regulation by SNAIL1 may also be useful for fine-tuning their current treatments. In addition to the pharmacological point of view, our results show functional and mechanistic aspects that should be taken into account.

In accordance with the capability of SNAIL1 to induce EDA inclusion, expression of the SNAIL1 and EDA+ fibronectin correlates in human advanced solid tumors. The available tumor data that we analyzed do not discriminate between an epithelial or mesenchymal origin of the molecules, but both molecules are reported to be expressed more frequently by fibroblasts than epithelial cells. *Snai1* KO and EDA– fibronectin MEFs behave similarly: They deposit matrices that do not sustain rigidity, fiber organization, metalloproteinase resistance, fibroblast activation or tumor cell invasion. Consequently, neither fibroblast line promotes lung metastasis in mouse breast tumors (Fig. 8B) [60]. Although we cannot completely rule out potential differences between the effects of the ECM produced by TGFβ-activated MEFs and myoCAFs, these data support that SNAIL1 is a component of the TGFβ/EDA+ fibronectin signaling loop involved in the assemblage of a stromal architecture that is favorable to the formation of metastasis [61].

The volume of primary breast tumors obtained in the presence of fibroblasts expressing EDA+ fibronectin was larger than of those expressing EDA– fibronectin. These data are in line with reports indicating that conditioned media from the EDA+ MEFs as well as from recombinant EDA+ fibronectin activate cell growth [30, 62]. However, we were not able to reproduce this outcome in tumor cell lines grown on 3D ECM with fibronectin either EDA– or EDA+ (data not shown). Hence, it is likely that the tumor size increase arises from soluble EDA+ fibronectin or other factors specifically secreted by EDA+ MEFs. Noteworthy, no volume differences were detected between PYMT-tumors generated in the presence of control or *Snai1* KO fibroblasts, even though *Snai1* KO fibroblasts block lung metastasis [60]. Given that we demonstrate that EDA+ fibronectin contributes to generating a stiff and fiber-aligned metastatic architecture that is TGFβ/SNAIL1-dependent, EDA+ fibronectin likely supports metastasis independently of increased tumor cell growth.

TGFβ-activated matrices from control and EDA+ MEFs are equivalent in terms of rigidity, fiber orientation and capacity to sustain invasion of collectively moving cells, indicating that EDA enrichment suffices to impose metastatic properties. However, while recombinant fibronectin fragments with EDA stimulate naïve fibroblasts [31], we found that matrices deposited by untreated control MEFs stimulate them as inefficiently as those deposited by EDA– MEFs. In contrast, we found that fibroblasts were efficiently stimulated by matrices from TGFβ-treated control MEFs; taken together, these results suggest that fiber alignment is required for the activation of EDA-dependent positive feedback on fibroblasts when the EDA+ and EDA– isoforms coexist, as is the case in the tumor stroma.

SR proteins, such as SRSF1, have been proposed to couple RNA Pol II transcription to pre-mRNA [47]. Our data linking SNAIL1 and SRSF1 fit with the existence of a TGFβ-induced co-transcriptional splicing complex with both proteins linked by nascent RNA. It is plausible that SNAIL1 is recruited to a canonical 5’-CACCTG binding site located just upstream of the enhancer splicing element that contains a consensus sequence for SRSF1 [63]. The presence of SNAIL1 in the exon may determine the conformation adopted by the nascent RNA and allow access of SRSF1 to its RNA binding site. The assembly of the splicing complex may be favored by additional TGFβ- or TGFβ/SNAIL1-dependent events, such as methylation or phosphorylation of its components [9, 34] or increased fibronectin transcription [64].

In addition to SRSF1, we observed that KHSRP, a splicing factor with binding sites around the exon 33, co-precipitated with SNAIL1. Despite this interaction, EDA inclusion was unaltered by lowering the levels of this factor with siRNAs (data not shown), indicating that this factor not involved in EDA splicing. Gain- and loss-of-function experiments in the absence of TGFβ suggest that SNAIL1 can modulate the alternative splicing of EDA by controlling the levels of other splicing factors, such as SRSF5 in MEFs and QKI in epithelial cells. However, EDA inclusion by SRSF1 is likely more relevant in myofibroblasts, given that the SNAIL1 levels are physiologically modulated by signaling pathways like TGFβ.

## Conclusions

Our results supports that EDA+ fibronectin isoforms favor the generation of an extracellular architecture similar to that generated by the myoCAF cell lines and that SNAIL1 controls the EDA inclusion into fibronectin. From a pharmacological point of view, specifically targeting EDA + fibronectin while leaving EDA– fibronectin isoforms alone could be a useful way to target the formation of a pro-metastatic environment in expected recurrent breast tumors, especially in tumors with limited alternative treatment, such as the triple-negative type.

### Supplementary Information

**Additional file 1.** Uncropped gels and blot images and standard protocols and specific reagents for: RNA extraction, reverse transcription and PCR, RNA-seq and alternative splicing analysis, Western blotting, immunofluorescence analysis, immunohistochemistry, collagen imaging, immunoprecipitation assay, chromatin immunoprecipitation, fibroblast activity, deposition of three-dimensional extracellular matrices, invasion assays and statistical analysis.

**Additional file 2: Video S1.** EpRas cell moving on glass coverslips. EpRas cells were plated on glass coverslips and 24 hlater were recorded overnight by taking images every 15 min with life microscopy.

**Additional file 3: Video S2.** EpRas cell moving on EDA– 3D ECMs. EpRas cells were plated on decellularized 3D ECM derived from MEFs EDA– treated with 5ng/mL TGFβ and 24 ho later were recorded overnight by taking images every 15 min with life microscopy.

**Additional file 4: Video S3.** EpRas cell moving on EDA+ 3D ECMs. EpRas cells were plated on decellularized 3DECM derived from MEFs EDA+ treated with 5ng/mL TGFβ and 24 h later were recorded overnight by taking images every 15 min with life microscopy.

## Data Availability

Gene expression data supporting the finding from this manuscript were included as Additional file 1: Tables S1 and S2.

## Abbreviations

3D ECM: Three-dimensional extracellular matrix
AFM: Atomic force microscopy
α-SMA: Alpha-smooth muscle actin
CAF: Cancer-associated fibroblast
ChIP: Chromatin immunoprecipitation
EDA: Extra domain A
ECM: Extracellular matrix
*FN1*: Fibronectin gene
KO: Knock out
MEF: Mouse embryonic fibroblast
MSC: Mesenchymal stem cell
myoCAF: MYOfibroblastic cancer-associated fibroblast
NOD-SCID: Non-obese diabetic-severe combined immunodeficiency
RT-PCR: Reverse transcription polymerase chain reaction
*Snail1*: Mouse gene for the transcription factor SNAIL1
SNAIL1: Protein corresponding to the transcripton factor 1 of the SNAG family
SRSF1: Serine/arginine-rich splicing factor 1
TGFβ: Transforming growth factor beta
TWOMBLI: The workflow of matrix biology informatics

## References

[1] da Silva-Diz V, Lorenzo-Sanz L, Bernat-Peguera A, Lopez-Cerda M, Muñoz P (2018). Cancer cell plasticity: impact on tumor progression and therapy response. Semin Cancer Biol.

[2] O’Brien-Ball C, Biddle A (2017). Reprogramming to developmental plasticity in cancer stem cells. Dev Biol.

[3] Delinassios JG, Hoffman RM (2022). The cancer-inhibitory effects of proliferating tumor-residing fibroblasts. Biochim Biophys Acta Rev Cancer.

[4] Conklin MW, Eickhoff JC, Riching KM, Pehlke CA, Eliceiri KW, Provenzano PP (2011). Aligned collagen is a prognostic signature for survival in human breast carcinoma. Am J Pathol.

[5] Takai K, Le A, Weaver VM, Werb Z (2016). Targeting the cancer-associated fibroblasts as a treatment in triple-negative breast cancer. Oncotarget.

[6] Wang M, Feng R, Chen Z, Shi W, Li C, Liu H, Wu K, Li D, Li X (2022). Identification of cancer-associated fibroblast subtype of triple-negative breast cancer. J Oncol.

[7] Van Cutsem E, Tempero MA, Sigal D, Oh DY, Fazio N, MacArulla T (2020). Randomized phase III trial of pegvorhyaluronidase alfa with nab-paclitaxel plus gemcitabine for patients with hyaluronan-high metastatic pancreatic adenocarcinoma. J Clin Oncol.

[8] Rahimi RA, Leof EB (2007). TGF-β signaling: A tale of two responses. J Cell Biochem.

[9] Sala L, Franco-Valls H, Stanisavljevic J, Curto J, Vergés J, Peña R (2019). Abrogation of myofibroblast activities in metastasis and fibrosis by methyltransferase inhibition. Int J Cancer.

[10] Stanisavljevic J, Loubat-Casanovas J, Herrera M, Luque T, Pena R, Lluch A (2015). Snail1-expressing fibroblasts in the tumor microenvironment display mechanical properties that support metastasis. Cancer Res.

[11] Francí C, Gallén M, Alameda F, Baró T, Iglesias M, Virtanen I (2009). Snail1 protein in the stroma as a new putative prognosis marker for colon tumours. PLoS ONE.

[12] Baulida J, de Herreros AG (2015). Snail1-driven plasticity of epithelial and mesenchymal cells sustains cancer malignancy. Biochim Biophys Acta BBA Rev Cancer.

[13] Graham J, Raghunath M, Vogel V (2019). Fibrillar fibronectin plays a key role as nucleator of collagen i polymerization during macromolecular crowding-enhanced matrix assembly. Biomater Sci.

[14] Mao Y, Schwarzbauer JE (2005). Fibronectin fibrillogenesis, a cell-mediated matrix assembly process. Matrix Biol.

[15] Patten J, Wang K (2021). Fibronectin in development and wound healing. Adv Drug Deliv Rev.

[16] Guan JL, Trevithick JE, Hynes RO (1990). Retroviral expression of alternatively spliced forms of rat fibronectin. J Cell Biol.

[17] Jarnagin WR, Rockey DC, Koteliansky VE, Wang SS, Bissell DM (1994). Expression of variant fibronectins in wound healing: cellular source and biological activity of the EIIIA segment in rat hepatic fibrogenesis. J Cell Biol.

[18] Kelsh RM, McKeown-Longo PJ, Clark RAF (2015). EDA fibronectin in keloids create a vicious cycle of fibrotic tumor formation. J Invest Dermatol.

[19] Muro AF, Chauhan AK, Gajovic S, Iaconcig A, Porro F, Stanta G (2003). Regulated splicing of the fibronectin EDA exon is essential for proper skin wound healing and normal lifespan. J Cell Biol.

[20] Phanish MK, Heidebrecht F, Nabi ME, Shah N, Niculescu-Duvaz I, Dockrell MEC (2015). The regulation of TGFβ1 induced fibronectin EDA exon alternative splicing in human renal proximal tubule epithelial cells. J Cell Physiol.

[21] Astrof S, Crowley D, George EL, Fukuda T, Sekiguchi K, Hanahan D (2004). Direct test of potential roles of EIIIA and EIIIB alternatively spliced segments of fibronectin in physiological and tumor angiogenesis. Mol Cell Biol.

[22] D’Ovidio MC, Mastracchio A, Marzullo A, Ciabatta M, Pini B, Uccini S (1998). Intratumoral microvessel density and expression of ED-A/ED-B sequences of fibronectin in breast carcinoma. Eur J Cancer.

[23] Pujuguet P, Hammann A, Moutet M, Samuel JL, Martin F, Martin M (1996). Expression of fibronectin ED-A+ and ED-B+ isoforms by human and experimental colorectal cancer: contribution of cancer cells and tumor-associated myofibroblasts. Am J Pathol.

[24] Schwager K, Villa A, Rösli C, Neri D, Rösli-Khabas M, Moser G (2011). A comparative immunofluorescence analysis of three clinical-stage antibodies in head and neck cancer. Head Neck Oncol.

[25] Frey K, Fiechter M, Schwager K, Belloni B, Barysch MJ, Neri D (2011). Different patterns of fibronectin and tenascin-C splice variants expression in primary and metastatic melanoma lesions. Exp Dermatol.

[26] Schliemann C, Wiedmer A, Pedretti M, Szczepanowski M, Klapper W, Neri D (2009). Three clinical-stage tumor targeting antibodies reveal differential expression of oncofetal fibronectin and tenascin-C isoforms in human lymphoma. Leuk Res.

[27] Ohnishi T, Hiraga S, Izumoto S, Matsumura H, Kanemura Y, Arita N (1998). Role of fibronectin-stimulated tumor cell migration in glioma invasion in vivo: clinical significance of fibronectin and fibronectin receptor expressed in human glioma tissues. Clin Exp Metastasis.

[28] Sottile J, Hocking DC (2002). Fibronectin polymerization regulates the composition and stability of extracellular matrix fibrils and cell-matrix adhesions. Mol Biol Cell.

[29] Manabe RI, Oh-e N, Maeda T, Fukuda T, Sekiguchi K (1997). Modulation of cell-adhesive activity of fibronectin by the alternatively spliced EDA segment. J Cell Biol.

[30] Manabe RI, Oh-e N, Sekiguchi K (1999). Alternatively spliced EDA segment regulates fibronectin-dependent cell cycle progression and mitogenic signal transduction. J Biol Chem.

[31] Shinde AV, Kelsh R, Peters JH, Sekiguchi K, Van De Water L, McKeown-Longo PJ (2015). The α4β1 integrin and the EDA domain of fibronectin regulate a profibrotic phenotype in dermal fibroblasts. Matrix Biol.

[32] Serini G, Ropraz P, Geinoz A, Borsi L, Zardi L, Gabbiani G (1998). The fibronectin domain ED-A is crucial for myofibroblastic phenotype induction by transforming growth factor-b1. J Cell Biol.

[33] Okamura Y, Watari M, Jerud ES, Young DW, Ishizaka ST, Rose J (2001). The extra domain A of fibronectin activates toll-like receptor 4. J Biol Chem.

[34] White ES, Sagana RL, Booth AJ, Yan M, Cornett AM, Bloomheart CA (2010). Control of fibroblast fibronectin expression and alternative splicing via the PI3K/Akt/mTOR pathway. Exp Cell Res.

[35] Muro AF, Caputi M, Pariyarath R, Pagani F, Buratti E, Baralle FE (1999). Regulation of fibronectin EDA exon alternative splicing: possible role of RNA secondary structure for enhancer display. Mol Cell Biol.

[36] Batlle R, Alba-Castellón L, Loubat-Casanovas J, Armenteros E, Francí C, Stanisavljevic J (2013). Snail1 controls TGF-β responsiveness and differentiation of mesenchymal stem cells. Oncogene.

[37] Lambies G, Miceli M, Martínez-Guillamon C, Olivera-Salguero R, Peña R, Frías C (2019). TGFβ-activated USP27X deubiquitinase regulates cell migration and chemoresistance via stabilization of Snail1. Cancer Res.

[38] Sun W, Duan T, Ye P, Chen K, Zhang G, Lai M (2018). TSVdb: A web-tool for TCGA splicing variants analysis. BMC Genom.

[39] Baulida J, Díaz VM, García de Herreros A (2019). Snail1: a transcriptional factor controlled at multiple levels. J Clin Med.

[40] Otero J, Navajas D, Alcaraz J. Characterization of the elastic properties of extracellular matrix models by atomic force microscopy. In: Methods in cell biology, vol. 156. New York: Academic Press; 2020. p. 59–83. 10.1016/bs.mcb.2019.11.016 32222227

[41] Yamashita M, Ogawa T, Zhang X, Hanamura N, Kashikura Y, Takamura M (2012). Role of stromal myofibroblasts in invasive breast cancer: Stromal expression of alpha-smooth muscle actin correlates with worse clinical outcome. Breast Cancer.

[42] Zhou L, Yang K, Andl T, Wickett RR, Zhang Y (2015). Perspective of targeting cancer-associated fibroblasts in melanoma. J Cancer.

[43] Velez DO, Tsui B, Goshia T, Chute CL, Han A, Carter H (2017). 3D collagen architecture induces a conserved migratory and transcriptional response linked to vasculogenic mimicry. Nat Commun.

[44] Ray A, Provenzano PP (2021). Aligned forces: origins and mechanisms of cancer dissemination guided by extracellular matrix architecture. Curr Opin Cell Biol.

[45] Hu C, Zhao Y, Wang X, Zhu T (2021). Intratumoral fibrosis in facilitating renal cancer aggressiveness: underlying mechanisms and promising targets. Front Cell Dev Biol.

[46] Jeong S (2017). SR proteins: binders, regulators, and connectors of RNA. Mol Cells.

[47] Das R, Yu J, Zhang Z, Gygi MP, Krainer AR, Gygi SP (2007). SR proteins function in coupling RNAP II transcription to Pre-mRNA splicing. Mol Cell.

[48] Bieberstein NI, Straube K, Neugebauer KM, Hertel K (2014). Chromatin immunoprecipitation approaches to determine co-transcriptional nature of splicing. Spliceosomal Pre-mRNA Splicing. Methods in molecular biology.

[49] Stanisavljevic J, Porta-de-la-Riva M, Batlle R, García de Herreros A, Baulida J (2011). The p65 subunit of NF-κB and PARP1 assist Snail1 in activating fibronectin transcription. J Cell Sci.

[50] Lim LP, Sharp PA (1998). Alternative splicing of the fibronectin EIIIB exon depends on specific TGCATG repeats. Mol Cell Biol.

[51] Liao KC, Chuo V, Fagg WS, Modahl CM, Widen S, Garcia-Blanco MA (2021). The RNA binding protein quaking represses splicing of the Fibronectin EDA exon and downregulates the interferon response. Nucleic Acids Res.

[52] Wershof E, Park D, Barry DJ, Jenkins RP, Rullan A, Wilkins A (2021). A FIJI macro for quantifying pattern in extracellular matrix. Life Sci Alliance.

[53] Saad S, Bendall LJ, Gottlieb DJ, Bradstock KF, Overall CM (2002). Cancer cell-associated fibronectin induces release of matrix metalloproteinase-2 from normal fibroblasts. Cancer Res.

[54] Amin A, Chikan NA, Mokhdomi TA, Bukhari S, Koul AM, Shah BA (2016). Irigenin, a novel lead from Western Himalayan chemiome inhibits Fibronectin-Extra Domain A induced metastasis in Lung cancer cells. Sci Rep.

[55] Femel J, Huijbers EJM, Saupe F, Cedervall J, Zhang L, Roswall P (2014). Therapeutic vaccination against fibronectin ED-A attenuates progression of metastatic breast cancer. Oncotarget.

[56] Dhuri K, Bechtold C, Quijano E, Pham H, Gupta A, Vikram A (2020). Antisense oligonucleotides: an emerging area in drug discovery and development. J Clin Med.

[57] Turner NA (2016). Inflammatory and fibrotic responses of cardiac fibroblasts to myocardial damage associated molecular patterns (DAMPs). J Mol Cell Cardiol.

[58] Bhattacharyya S, Tamaki Z, Wang W, Hinchcliff M, Hoover P, Getsios S (2014). Fibronectin EDA promotes chronic cutaneous fibrosis through toll-like receptor signaling. Sci Transl Med.

[59] Li Y, Randriantsilefisoa R, Chen J, Cuellar-Camacho JL, Liang W, Li W (2020). Matrix stiffness regulates chemosensitivity, stemness characteristics, and autophagy in breast cancer cells. ACS Appl Bio Mater.

[60] Alba-Castellón L, Olivera-Salguero R, Mestre-Farrera A, Peña R, Herrera M, Bonilla F (2016). Snail1-dependent activation of cancer-associated fibroblast controls epithelial tumor cell invasion and metastasis. Cancer Res.

[61] Klingberg F, Chau G, Walraven M, Boo S, Koehler A, Chow ML (2018). The ED-A domain enhances the capacity of fibronectin to store latent TGF-β binding protein-1 in the fibroblast matrix. J Cell Sci.

[62] Losino N, Waisman A, Solari C, Luzzani C, Espinosa DF, Sassone A (2013). EDA-containing fibronectin increases proliferation of embryonic stem cells. PLoS ONE.

[63] Tacke R, Manley JL (1995). The human splicing factors ASF/SF2 and SC35 possess distinct, functionally significant RNA binding specificities. EMBO J.

[64] Baulida J (2017). Epithelial-to-mesenchymal transition transcription factors in cancer-associated fibroblasts. Mol Oncol.

