# Supplementary material for: Formation of an invasion-permissive matrix requires TGFβ/SNAIL1-regulated alternative splicing of fibronectin
Source: Breast Cancer Res. 2023 Nov 14;25:143. doi: 10.1186/s13058-023-01736-y (PMC10647173; doi:10.1186/s13058-023-01736-y)
Supplement: Supplementary file 1 — Additional file 1. Uncropped gels and blot images and standard protocols and specific reagents for: RNA extraction, reverse transcription and PCR, RNA-seq and alternative splicing analysis, Western blotting, immunofluorescence analysis, immunohistochemistry, collagen imaging, immunoprecipitation assay, chromatin immunoprecipitation, fibroblast activity, deposition of three-dimensional extracellular matrices, invasion assays and statistical analysis. [file 13058_2023_1736_MOESM1_ESM.docx]

**SUPPLEMENTARY MATERIAL**

**SUPPLEMENTARY MATERIALS AND METHODS**

**Stable cell lines**

Cells were grown and maintained in Dulbecco’s modified Eagle’s medium (DMEM) (Invitrogen) high glucose supplemented with 10% FBS (Gibco), 2 mM glutamine, 4,5 g/L of Sodium Pyruvate, 56 U/L penicillin, 56 U/L streptomycin, and non-essential amino acids. Cell cultures were maintained at 37 °C in a humid atmosphere containing 5% CO2.

MDA-MB-231, MCF7, HT-29 M6 and NIH-3T3 cells were acquired from the repository stock of our center. Mouse embryonic fibroblasts (MEFs) control and *Snai1* KO, mesenchymal stem cells (MSC) (1), and murine CAFs control and *Snai1* KO (2) were previously established in our laboratory. MSC are used as non-transformed fibroblasts recruited by tumors.

Mouse embryonic fibroblasts wild-type, EDA- and EDA+ were kindly provided by Dr. Andrés Muro lab, International Centre for Genetic Engineering and Biotechnology. BJ human fibroblasts were kindly gifted by Dr. Cristina Peña lab, Hospital Universitario Puerta de Hierro. EpRas tumor cell line was kindly provided by Dr. Antoni Celià lab, Institut Hospital del Mar d’Investigacions Mèdiques. They were originally generated by Dr. Robert Weinberg’s lab, Whitehead Institute for Biomedical Research. AT-3 tumor cell line was kindly gifted by Dr. José Yelamos’ lab, Institut Hospital del Mar d’Investigacions Mèdiques.

**Cell treatments**

Cells were treated with different specific reagents, which are listed in Table 1.

| Treatment | Supplier | Concentration |
| --- | --- | --- |
| TGFβ1 | Peprotech | 5ng/mL |
| Irigenin | Tebu Bio | 50μM |
| CLI-095 | InvivoGen | 5μM |
| GM6001 | Millipore | 25μM |
| CellTracker  Green CMFDA | Thermo Fisher | 1μM |

**Table 1: Cell culture treatments**

**Cell transfection**

For transient transfection with siRNA, MEFs were grown to 60–80% confluence. Cells were transfected in DMEM without antibiotics using the DharmaFECT transfection agent. Transfected cells were kept in media without antibiotics for 24 h and then complete medium for an extra 24 h before testing gene expression by RT-qPCR or 48 for protein analysis by WB. Transfected siRNAs are specified in Table 2.

| siRNA | Supplier |
| --- | --- |
| siCtrl | D-001810-02-50, Dharmacon |
| siSnai1 | L-010847-01-0005, Dharmacon |

**Table 2: Transfected siRNA**

**Cell infection**

Retrovirus containing a pBABE empty and pBABE *Snai1*-HA plasmid were used to induce stable expression of SNAIL1-HA in MEF cells. MEF were grown up to 50% confluence and virus were added dropwise to complete medium. Culture medium was renewed 24 h after infection and puromycin 1mg/mL was added to select infected cells.

**RNA extraction, reverse transcription, and PCR**

Cells were washed twice with cold PBS and then lysed in TRIzol reagent (Invitrogen). Lysates were mixed with 200 μL chloroform, vortexed and incubated for 2 min. Then, samples were centrifuged at 12000 g for 20 min at 4 ˚C. The aqueous phase of the samples was transferred to a new Eppendorf tube, mixed with 400 μL isopropanol and incubated for 20 min at Room Temperature (RT) to precipitate RNA. Then, tubes were centrifuged at 12000 g for 20 min at 4 ˚C. Supernatant was discarded and the pellet was washed with 70% ethanol and centrifuged at 12000 g for 15 min at 4 ˚C. Ethanol was completely removed and the RNA pellet was resuspended in 20-50 μL water. Extraction results were quantified using a NanoDrop.

Reverse transcription was performed on 1-2 mg RNA using the Transcription First Strand cDNA Synthesis Kit (Roche) following the manufacturer protocol.

PCR was performed using BioTaq DNA polymerase (Bioline) with specific oligonucleotides (Table 4) and 100 ng cDNA. For semiquantitative PCR, PCR cycles were optimized from 20 to 30 to better visualize differences between samples. PCR products were separated by 2% agarose DNA electrophoresis and visualized using SYBR Safe DNA gel stain (Invitrogen). The intensity of the amplified bands was estimated with the ImageJ gel analysis tool.

| Oligonucleotide | Sequence |
| --- | --- |
| FN1 exon 32 | CCCTGGTTCAAACTGCAGTG |
| FN1 exon 34 | GGTTGATTTCTTTCATTGGTCCTG |

**Table 4: Oligonucleotides used for PCR**

For quantitative studies, qPCR was performed using the SYBR Green LightCycler 480 Real Time System (Roche). 5 to 20 ng cDNA where amplified using specific oligonucleotides (Table 5).

| Oligonucleotide | Sequence |
| --- | --- |
| Human FN1 exon 33 Fw | TGCACGATGATATGGAGAGC |
| Human FN1 exon 34 Rv | TGGGTGTGACCTGAGTGAAC |
| Human FN1 exon 1 Fw | GGGAGCCTCGAAGAGCAAG |
| Human FN1 exon 2 Rv | CGCTCCCACTGTTGATTTATCTG |
| Human and Mouse HPRT Fw | GGCCAGACTTTGTTGGATTTG |
| Human and Mouse HPRT Rv | TGCGCTCATCTTAGGCTTTGT |
| Mouse KHSRP Fw | GACTCAGGCTGCAAAGTTCA |
| Mouse KHSRP Rv | GTGCTCCAGTCAGAGACACG |
| Mouse FN1 exon 32 Fw | CCCTGGTTCAAACTGCAGTG |
| Mouse FN1 exon 33 Rv | TGTGGGCTTTCCCAAGCAAT |
| Mouse FN1 Promoter Fw | CTGCTCTTGGGGCTCAACC |
| Mouse FN1 Promoter Rv | AAGGAGATGGAAGGAGAGGACC |
| Mouse FN1 exon 7 Fw | GCTCCTTCACTGATGTCCGAA |
| Mouse FN1 exon 7 Rv | CTTCAGCCACTGCATTCCCA |
| Mouse FN1 exon 33 Fw | TTCCAATCAGGGGCTGGCTCTC |
| Mouse FN1 exon 33 Rv | TCGAGCCCTGAGGATGGAATCC |
| Mouse FN1 intron 32 Rv | GCAGAACTGCTTTGCATGGTA |

**Table 5: Oligonucleotides used for Qpcr**

**RNA sequencing and alternative splicing analysis**

Total RNA was extracted from cultured cells at the indicated conditions with the GenElute Mammalian Total RNA Miniprep kit (Sigma). Samples were paired-end sequenced in CRG Sequencing Unit until reaching 80M reads/sample. For splicing analysis, two softwares were used. The SANJUAN (<https://github.com/ppapasaikas/SANJUAN>) was run with a threshold of 0.15 delta Percentage Spliced In, calculated from the number of inclusion and exclusion sequencing reads as follows: [#inclusion/(#inclusion+#exclusion)x100]. The SUPPA2 software was designed by Dr. Eduardo Eyras’ lab (<https://github.com/comprna/SUPPA>) was run with a threshold of 0.1 ΔPSI.

**Western Blot**

Cells were washed twice with cold PBS and then lysed with lysis buffer (2% SDS, 50mM TRIS pH 7.5, 10% glycine). Lysates were boiled for 10 min and centrifuged for 10 min at top speed to eliminate insoluble debris. Protein concentration was quantified by DC Protein Assay (Bio-Rad). For extraction of proteins from PDX frozen pieces, a volume of lysis buffer proportional to the piece weight was added and an 18G syringe was used to break the tissue. Then, the samples were boiled at 95 ˚C for 10 min. Afterwards, the samples were sonicated (Branson DIGITAL Sonifier UNIT Model S-450D) for two rounds of 15 seconds at a potency of 15%, with 30 seconds of resting between each round. The samples were kept on ice during all the process. Finally, we centrifuged the samples for 5 min at maximum revolution and collected and quantified the supernatant.

From 1 to 20μg protein were mixed with loading buffer (50mM TRIS pH6.8, 2% SDS, 10% glycerol, 0.1% bromophenol blue), boiled and loaded into an SDS-polyacrylamide gel. Samples were run in TGS buffer at 120 V and transferred into a nitrocellulose membrane at 400mA for 90 min. Once proteins were transferred, membrane was blocked with TBS-Tween 20 (Tris-buffered saline) 1% BSA and incubated overnight with the primary antibody diluted in TBST 0.1% BSA (Table 6). After three washes with TBST membranes were incubated with Horseradish peroxidase (HRP)-conjugated secondary antibody 1h at room temperature and washed again with TBS-Tween. The detection was carried out using Immobilon western HRP substrate (Millipore) and captured using the Alliance Q9 Advanced (Uvitec) chemiluminescence imager. For PDXs samples, the intensity of the SNAIL1 and fibronectin EDA bands was estimated with the ImageJ gel analysis tool.

| Protein | Host | Application | Dilution | Reference |
| --- | --- | --- | --- | --- |
| EDA+FN1 | Mouse | WB | 1:500 | ab6328, Abcam |
| EDA+FN1 | Mouse | WB / IF | 1:500 / 1:100 | F6140, Sigma |
| FN1 | Rabbit | WB / IF | 1:2000 / 1:1000 | A0245, Dako |
| LAMIN B | Rabbit | WB | 1:2000 | ab16048, Abcam |
| PYRUVATE KINASE | Goat | WB | 1:2000 | AB1235, Chemicon |
| SNAIL1 | Rabbit | WB | 1:1000 | 3879, Cell Signaling |
| SNAIL1 | Mouse | IF | 1:2 | Hybridoma(3) |
| SRSF1 | Mouse | WB / IF | 1:1000 / 1:100 | 32-4500, ThermoFisher |
| α-SMA | Mouse | IF | 1:100 | A2547, Sigma |
| β-ACTIN | Mouse | WB | 1:10000 | A5441, Sigma |
| α-TUBULIN | Mouse | WB | 1:10000 | T9026, Sigma |

**Table 6: Antibodies used for Immunofluorescence and Western Blot**

**Immunofluorescence analysis**

Cells were grown for at least 48 h on ethanol-sterilized glass coverslip following a standard IF protocol. All steps were carried out at room temperature. Cells were fixed with 4% PFA for 10 min. PFA autofluorescence was quenched by incubating with 50 mM NH4Cl in PBS for 5 min. Blocking and permeabilization were carried out at the same time with a solution of 1% BSA and 0,3% Triton X-100 for 1 hour. Coverslips were incubated overnight with specific primary antibodies (Table 6) in blocking + permeabilization solution, and then for 1 hour with the corresponding secondary antibody. In samples where phalloidin was used, it was added to the secondary antibody solution. Secondary antibodies and phalloidin were complexed with Alexa fluorochromes. Nuclei were stained with DAPI for 10 min and coverslips were mounted with Fluoromount G (Southern Biotech).

For 3D-ECM produced *in vitro*, the same protocol was followed. For nuclei alignment, measurements of the angle of the ellipse fitting with each nucleus were determined with ImageJ on DAPI images. Length, width and area of the ellipses were used for nucleus morphological analyses. Nucleus aspect ratio (AR) is defined as the ratio between nuclear length and width. Fibronectin fiber alignment was quantified on fibronectin immunofluorescent images using two ImageJ extensions. OrientationJ distribution tool of the OrientationJ plugin (4) was used setting both Min. Coherency and Min. Energy at 10%. The TWOMBLI plugin (5) allows quantifying other morphological patterns of the ECM, including the alignment. Optimal parameters to analyze our images were obtained by testing a small sample (Contrast Saturation: 0.35, Min Line Width: 10, Max Line Width: 10, Min Curvature Window: 50, Max Curvature Window: 50, Minimum Branch Length: 10, Maximum Display HDM: 225, Minimum Gap Diameter: 0).

**Collagen imaging**

Fixed cellularized 3D-ECMs produced in vitro were stained with Trichrome III blue staining kit (Roche). Alternatively, fixed cellularized 3D-ECM produced in vitro were used to obtain second harmonic generation (SHG) images. The second harmonic was detected with an inverted multiphoton laser scanning microscope (Leica TCS SP5) equipped with a pulsed (80 Mhz) and tunable Mai Tai Ti:Sapphire laser (Spectra Physics) set at 880 nm. To collect the SHG signal from collagen, a 0.55 NA condenser with a BP 436/7 filter set above it was used.

**Immunohistochemistry**

Harvested tissue samples were fixed in 4% PFA and embedded in paraffin. Sections of 4μm were obtained with a microtome and then subsequently dewaxed and rehydrated. Antigens were retrieved by boiling the samples in Tris/EDTA (50mM Tris/HCl, 1mM EDTA, and 10mM NaCl, pH 9.0) for 15 min. Endogenous peroxidase activity was quenched for 15 min with 3% hydrogen peroxide in PBS containing 1% sodium azide. After several rinses with PBS, sections were incubated with PBS containing 1% BSA and 0.3% Triton X-100 to block non-specific binding and then washed with PBS. Sections were incubated with the indicated antibodies overnight at 4 °C. After several rinses with PBS, bound antibody was detected using anti-mouse or anti-rabbit Envision. Sections were counterstained with hematoxylin and mounted for microscopy analysis.

**Immunoprecipitation Assay**

Cells grown at approximately 80% confluence were washed twice with cold PBS and then lysed with RIPA buffer (1% NP-40, 0.5% Sodium deoxycholate, 0.1% SDS in PBS). Protein concentration was quantified by DC Protein Assay (Bio-Rad). 500μg of protein was diluted in a total volume of 500μL and either 1μL of primary antibody or the corresponding volume of Irrelevant IgG was added. After incubating the mix overnight at 4 °C, 20μL of Gammabind G Sepharose (GE Healthcare) beads were added to each sample and incubated for 2 h at 4 °C. Samples were centrifuged at 380 g at 4 °C. Supernatants were discarded or saved as an unbound fraction to assess immunoprecipitation efficiency. Beads were washed with PBS 0.1% NP-40 and centrifuged three times. Finally, 20μL of Loading Buffer were added to the beads, boiled and samples were loaded in SDS-polyacrylamide gels for protein electrophoresis and western blot analysis.

**Chromatin Immunoprecipitation**

Cells were seeded on culture dishes and allowed to grow in regular culture medium up to 80% confluence and, when indicated, treated with 5ng/mL TGFβ for 3 h. Samples were cross-linked for 10 min at 37 °C with 1% formaldehyde in DMEM. To stop the reaction, cells were incubated for 5 more min with glycine added at a final concentration of 0.125M. Cells were washed twice with cold PBS and scrapped off with cold PBS containing Protease Inhibitors. Samples were centrifuged at 800 g for 5 min at 4 ˚C and supernatants were removed. Cell pellets were resuspended in soft lysis buffer (20mM Tris pH 8.1, 85mM KCl, 0.5% NP-40, 5mM PIPES) at a rate of 500μL per 10^7^ cells. Lysates were incubated for 15 min on ice and then centrifuged for 5 min at 8 g at 4 ˚C and the supernatants were discarded. Pellets were resuspended in nuclear lysis buffer (1% SDS, 10mM EDTA, 50mM Tris pH 8.1) and sonicated 15 rounds of 10 seconds (separated by 30 cooling seconds on ice) using 10% of the sonifier’s amplitude (Branson DIGITAL Sonifier UNIT Model S-450D) in order to generate DNA fragments ranging from 200 to 500 base pairs in length. Optionally, the length of the fragments was confirmed in a small volume of the sample by 2% agarose DNA electrophoresis. Samples were centrifuged at 12000 g for 10 min at 4˚C and the supernatant was recovered.

As starting material for the next steps of the procedure, a supernatant volume corresponding to 2x10^6^ cells was diluted 1:10 in dilution buffer (0.01% SDS, 1% Triton X-100, 16.7mM Tris pH 8.1, 1.2mM EDTA, 167mM NaCl). In order to reduce background, samples were incubated in constant rotation for 1 hour at 4 °C with IgGs of the same species as the primary antibody that is going to be used and 30μL Gammabind G Sepharose beads. Beads were typically separated from the samples by 3 min 350 g centrifugation at 4 °C.

10% of the beads free lysate was kept apart for the input and the remaining volume was cut in half and incubated overnight at 4 °C with agitation with either 5-10μL of the specific antibody or the equivalent amount of IgG of the same species. In parallel, 30μL of beads for each sample were blocked overnight with BSA 0,5% in TBS. The next day, beads were washed with dilution buffer and added to samples, which were further incubated 4 h at 4 °C with rotation. Afterwards, three washes were performed on ice with each of the given buffers: low salt buffer (0.1% SDS, 1% Triton X-100, 2mM EDTA, 20mM Tris pH 8.1, and 150mM NaCl), high salt buffer (the same as low salt but 500mM NaCl) and LiCl Buffer (250mM LiCl, 1% Nonidet P-40, 1% Sodium deoxycholate, 1mM EDTA, and 10mM Tris pH 8.0). For each wash, samples were centrifuged at 350 g for 3 min at 4 ˚C.

Recovered beads were incubated with 800 rpm shacking in 100μL elution buffer (100mM Na2CO3, 1% SDS) at 37 °C for 1 hour, separated by centrifugation and discarded. NaCl was added to elutes and inputs at a final concentration of 200mM. Immunoprecipitates and inputs were then decrosslinked by incubation at 65 °C overnight with 800 rpm shacking followed by digestion with proteinase K for 1 hour at 55 ˚C with shacking 800 rpm. DNA for quantitative PCR analysis was purified using the MinElute PCR Purification Kit (Qiagen).

**RNA Immunoprecipitation**

Cells on cell culture dishes were grown until 80% confluence, washed with warm PBS, trypsinized and recovered by centrifugation. Cells were then resuspended in 300μL lysis buffer (100mM KCl, 5mM MgCl2, 10mM HEPES pH 7, 0.5% NP-40, 1mM DTT, 1x RNase Inhibitor, 1x Protease Inhibitor) for each 15 cm diameter plate, incubated 5 min on ice, snap-frozen in liquid nitrogen. Thawed samples were sonicated in a Bioruptor Pico Sonicator (Diagenode) for 15 cycles of 30 seconds ON/OFF at 4 ˚C. Samples were centrifuged at 16000 g for 10 min at 4 ˚C, supernatants recovered, and their protein content quantified.

In parallel, antibody-complexed beads were prepared. 30μL of Gammabind G Sepharose beads per sample were washed in NET buffer (50mM Tris pH 7.5, 150mM NaCl, 0.1% NP-40, 1mM EDTA), blocked with 20μg tRNA, washed again and incubated for 2 h at 4 °C with rotation after adding the primary antibody or Irrelevant IgG. All centrifugations of beads were done at 350 g and 4 ˚C.

Protein samples were precleared with unblocked beads for 30 min at 4 °C with rotation, centrifuged for 2 min at 350 g and the supernatant was recovered. 6mg of protein was mixed with the previously blocked and Antibody-complexed beads and incubated for 2 h at 4 ˚C with rotation. The mix was centrifuged and beads were washed 4 times with NET buffer. Finally, beads were resuspended in 50μL NET with 2μL of blue glycogen and 150μL TRIzol and RNA extraction was carried out as described.

**Fibroblast activity**

40.000 MSC or NIH3T3 fibroblasts were seeded on decellularized matrices blocked with heat-denatured 2% BSA. Samples were fixed with 4% PFA and NH4Cl 50mM after an overnight (~16 h) to perform immunofluorescence with an α-SMA antibody. When required, inhibitors were added to the fibroblasts.

**Migration assays**

Prior to the assay, tumor cells were labeled in 80% confluence 10 cm round plates with 1μM Cell Tracker in DMEM medium with 0% FBS for one hour and allowed to recover in DMEM containing 10% FBS for half an hour. Next, 104 trypsinized cells were seeded on decellularized matrices blocked with heat-denatured 2% BSA. After at least 24 h, a fluorescence microscope Zeiss Cell Observer HS was used to obtain bright-field and fluorescence images every 15 min for ~16 h.

**Deposition of three-dimensional extracellular matrices**

Three-dimensional ECMs were generated following a previously described protocol (6). For 24 wells plates, 1-3x10^5^ fibroblasts were seeded on gelatin-cross-linked glass coverslips, and for invasion experiments, 10^5^ fibroblasts were seeded in gelatin-cross-linked invasion inserts, using 100 µL of medium (7). After 24 h, cell culture media were supplemented with 50µg/ml ascorbic acid and, where indicated, 5ng/ml TGFβ. To foster ECM deposition by the plated cells, media were replaced every two days for six days. Cultures were eventually washed with pre-warmed (37 °C) phosphate-buffered saline (PBS), and either fixed with 4% paraformaldehyde (PFA) and treated with NH_4_Cl 50mM to quench PFA fluorescence for immunofluorescence analysis, or decellularized with 20mM NH_4_OH and 0.5% Triton X-100 in PBS for later use as a cell culture substrate.

**Invasion assays**

Approximately 5x10^5^ cells were seeded on decellularized matrices produced in invasion inserts in 100μL of DMEM containing 0.1% FBS. DMEM (500μL, 10% FBS) was added to the lower chamber as a chemoattractant. MDA-MB-231 cells were allowed to invade the ECM for 24 h and EpRas were allowed to invade for 48 h. After the invasion period, samples were fixed with 4% PFA and stained with DAPI. Non-invading cells were removed from the upper side of the insert using a cotton swab. Invasion insert membranes were removed and mounted on glass slides using DAPI Fluoromount G (SouthernBiotech). Invading cells were imaged and quantified using Image J software.

**Mammary orthotopic transplantation and resection**

Animals were maintained in a specific pathogen-free area and fed *ad libitum*. All the procedures were approved by the Animal Research Ethical Committee from the Parc de Recerca Biomèdica de Barcelona (Barcelona, Spain) and by the Generalitat de Catalunya.

Synchronized primary tumors were generated in mice by implanting 5x10^4^ AT-3 tumor cells and 5x10^4^ MEF EDA+ or EDA- embedded in Matrigel. NOD-SCID (Non Obese Diabetic/Severe Combined Immunodeficiency) females at least eight weeks old were treated with buprenorphine 0.1 mg/kg and anesthetized with isoflurane 2.5-3.5% on O_2_ 0,8 L/min, following the procedure approved by the ethical committee, and two inguinal mammary fat pats per mice were injected. Tumor sizes were externally monitored and tumors were surgically resected when reached 0.2-0.4cm. For resection surgery, mice were anesthetized as described above. Post-resection mice were maintained alive an extra two months to allow for the growth of metastasis and then sacrificed to quantify tumor lung metastatic foci. Metastases were counted in hematoxylin and eosin stained slides of formalin fixed paraffin embedded lungs.

**REFERENCES**

1. Batlle R, Alba-Castellón L, Loubat-Casanovas J, Armenteros E, Francí C, Stanisavljevic J, et al. Snail1 controls TGF-β responsiveness and differentiation of mesenchymal stem cells. Oncogene [Internet]. 2013 [cited 2014 Jan 27];32:3381–9. Available from: http://www.pubmedcentral.nih.gov/articlerender.fcgi?artid=3494751&tool=pmcentrez&rendertype=abstract

2. Bruch-Oms M, Olivera-Salguero R, Mazzolini R, del Valle-Pérez B, Mayo-González P, Beteta Á, et al. Analyzing the role of cancer-associated fibroblast activation on macrophage polarization. Mol Oncol. 2023;17:1492–513.

3. Francí C, Takkunen M, Dave N, Alameda F, Gómez S, Rodríguez R, et al. Expression of Snail protein in tumor-stroma interface. Oncogene [Internet]. 2006 [cited 2013 Oct 21];25:5134–44. Available from: http://www.ncbi.nlm.nih.gov/pubmed/16568079

4. Püspöki Z, Storath M, Sage D, Unser M. Transforms and Operators for Directional Bioimage Analysis: A Survey. Focus Bio-Image Informatics. 2016. page 69–93.

5. Wershof E, Park D, Barry DJ, Jenkins RP, Rullan A, Wilkins A, et al. A FIJI macro for quantifying pattern in extracellular matrix. Life Sci Alliance. 2021;4:1–11.

6. Castelló-Cros R, Cukierman E. Stromagenesis During Tumorigenesis: Characterization of Tumor-associated Fibroblasts and Stroma-derived 3D Matrices. Extracell matrix Protoc. 2009. page 275–305.

7. Sala L, Franco-Valls H, Stanisavljevic J, Curto J, Vergés J, Peña R, et al. Abrogation of myofibroblast activities in metastasis and fibrosis by methyltransferase inhibition. Int J Cancer. 2019;

**SUPPLEMENTARY FIGURES**

**Supplementary Figure S1**

**
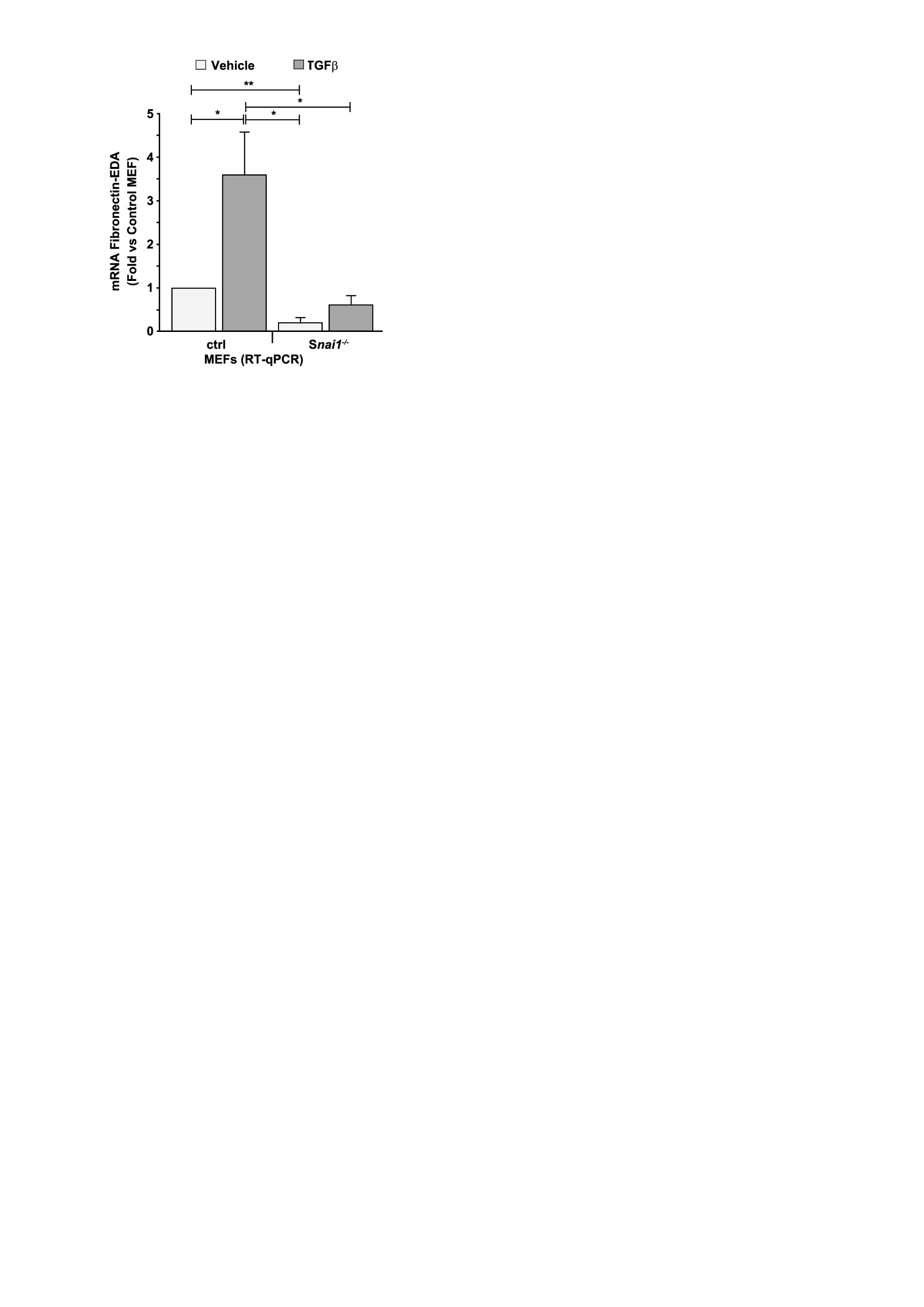
**

**Quantification of EDA fibronectin inclusion. A**, Quantification of EDA fibronectin inclusion by RT-qPCR. RNA obtained from Control or *Snai1*^-/-^ MEFs untreated or treated 24 h with 5ng/mL of TGFβ was retrotranscribed and amplified by quantitative PCR using primers specific for the exon 33 and HPRT for normalization.

**Supplementary Figure S2**

**
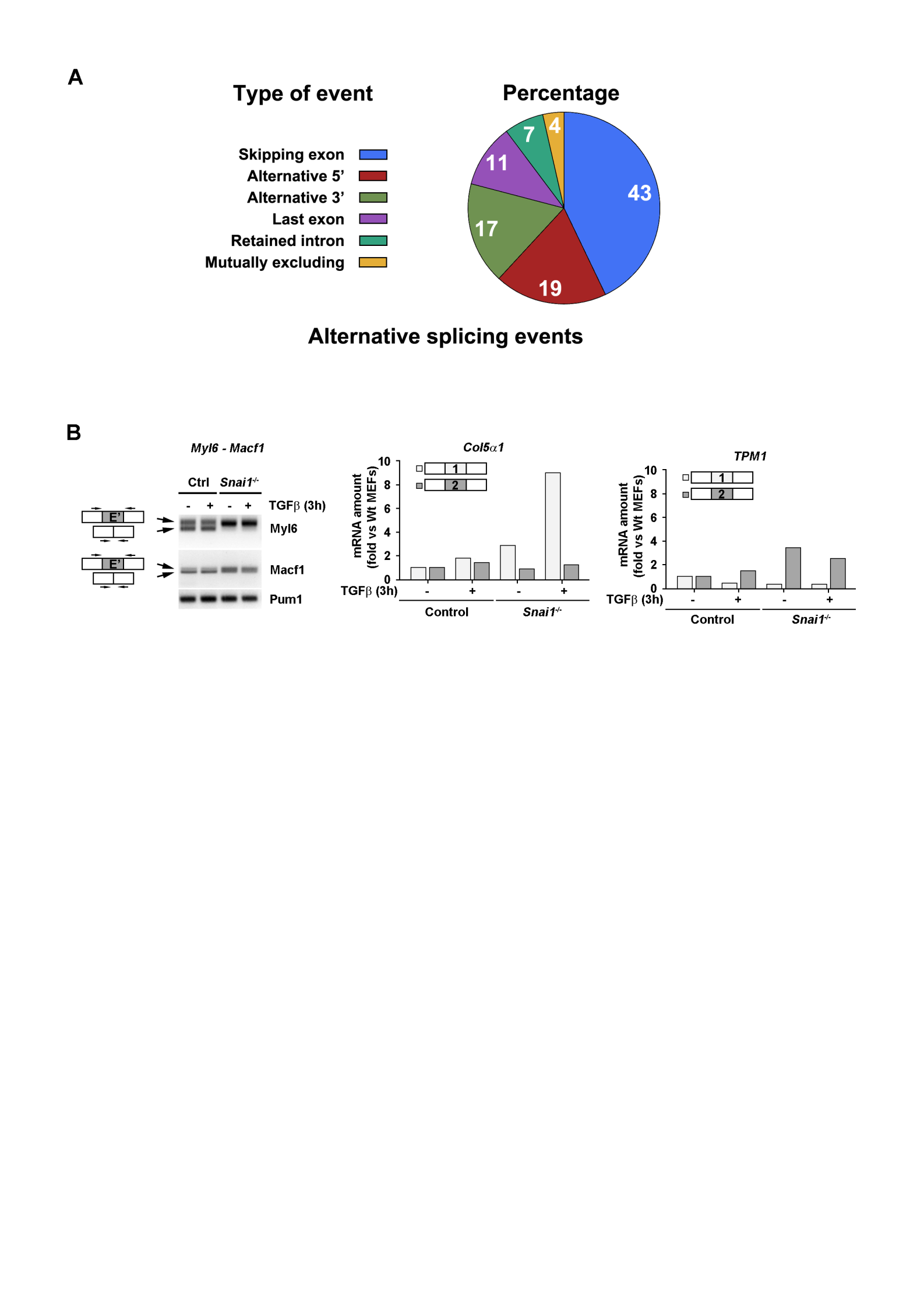
**

**Depletion of *Snail1^-/-^* modulates alternative splicing. A,** Type and percentage of alternative splicing events modulated by Snail1 depletion. Deep RNA sequencing from samples of Control and *Snai1^-/-^* MEFs treated with 5ng/mL TGFβ for 3 h was analyzed with the SUPPA2 pipeline to detect alternative splicing events. Supplementary Table S1 includes a list of the 674 differentially spliced events. The percentage of each splicing event type is shown in a pie chart. **B,** Relative RNA amount of *Myl6*, *Macf1,* *Col5α1* and *Tpm1* isoforms in Control and *Snai1^-/-^* MEFs. RNA obtained from indicated MEFs treated 3 h with 5ng/mL of TGFβ was retrotranscribed and amplified by sqPCR (*Myl6 and Macf1*) or qPCR (*Col5α1* and *Tpm1)* using specific primers. The resulting sqPCR DNA was visualized by electrophoresis on a 2% agarose gel. Quantitative PCR values for indicate genes were normalized with Pum1 values.

**Supplementary Figure S3**

**
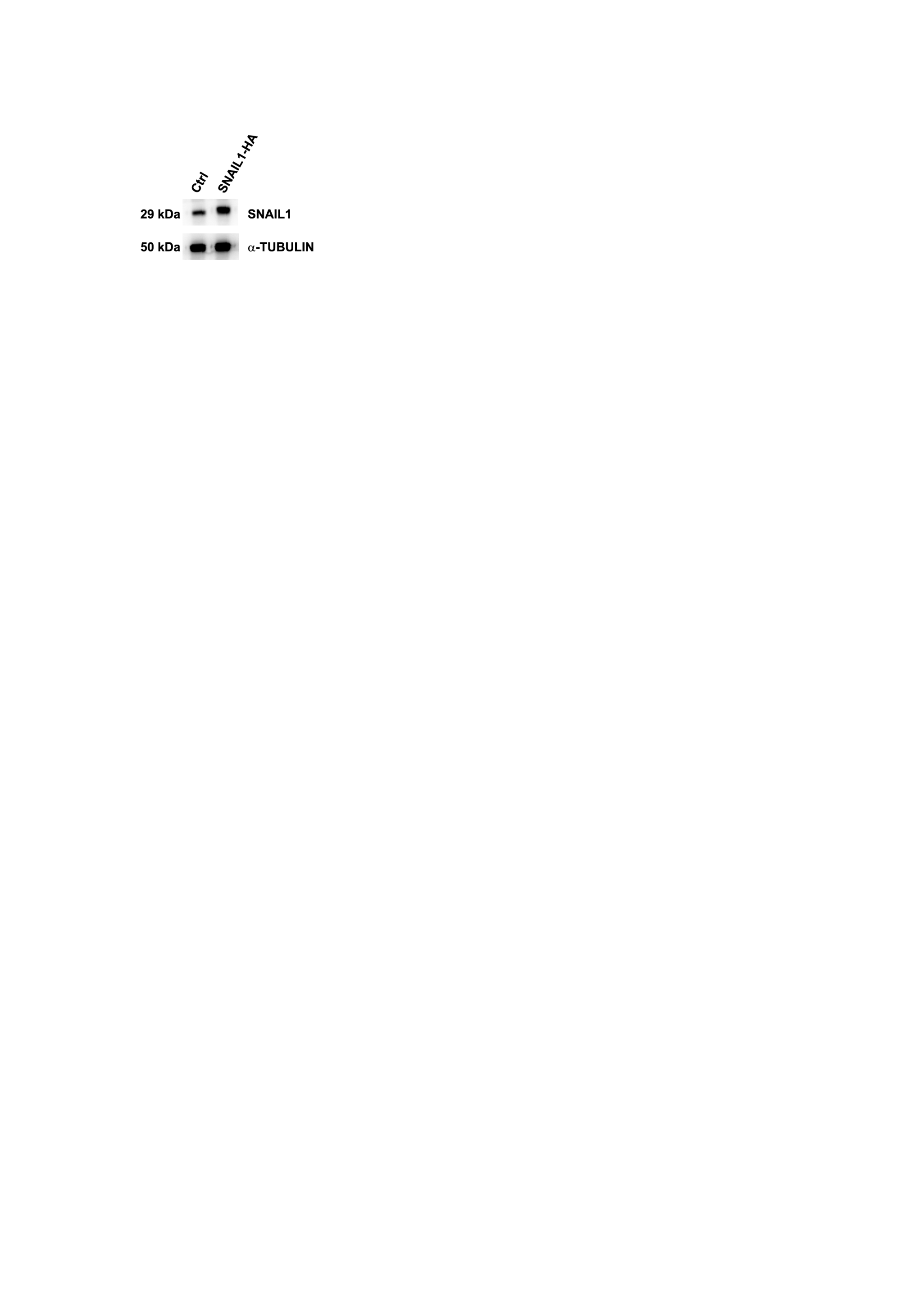
**

**Protein amount of SNAIL1 and α-TUBULIN in MEFs transfected with Snail-HA**. MEF control and MEF expressing exogenous SNAIL1-HA were lysed in SDS buffer and levels of the indicated proteins were analyzed by Western Blot.

**Supplementary Figure S4**

**
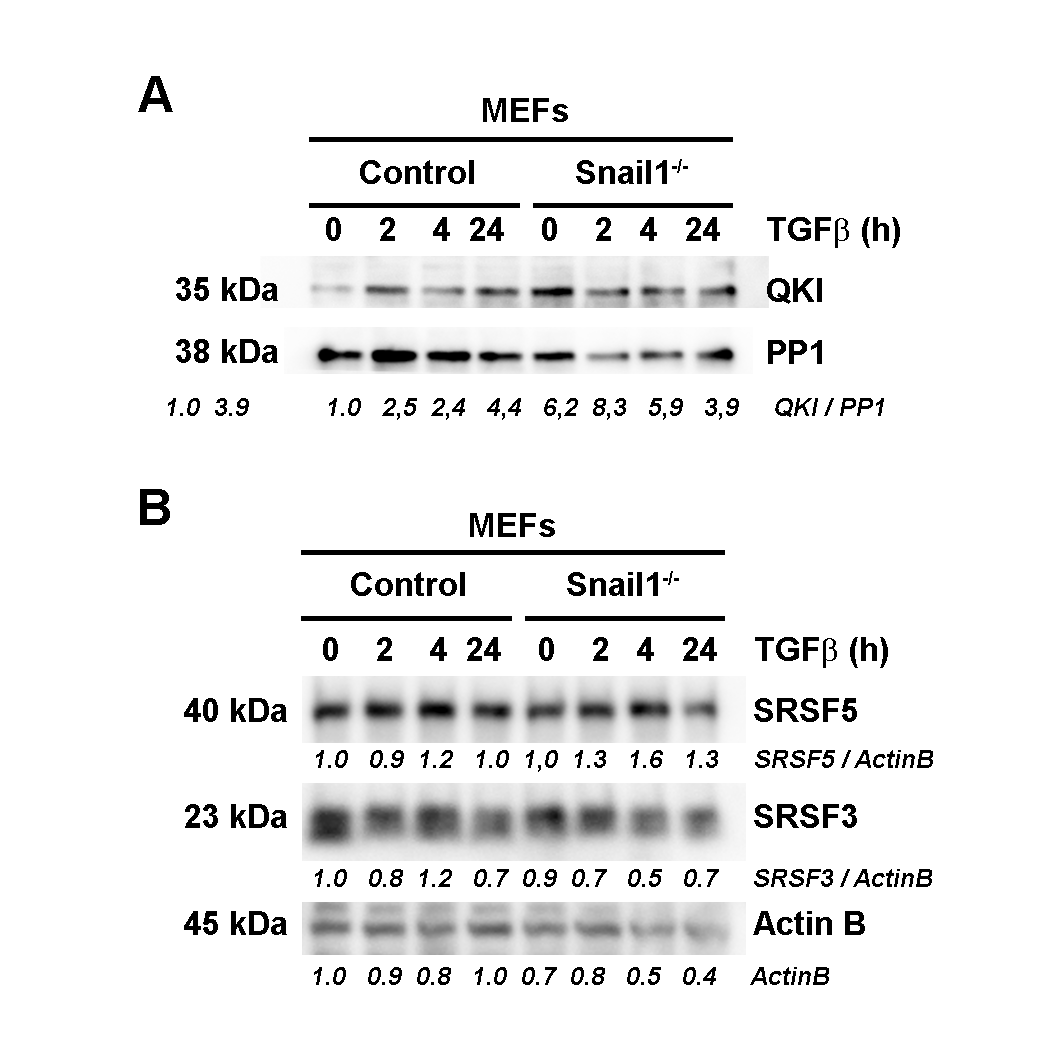
**

**Expression of splicing factors modulating EDA alternative splicing in Control and *Snai1^-/-^*** **MEFs. A,** Depletion of *Snai1* in MEFs increases QKI levels. B, Expression of SFRF3 and SRSF5 in Control and control and *Snai1^-/-^* MEFs. Indicated proteins from each cell line treated with 5 ng/mL TGFβ for indicated times were analyzed by Western blot. The intensity of the bands was calculated with an ImageJ gel plugin, and the splicing factor values were normalized with the Actin B or PP1 values. Italic numbers indicate the fold increase relative to control MEFs.

**Supplementary Figure S5**

**
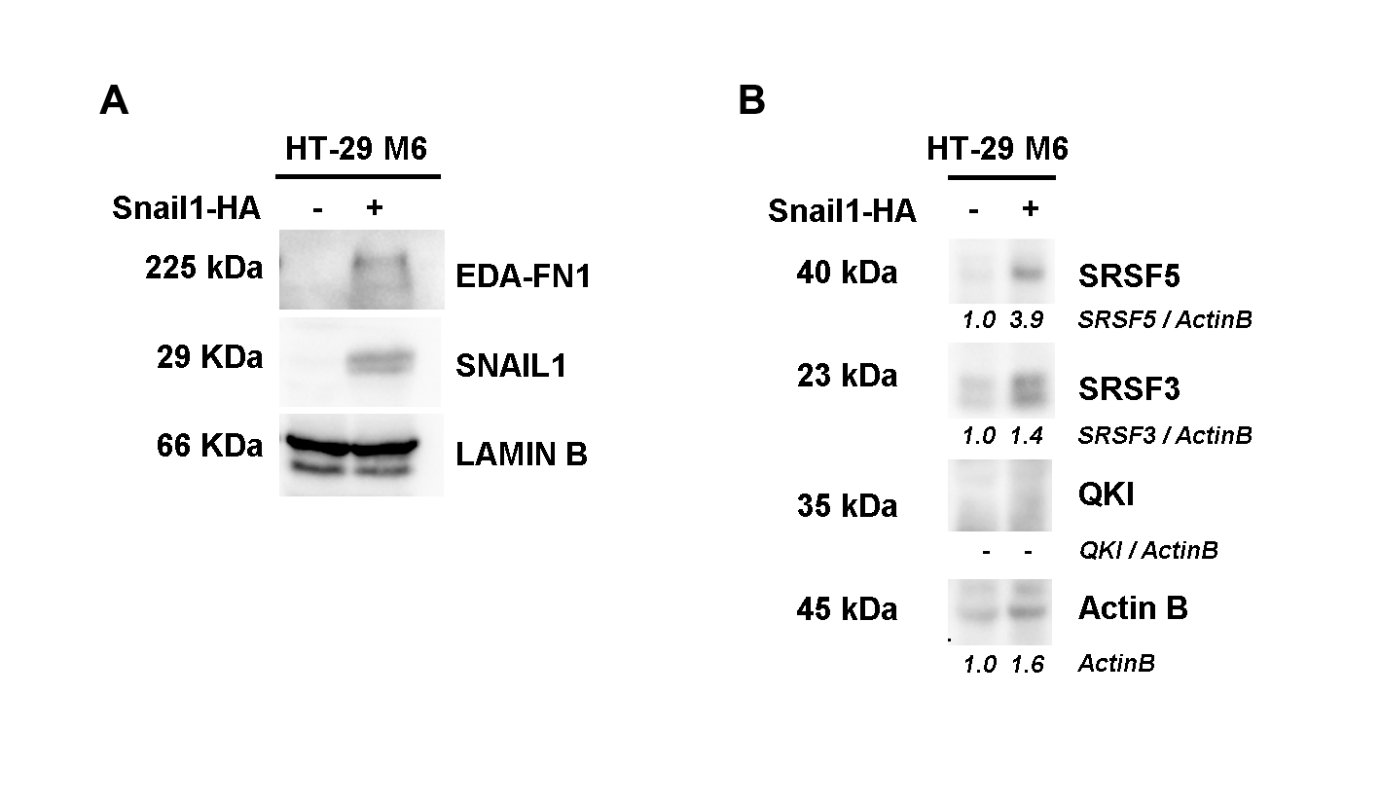
**

**Over-expression of SNAIL1 in epithelial cells increases fibronectin EDA and SRSF5 levels. A,** Expression of FN1-EDA in HT-29 M6 control and expressing exogenous SNAIL1-HA. B, Expression of SRSF3, SRSF5, and QKI in HT-29 M6 control and expressing exogenous SNAIL1-HA. Indicated proteins from each cell line were analyzed by Western blot. The intensity of the bands was calculated with an ImageJ gel plugin and the splicing factor values were normalized with the Actin B values. Italic numbers indicate the fold increase relative to control HT29 M6 cells.

**Supplementary Figure S6**

**
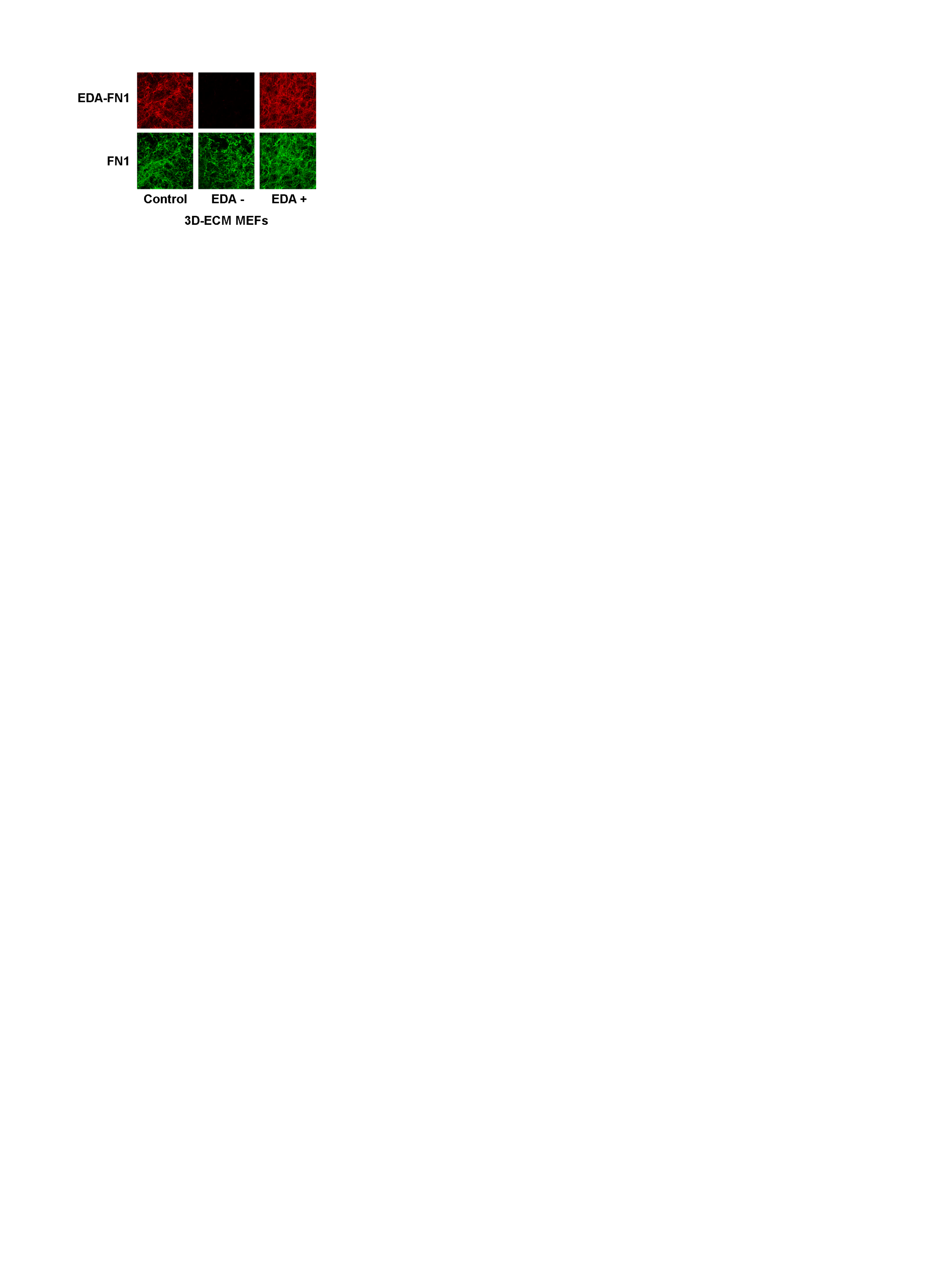
**

**Fibronectin EDA detection in 3D-ECMs.** MEFs were seeded on coverslips and allowed to produce extracellular matrix for 6 days. Cell cultures were then fixed with 4% PFA and analyzed by IF with anti-EDA Fibronectin (red) and anti-panFibronectin (green).

**Supplementary Figure S7**

**
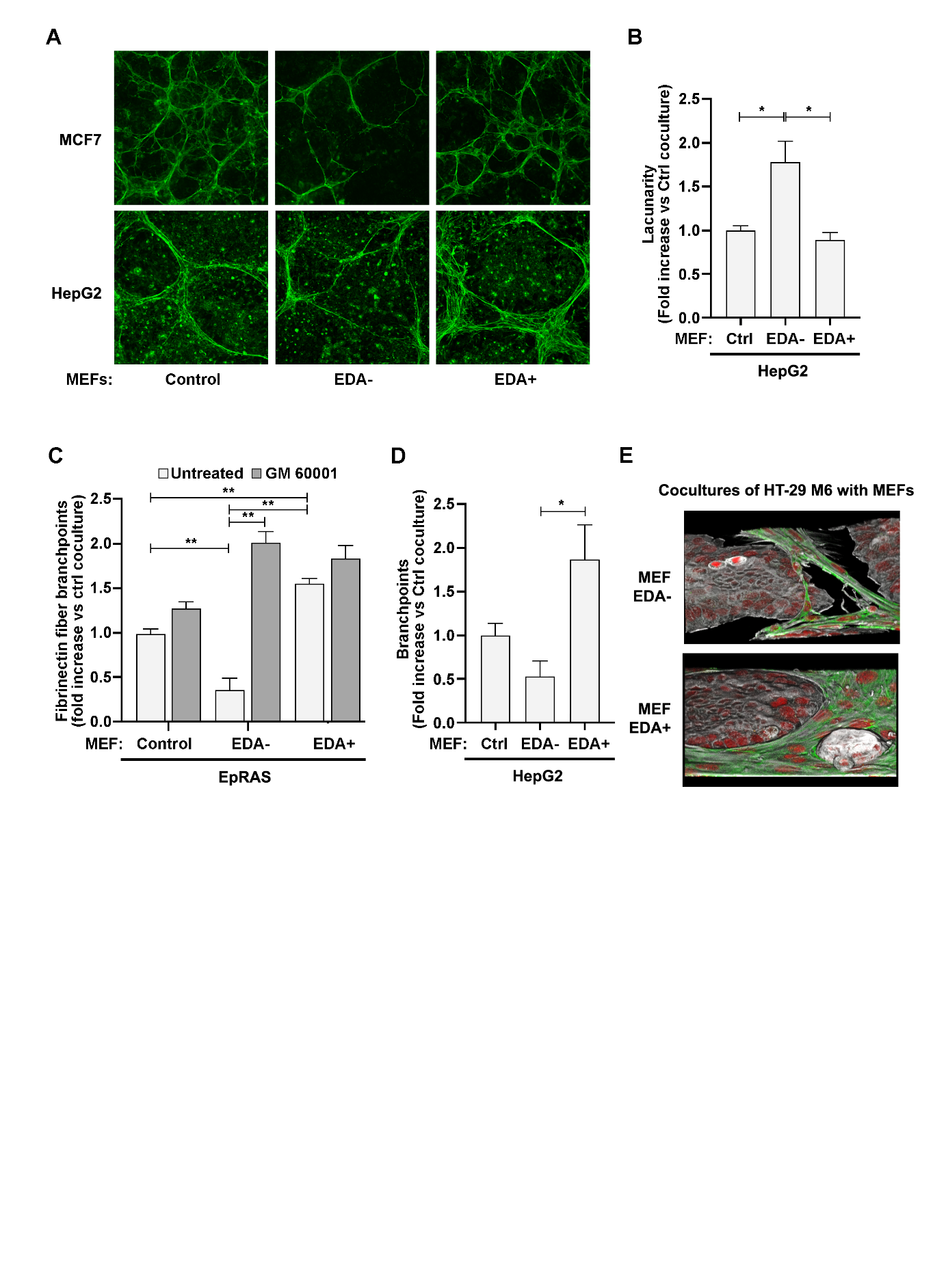
**

**Fibronectin fiber organization in cocultures of tumor cells and ibroblasts is EDA and metalloprotease dependent**. **A**, Fibronectin fiber organization in MCF7 or HepG2 cells cocultures with fibroblasts. Indicated tumor cells and MEFs were cocultured on glass coverslips for 3 days. Cocultures were analyzed by IF with anti-fibronectin (green). Microscopy images are shown. **B**, Fibronectin fiber lacunarity in HepG2 cocultures with fibroblasts is EDA dependent. Lacunary in fibronectin images obtained as in A was quantified using the TWOMBLI plugin of ImageJ software and fold increase with respect to values in control MEF cocultures was plotted. **C**, Fibronectin fiber branchpoints in EpRas cells cocultures with fibroblasts is EDA and metalloprotease dependent. Fiber branchpoinst in fibronectin images obtained as in Fig. 5A was quantified using the TWOMBLI plugin of ImageJ software and fold increase with respect to values in untreated control MEF cocultures was plotted. **D**, Fibronectin fiber branchpoints in HepG2 cocultures with fibroblasts is EDA dependent. Fiber branchpoints in fibronectin images obtained as in A was quantified using the TWOMBLI plugin of ImageJ software and fold increase with respect to values in control MEF cocultures was plotted. **E**, Tridimensional reconstruction of HT-29 M6 cocultured with MEFs. Z-stacks images were obtained with immunofluorescence confocal microscopy (630x) of cocultures from **Fig. 5C.** 3D rendering of Z-stacks with ImageJ volume viewer were obtained. Fibronectin (green), phalloidin (white) and DAPI (red) staining are shown.

**Supplementary Figure S8**

**
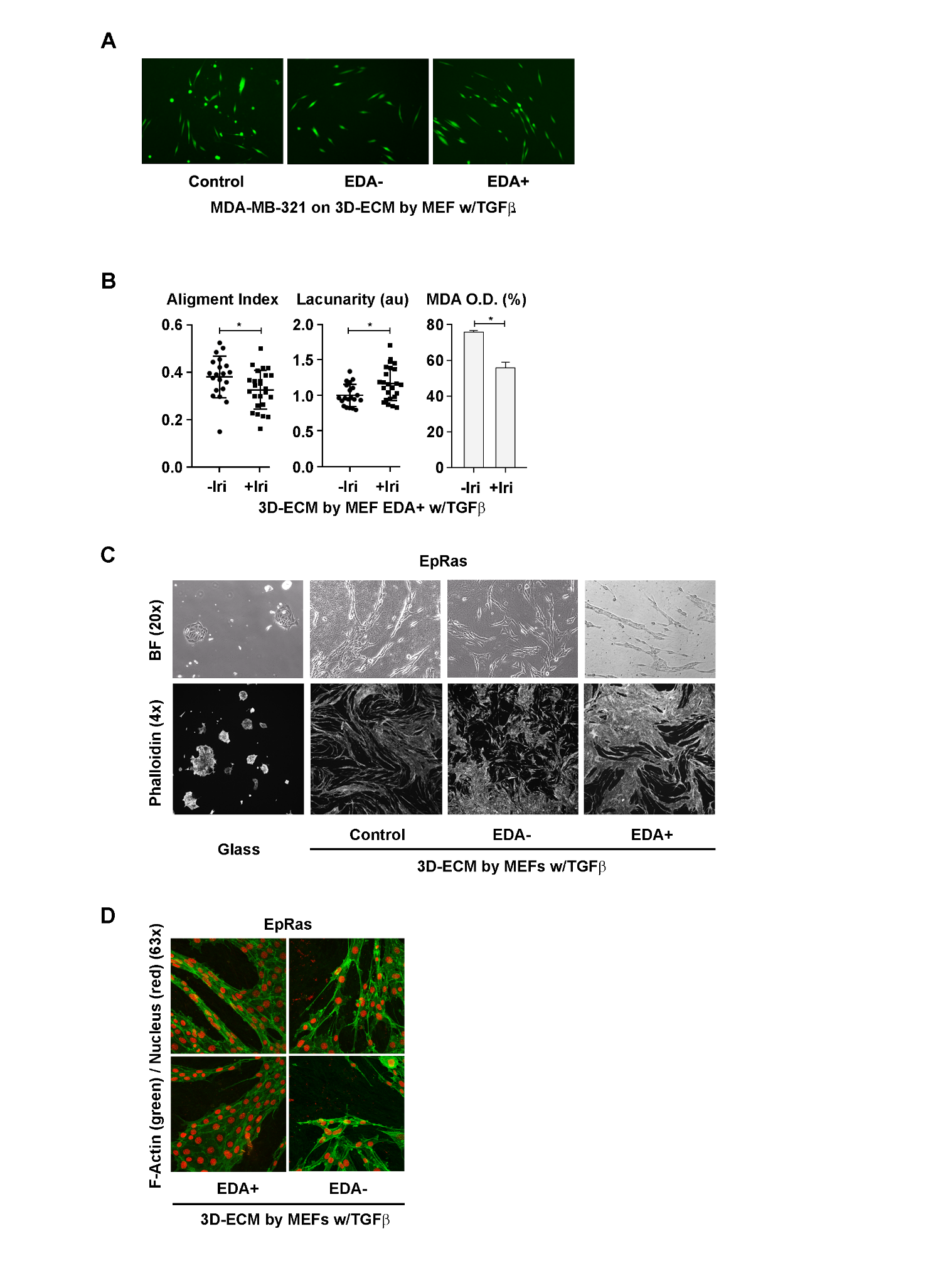
­**

**Aspect of tumor cells on Fibronectin EDA+ and - matrices. A**, Immunofluorescence of migrating MDA-MB-231 tumor cells. Cell tracker labeled MDA-MB-231 tumor cells were seeded on top of decellularized 3D-ECMs generated by indicated MEFs in the presence of 5ng/ml of TGFβ. Cells were recorded overnight by taking immunofluorescense images every 15 min with life microscopy. An image of MDA cells on each 3D-ECM is shown. **B**, Irigenin alters 3D-ECM formation leading to a decrease in the oriented migration of MDA-MB-231 tumor cells. 3D-ECMs were produced by the indicated MEF lines activated with 5ng/ml TGFβ in the absence (-Iri) or presence (+Iri) of 50 μM of Irigenin. ECMs were decellularized and either stained by immunofluorescence with an anti-fibronectin and analyzed by TWOMBLI (Alignment Index and Lacunarity plots) or used as a substrate for labeled MDA to be allowed to migrate. Migration was recorded overnight as in **A** and oriented movement was quantified as percentage of cell movements towards the same direction (up to 21˚ deviation from the mode). **C,** Collective organization of EpRas on 3D-ECMs. EpRAs cells were plated on glass or decellularized 3D-ECMs generated by the indicated MEFS in the presence of 5ng/mL TGFβ. At least 24 h later, cell migration was recorded overnight by time lapse microscopy. Representative bright field (BF) images taken during the recording are shown. Eventually, cells were fixed with 4% PFA and analyzed by IF with Phalloidin (gray). Low magnification images are shown to illustrate the aspect of the colonies. **D**, Colonies of EpRas cells on 3D-ECMs from TGFβ-treated EDA+ and EDA- MEFs. Two representative phalloidin immunofluorescence images obtained with the 63x confocal microscopy objective are shown. Phalloidin signal in green and DAPI in red.

**Supplementary Figure S9**

**^

^**

**Naïve fibroblasts are induced to assemble αSMA stress fibers by 3D-ECMs including EDA.** MSC or NIH-3T3 were grown 24h on decellularized 3D-matrices generated by EDA - or EDA + MEFs before labeling α-SMA and nuclei by immunofluorescence. Images obtained through fluorescence microscopy were used quantify the percentage of cells presenting α-SMA-positive stress fibers. **A**, Example of MSC non-presenting (negative) or presenting (positive) α-SMA-positive stress fibers. **B**, Representative images of indicated fibroblasts on matrices.

**Supplementary Figure S10**


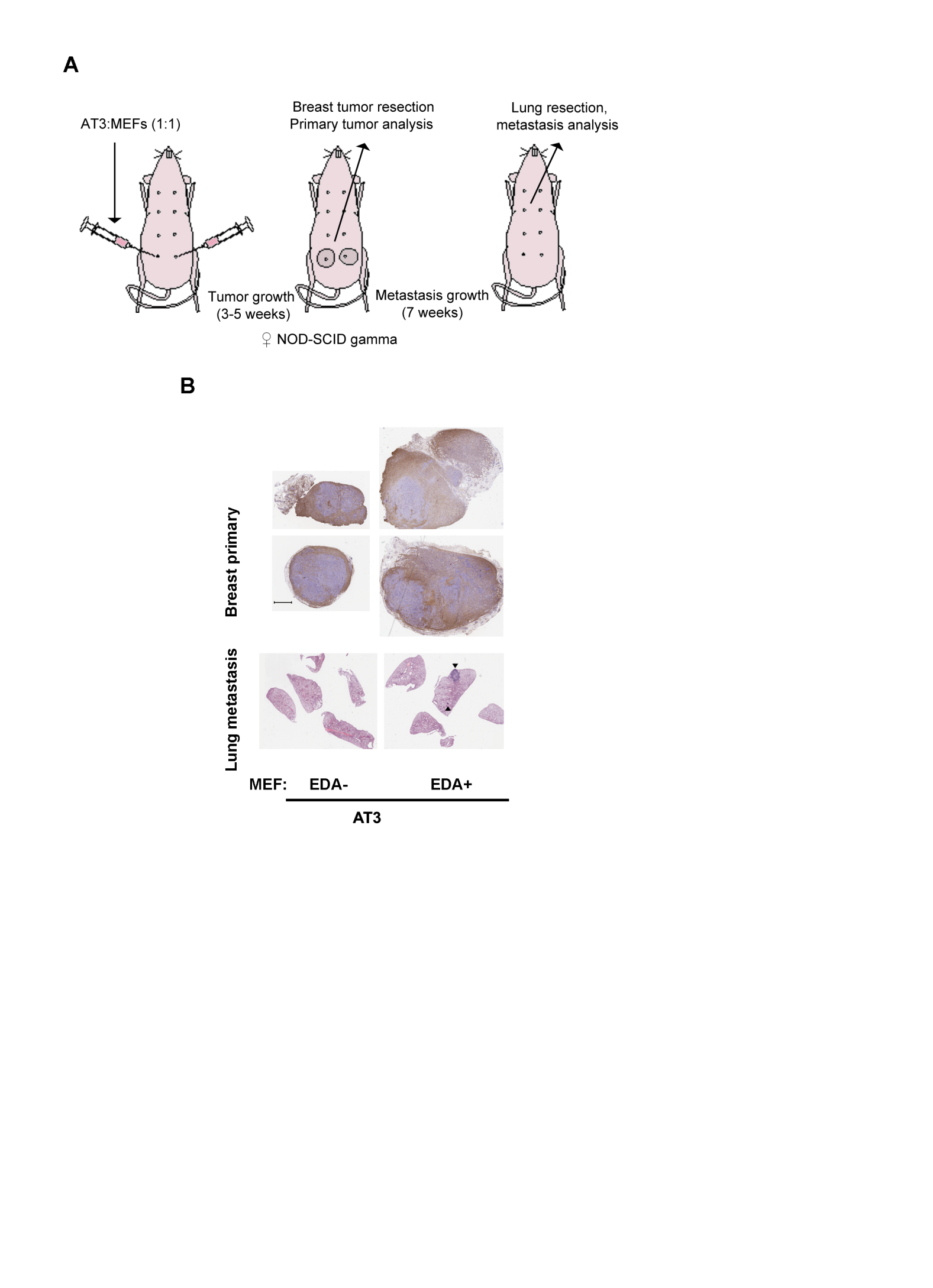


**Fibroblasts lacking EDA fibronectin prevent metastasis formation. A**, Experimental in vivo metastatic tumor model. 5x10^4^ AT-3 and either 5x10^4^ EDA- MEF or 5x10^4^ EDA+ MEF were co-injected orthotopically into the inguinal mammary fat pad of NOD-SCID gamma mice. Tumors were simultaneously resected when bigger tumors reached 0.5cm approximately. Extracted tumors were further measured, and immunohistochemistry was performed on 4% PFA-fixed paraffin-embedded tissues. Mice were kept for seven more weeks to allow metastasis formation in the lung and were humanely sacrificed. **B**, Histology of primary tumors and lung metastasis. Representative fibronectin hematoxylin staining of primary tumors and eosin hematoxylin staining of lung sections are shown. Black arrowheads point to lung metastasis.

**SUPPLEMENTARY IMAGES**

**Uncropped blot and gel images used to prepare the figures

**

**
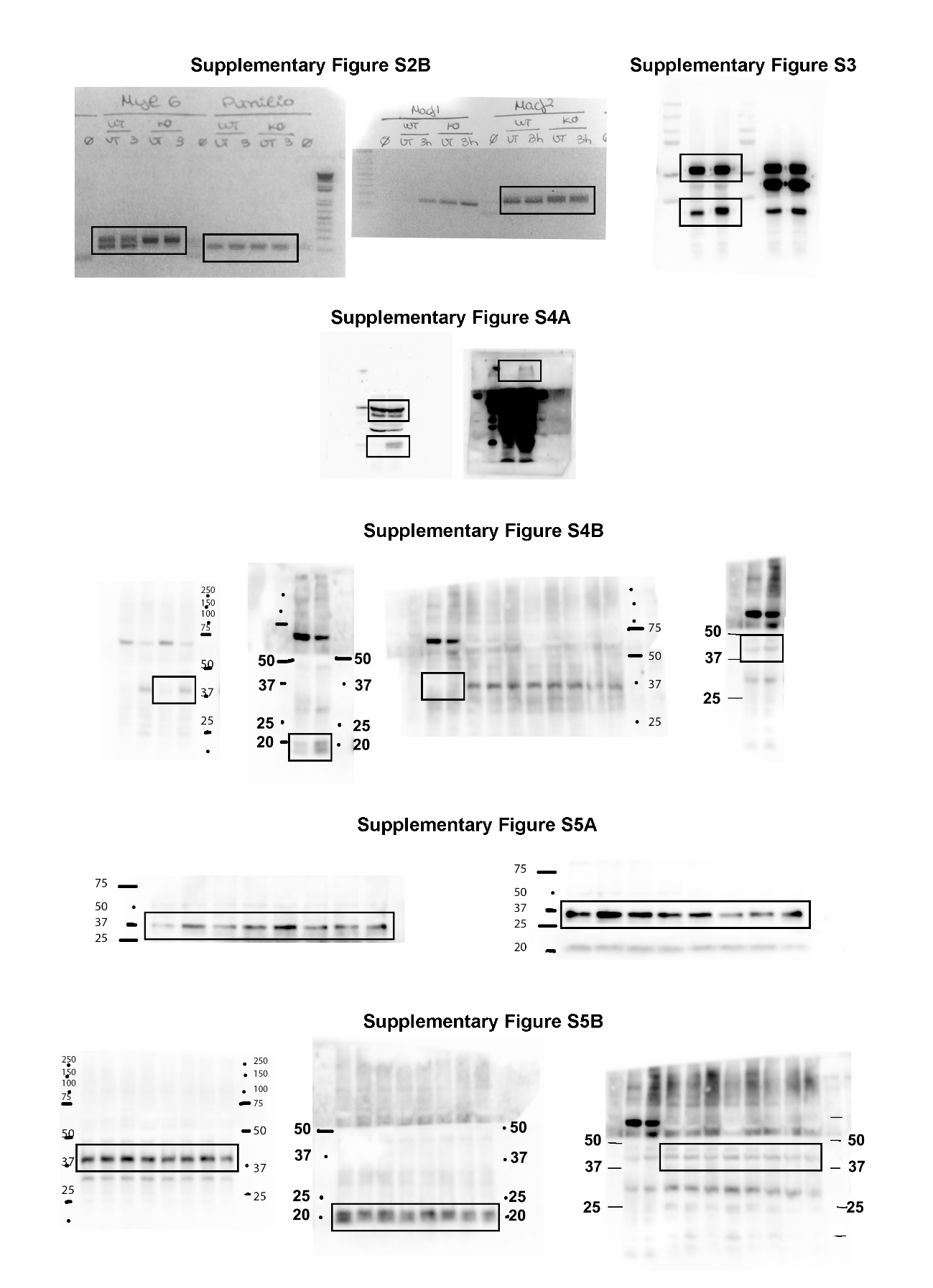
**

**SUPPLEMENTARY VIDEOS**

**Supplementary Video S1**

**EpRas cell moving on glass coverslips.** EpRas cells were plated on glass coverslips and 24 hlater were recorded overnight by taking images every 15 min with life microscopy.

**Supplementary Video S2**

**EpRas cell moving on EDA- 3DECMs.** EpRas cells were plated on decellularized 3D-ECM derived from MEFs EDA- treated with 5ng/mL TGFβ and 24 ho later were recorded overnight by taking images every 15 min with life microscopy.

**Supplementary Video S3**

**EpRas cell moving on EDA+ 3DECMs.** EpRas cells were plated on decellularized 3D-ECD derived from MEFs EDA+ treated with 5ng/mL TGFβ, and 24 h later were recorded overnight by taking images every 15 min with life microscopy.

**SUPPLEMENTARY TABLES**

**Supplementary Table S1**

**List of 674 splicing events significantly changed between control and *Snai1^-/-^***. RNA samples from Control and *Snai1^-/-^* MEFs treated with 5ng/mL TGFβ for 3 h were paired-end sequenced in CRG Sequencing Unit until reaching 80M reads/sample. The SUPPA2 software was run with a threshold of 0.1 ΔPSI (https://github.com/comprna/SUPPA).

| **Gene name** | **Type** | **dPSI KO/WT** | **p-val KO/WT** | **rownames(significant)** | |
| --- | --- | --- | --- | --- | --- |
| Srsf6 | SE | 0.2438135877 | 0 | ENSMUSG00000016921;SE:2:162932142-162932815:162933082-162933426:+ |  |
| Rgs17 | SE | 0.2389453094 | 0 | ENSMUSG00000019775;SE:10:5862662-5918184:5918253-5922228:- |  |
| Eif4a2 | SE | 0.2384287659 | 0 | ENSMUSG00000022884;SE:16:23111896-23112351:23112457-23113167:+ |  |
| Tmem80 | SE | -0.3091991075 | 0 | ENSMUSG00000025505;SE:7:141328266-141331418:141331561-141332910:+ |  |
| Fn1 | SE | 0.3417913771 | 0 | ENSMUSG00000026193;SE:1:71602497-71603660:71603929-71604970:- |  |
| Eif4e2 | SE | 0.1865904309 | 0 | ENSMUSG00000026254;SE:1:87214019-87214346:87214495-87219581:+ |  |
| Fip1l1 | SE | -0.1807794927 | 0 | ENSMUSG00000029227;SE:5:74572585-74585046:74585072-74587003:+ |  |
| Sh3glb1 | SE | 0.214139385 | 0 | ENSMUSG00000037062;SE:3:144697467-144701760:144701846-144705513:- |  |
| Ipo9 | SE | 0.2162953969 | 0 | ENSMUSG00000041879;SE:1:135420353-135426931:135427041-135430268:- |  |
| Pitpnb | SE | 0.2115920878 | 0 | ENSMUSG00000050017;SE:5:111383110-111385520:111385604-111386503:+ |  |
| Pdp1 | SE | 0.2761212021 | 0.000999001 | ENSMUSG00000049225;SE:4:11962233-11965614:11965648-11966302:- |  |
| Acly | SE | 0.1802095833 | 0.0014985015 | ENSMUSG00000020917;SE:11:100498762-100503160:100503189-100504203:- |  |
| Cuedc2 | SE | 0.1888428306 | 0.0014985015 | ENSMUSG00000036748;SE:19:46332701-46338313:46338420-46338569:- |  |
| Pigk | SE | -0.1739519747 | 0.0014985015 | ENSMUSG00000039047;SE:3:152738321-152740129:152740240-152742489:+ |  |
| Pigk | SE | 0.3715013354 | 0.0014985015 | ENSMUSG00000039047;SE:3:152740240-152740753:152740893-152742489:+ |  |
| Slk | SE | 0.2115022206 | 0.001998002 | ENSMUSG00000025060;SE:19:47625484-47627648:47627740-47635469:+ |  |
| Ndc1 | SE | -0.1894243141 | 0.001998002 | ENSMUSG00000028614;SE:4:107368148-107368757:107368877-107371262:+ |  |
| Hsbp1 | SE | 0.1595775703 | 0.001998002 | ENSMUSG00000031839;SE:8:119345733-119346825:119346867-119348036:+ |  |
| Aplp2 | SE | 0.1843689006 | 0.001998002 | ENSMUSG00000031996;SE:9:31164673-31167561:31167728-31168708:- |  |
| Pmepa1 | SE | 0.146341973 | 0.001998002 | ENSMUSG00000038400;SE:2:173234461-173271567:173271680-173276088:- |  |
| Ascc2 | SE | 0.199058357 | 0.0024975025 | ENSMUSG00000020412;SE:11:4672497-4673328:4673447-4679252:+ |  |
| Sntb2 | SE | -0.2356856825 | 0.0024975025 | ENSMUSG00000041308;SE:8:107001706-107009781:107009965-107011292:+ |  |
| Hars2 | SE | 0.3874961712 | 0.002997003 | ENSMUSG00000019143;SE:18:36788670-36789157:36789284-36790134:+ |  |
| Pam | SE | -0.2316632773 | 0.002997003 | ENSMUSG00000026335;SE:1:97853232-97864203:97864520-97884126:- |  |
| Abhd5 | SE | 0.224779973 | 0.002997003 | ENSMUSG00000032540;SE:9:122351839-122363815:122363864-122365136:+ |  |
| Prrx1 | SE | 0.166544381 | 0.0034965035 | ENSMUSG00000026586;SE:1:163248396-163253977:163254048-163257761:- |  |
| Fip1l1 | SE | -0.1658168016 | 0.0034965035 | ENSMUSG00000029227;SE:5:74557149-74564519:74564626-74571099:+ |  |
| Ap1g1 | SE | -0.1582524107 | 0.003996004 | ENSMUSG00000031731;SE:8:109829675-109830613:109830621-109832717:+ |  |
| Lss | SE | -0.1969407817 | 0.003996004 | ENSMUSG00000033105;SE:10:76536365-76537569:76537663-76538352:+ |  |
| Bri3bp | SE | 0.2096055944 | 0.003996004 | ENSMUSG00000037905;SE:5:125451772-125452148:125452245-125454326:+ |  |
| Cd47 | SE | -0.1504961968 | 0.003996004 | ENSMUSG00000055447;SE:16:49868112-49869018:49869080-49884165:+ |  |
| Anxa6 | SE | -0.1492323306 | 0.0044955045 | ENSMUSG00000018340;SE:11:54986215-54987051:54987068-54991391:- |  |
| Enpp2 | SE | -0.2792624815 | 0.0044955045 | ENSMUSG00000022425;SE:15:54875760-54877776:54877931-54882130:- |  |
| Hnrnpdl | SE | 0.1208225236 | 0.0044955045 | ENSMUSG00000029328;SE:5:100034984-100035635:100035739-100036104:- |  |
| Abi3bp | SE | 0.1044550812 | 0.0044955045 | ENSMUSG00000035258;SE:16:56647099-56650711:56650770-56652137:+ |  |
| Abi3bp | SE | -0.1324031191 | 0.0044955045 | ENSMUSG00000035258;SE:16:56650770-56651307:56651354-56652137:+ |  |
| Ube2j1 | SE | -0.1330016361 | 0.0046620047 | ENSMUSG00000028277;SE:4:33031616-33036709:33036782-33038199:+ |  |
| Rab11a | SE | -0.5807872072 | 0.004995005 | ENSMUSG00000004771;SE:9:64716988-64725453:64725582-64726624:- |  |
| Helz | SE | -0.2177445401 | 0.004995005 | ENSMUSG00000020721;SE:11:107549401-107555519:107555575-107574938:+ |  |
| Nmt1 | SE | -0.1359540577 | 0.004995005 | ENSMUSG00000020936;SE:11:103028702-103043168:103043276-103046379:+ |  |
| Tmub2 | SE | -0.3005547396 | 0.004995005 | ENSMUSG00000034757;SE:11:102285088-102285980:102286116-102287308:+ |  |
| Sh3kbp1 | SE | 0.5363324903 | 0.004995005 | ENSMUSG00000040990;SE:X:159840611-159841076:159841186-159865325:+ |  |
| Mkrn2 | SE | -0.2499387684 | 0.005994006 | ENSMUSG00000000439;SE:6:115610700-115611635:115611939-115613327:+ |  |
| Eif2s1 | SE | -0.1190059817 | 0.005994006 | ENSMUSG00000021116;SE:12:78862180-78866526:78866767-78874564:+ |  |
| Rtn3 | SE | 0.1652992419 | 0.005994006 | ENSMUSG00000024758;SE:19:7435138-7467811:7467867-7482939:- |  |
| Dicer1 | SE | 0.2101380136 | 0.005994006 | ENSMUSG00000041415;SE:12:104714906-104720617:104720718-104721971:- |  |
| Guf1 | SE | 0.2578436602 | 0.0062437562 | ENSMUSG00000029208;SE:5:69561857-69563083:69563222-69564524:+ |  |
| Guf1 | SE | -0.4736813226 | 0.0062437562 | ENSMUSG00000029208;SE:5:69561857-69564524:69564647-69565430:+ |  |
| Srpk1 | SE | -0.1448967853 | 0.0064935065 | ENSMUSG00000004865;SE:17:28599869-28600243:28600456-28600869:- |  |
| Phf10 | SE | 0.1032841863 | 0.0064935065 | ENSMUSG00000023883;SE:17:14952773-14954015:14954124-14954792:- |  |
| Fbxo28 | SE | -0.2175930179 | 0.0067432567 | ENSMUSG00000047539;SE:1:182318005-182326254:182326392-182329874:- |  |
| Rchy1 | SE | 0.1127591445 | 0.006993007 | ENSMUSG00000029397;SE:5:91957650-91957878:91957997-91962630:- |  |
| Zc2hc1a | SE | -0.1701501555 | 0.006993007 | ENSMUSG00000043542;SE:3:7528684-7539076:7539183-7551383:+ |  |
| Ergic1 | SE | 0.1333978381 | 0.0074925075 | ENSMUSG00000001576;SE:17:26561632-26605464:26605584-26608593:+ |  |
| Sptlc2 | SE | -0.1281458458 | 0.0074925075 | ENSMUSG00000021036;SE:12:87346801-87350261:87350385-87355526:- |  |
| Ybx3 | SE | -0.1050176728 | 0.0074925075 | ENSMUSG00000030189;SE:6:131370414-131375974:131376180-131379358:- |  |
| Tsc22d2 | SE | -0.13396498 | 0.007992008 | ENSMUSG00000027806;SE:3:58417613-58428228:58428299-58459331:+ |  |
| Ankrd11 | SE | 0.1303788977 | 0.007992008 | ENSMUSG00000035569;SE:8:122900149-122900602:122900698-122908668:- |  |
| Mxd4 | SE | -0.1310153255 | 0.007992008 | ENSMUSG00000037235;SE:5:34178924-34184314:34184343-34187329:- |  |
| Fn1 | SE | 0.29548683 | 0.0082417582 | ENSMUSG00000026193;SE:1:71597387-71597642:71597747-71598374:- |  |
| Idh3a | SE | 0.109169206 | 0.0084915085 | ENSMUSG00000032279;SE:9:54589920-54592161:54592255-54592394:+ |  |
| Dusp11 | SE | -0.1125525741 | 0.008991009 | ENSMUSG00000030002;SE:6:85950093-85950514:85950560-85952305:- |  |
| Flot2 | SE | 0.267292247 | 0.008991009 | ENSMUSG00000061981;SE:11:78053453-78054785:78054875-78055757:+ |  |
| Hypk | SE | -0.2344105119 | 0.0093240093 | ENSMUSG00000027245;SE:2:121457291-121457646:121457701-121457992:+ |  |
| Spata7 | SE | 0.2507105715 | 0.0094905095 | ENSMUSG00000021007;SE:12:98632037-98634224:98634319-98637566:+ |  |
| Rftn2 | SE | 0.1912244098 | 0.0094905095 | ENSMUSG00000025978;SE:1:55195637-55201345:55201422-55204183:- |  |
| Mark1 | SE | 0.1288904982 | 0.0094905095 | ENSMUSG00000026620;SE:1:184945000-184984847:184984980-184999422:- |  |
| Inip | SE | -0.1577711825 | 0.0094905095 | ENSMUSG00000038544;SE:4:59775549-59782254:59782334-59783728:- |  |
| Zranb2 | SE | -0.2606008442 | 0.00999001 | ENSMUSG00000028180;SE:3:157536386-157536448:157536586-157537587:+ |  |
| Ptp4a2 | SE | -0.1729376252 | 0.00999001 | ENSMUSG00000028788;SE:4:129842762-129845062:129845192-129846465:+ |  |
| Limch1 | SE | -0.3540991436 | 0.00999001 | ENSMUSG00000037736;SE:5:66974662-66993143:66993178-66997120:+ |  |
| Srsf1 | SE | 0.463553232 | 0.0104895105 | ENSMUSG00000018379;SE:11:88049215-88049412:88049598-88049977:+ |  |
| Pcgf2 | SE | 0.1767457872 | 0.0104895105 | ENSMUSG00000018537;SE:11:97693568-97699206:97699300-97699536:- |  |
| Mcmbp | SE | -0.1083049091 | 0.0104895105 | ENSMUSG00000048170;SE:7:128698570-128699319:128699407-128702243:- |  |
| Tead1 | SE | 0.1738163136 | 0.010989011 | ENSMUSG00000055320;SE:7:112839480-112841888:112841950-112842040:+ |  |
| Tead1 | SE | 0.1664447165 | 0.010989011 | ENSMUSG00000055320;SE:7:112842102-112856053:112856064-112856798:+ |  |
| Rbm39 | SE | 0.2722416718 | 0.0110603682 | ENSMUSG00000027620;SE:2:156177906-156178880:156178952-156179177:- |  |
| Endov | SE | -0.3065993324 | 0.0114171543 | ENSMUSG00000039850;SE:11:119491459-119491785:119491956-119499527:+ |  |
| Rbbp6 | SE | -0.1391377644 | 0.0114885115 | ENSMUSG00000030779;SE:7:122997305-122997596:122997697-122998525:+ |  |
| Irak1bp1 | SE | -0.1761422804 | 0.0114885115 | ENSMUSG00000032251;SE:9:82830339-82837418:82837483-82846299:+ |  |
| Slain2 | SE | -0.1079754412 | 0.0114885115 | ENSMUSG00000036087;SE:5:72958286-72965804:72965881-72974531:+ |  |
| Sap30bp | SE | -0.1399099765 | 0.011988012 | ENSMUSG00000020755;SE:11:115960591-115961356:115961416-115961739:+ |  |
| Zdhhc20 | SE | 0.1670119389 | 0.011988012 | ENSMUSG00000021969;SE:14:57847062-57850145:57850180-57856610:- |  |
| Gnb4 | SE | -0.1739994701 | 0.011988012 | ENSMUSG00000027669;SE:3:32602140-32614100:32614346-32616439:- |  |
| Zranb2 | SE | 0.1603518529 | 0.011988012 | ENSMUSG00000028180;SE:3:157536386-157536483:157536586-157537587:+ |  |
| Mre11a | SE | 0.2002276857 | 0.011988012 | ENSMUSG00000031928;SE:9:14805527-14809804:14809884-14811830:+ |  |
| Adnp | SE | 0.1393015955 | 0.011988012 | ENSMUSG00000051149;SE:2:168189394-168204600:168204774-168206574:- |  |
| Ntan1 | SE | 0.142456492 | 0.0122377622 | ENSMUSG00000022681;SE:16:13819436-13827060:13827125-13827388:+ |  |
| Ntan1 | SE | 0.1425633149 | 0.0122377622 | ENSMUSG00000022681;SE:16:13832434-13834681:13834794-13835135:+ |  |
| Pmpca | SE | -0.1133277893 | 0.012987013 | ENSMUSG00000026926;SE:2:26390577-26391084:26391178-26391866:+ |  |
| Unc13a | SE | -0.5589970168 | 0.012987013 | ENSMUSG00000034799;SE:8:71666078-71666641:71666670-71671626:- |  |
| Bclaf1 | SE | -0.1038709401 | 0.012987013 | ENSMUSG00000037608;SE:10:20333572-20334499:20334645-20339709:+ |  |
| Psmg4 | SE | 0.1166737425 | 0.012987013 | ENSMUSG00000071451;SE:13:34163249-34166061:34166136-34177981:+ |  |
| Sar1a | SE | 0.134102014 | 0.0134865135 | ENSMUSG00000020088;SE:10:61680413-61684234:61684331-61684880:+ |  |
| Mecp2 | SE | -0.2465632892 | 0.0134865135 | ENSMUSG00000031393;SE:X:74037332-74079909:74080032-74085510:- |  |
| 1700025G04Rik | SE | 0.4438910491 | 0.0134865135 | ENSMUSG00000032666;SE:1:151921123-151974352:151974488-151980426:- |  |
| Picalm | SE | 0.1972860558 | 0.0134865135 | ENSMUSG00000039361;SE:7:90177603-90182219:90182368-90189150:+ |  |
| Myl6 | SE | -0.2927686398 | 0.0134865135 | ENSMUSG00000090841;SE:10:128491033-128491720:128491764-128492059:- |  |
| Map3k7 | SE | 0.1880278234 | 0.013986014 | ENSMUSG00000028284;SE:4:31992516-31994874:31994954-32002089:+ |  |
| Rbm34 | SE | -0.1038607548 | 0.013986014 | ENSMUSG00000033931;SE:8:126959585-126961912:126961971-126965382:- |  |
| Preb | SE | 0.4756344567 | 0.013986014 | ENSMUSG00000045302;SE:5:30955737-30955930:30956089-30957888:- |  |
| Ubr4 | SE | -0.1663587272 | 0.013986014 | ENSMUSG00000066036;SE:4:139431761-139432482:139432514-139433620:+ |  |
| Ubr4 | SE | 0.1663587272 | 0.013986014 | ENSMUSG00000066036;SE:4:139440822-139441192:139441296-139442686:+ |  |
| Pisd-ps1 | SE | -0.2746093952 | 0.013986014 | ENSMUSG00000082286;SE:11:3126058-3127131:3127219-3127479:+ |  |
| Fam210a | SE | 0.134020815 | 0.0144855145 | ENSMUSG00000038121;SE:18:68276274-68279267:68279428-68300074:- |  |
| Rock2 | SE | 0.1060939101 | 0.014985015 | ENSMUSG00000020580;SE:12:16973149-16973440:16973610-16974842:+ |  |
| Smc6 | SE | -0.3695789628 | 0.014985015 | ENSMUSG00000020608;SE:12:11271871-11273994:11274111-11279605:+ |  |
| Clmn | SE | 0.1650872012 | 0.014985015 | ENSMUSG00000021097;SE:12:104774549-104775848:104775928-104776945:- |  |
| Itsn1 | SE | -0.6880109207 | 0.014985015 | ENSMUSG00000022957;SE:16:91854655-91861194:91861239-91863257:+ |  |
| Spopl | SE | 0.1869277091 | 0.014985015 | ENSMUSG00000026771;SE:2:23537638-23541519:23541615-23543197:- |  |
| Ppp3ca | SE | 0.1055195749 | 0.014985015 | ENSMUSG00000028161;SE:3:136928619-136932011:136932040-136935029:+ |  |
| Tra2a | SE | -0.1118845668 | 0.014985015 | ENSMUSG00000029817;SE:6:49252515-49252803:49253108-49263838:- |  |
| Tial1 | SE | -0.1135976603 | 0.014985015 | ENSMUSG00000030846;SE:7:128446270-128446731:128446798-128448121:- |  |
| Ogt | SE | 0.1194848627 | 0.014985015 | ENSMUSG00000034160;SE:X:101661331-101663359:101663554-101663843:+ |  |
| Stx17 | SE | 0.2467368783 | 0.014985015 | ENSMUSG00000061455;SE:4:48124958-48129053:48129098-48131765:+ |  |
| Dab2 | SE | -0.2191174558 | 0.0153180153 | ENSMUSG00000022150;SE:15:6424697-6429296:6429949-6430954:+ |  |
| Bbs7 | SE | 0.1480262974 | 0.0154845155 | ENSMUSG00000037325;SE:3:36610628-36612012:36612077-36613250:- |  |
| Rgl1 | SE | -0.3992129365 | 0.015984016 | ENSMUSG00000026482;SE:1:152586615-152623818:152623928-152674860:- |  |
| Akap13 | SE | -0.2575351433 | 0.015984016 | ENSMUSG00000066406;SE:7:75679873-75683394:75683459-75685117:+ |  |
| Kcnk2 | SE | -0.4403139864 | 0.0164835165 | ENSMUSG00000037624;SE:1:189258898-189295594:189295711-189339785:- |  |
| Nrm | SE | 0.145638193 | 0.0164835165 | ENSMUSG00000059791;SE:17:35861517-35863439:35863635-35864112:+ |  |
| Gpatch2l | SE | -0.1211927116 | 0.016983017 | ENSMUSG00000021254;SE:12:86269061-86281449:86281543-86288800:+ |  |
| Commd7 | SE | 0.1230077812 | 0.016983017 | ENSMUSG00000056941;SE:2:153629246-153632188:153632262-153632594:- |  |
| Zfp950 | SE | -0.2734192074 | 0.016983017 | ENSMUSG00000074733;SE:19:61117288-61118227:61118350-61127007:- |  |
| Cpsf6 | SE | 0.1409685161 | 0.0174825175 | ENSMUSG00000055531;SE:10:117361360-117361774:117361884-117362014:- |  |
| Phactr4 | SE | -0.1199958025 | 0.0174825175 | ENSMUSG00000066043;SE:4:132378563-132383282:132383362-132386892:- |  |
| Ubqln1 | SE | 0.118744707 | 0.017982018 | ENSMUSG00000005312;SE:13:58179668-58180822:58180905-58183182:- |  |
| Slc35b4 | SE | 0.190887728 | 0.017982018 | ENSMUSG00000018999;SE:6:34170574-34172628:34172755-34176778:- |  |
| Uba3 | SE | 0.114674607 | 0.017982018 | ENSMUSG00000030061;SE:6:97203594-97205306:97205347-97205533:- |  |
| Kras | SE | 0.1151082677 | 0.017982018 | ENSMUSG00000030265;SE:6:145220724-145225074:145225197-145232095:- |  |
| Atxn1 | SE | -0.2227335099 | 0.017982018 | ENSMUSG00000046876;SE:13:45852456-45956455:45956575-45964815:- |  |
| Tyw3 | SE | -0.3429769976 | 0.017982018 | ENSMUSG00000047583;SE:3:154587610-154591084:154591155-154593717:- |  |
| Eapp | SE | -0.1429686536 | 0.017982018 | ENSMUSG00000054302;SE:12:54673850-54678510:54678623-54685860:- |  |
| Cnnm2 | SE | -0.1631128635 | 0.0184815185 | ENSMUSG00000064105;SE:19:46868631-46871656:46871721-46877208:+ |  |
| Rhot1 | SE | -0.2034774429 | 0.0186480186 | ENSMUSG00000017686;SE:11:80254862-80257514:80257636-80265742:+ |  |
| Rhot1 | SE | -0.1520002234 | 0.0186480186 | ENSMUSG00000017686;SE:11:80255957-80257514:80257636-80265742:+ |  |
| Selenoi | SE | -0.1734024339 | 0.0186480186 | ENSMUSG00000075703;SE:5:30247531-30248412:30248520-30252710:+ |  |
| Selenoi | SE | -0.1202892601 | 0.0186480186 | ENSMUSG00000075703;SE:5:30248520-30252710:30252784-30256078:+ |  |
| Mff | SE | -0.1474001862 | 0.0187312687 | ENSMUSG00000026150;SE:1:82735507-82747083:82747142-82750520:+ |  |
| Mia2 | SE | -0.2174439089 | 0.018981019 | ENSMUSG00000021000;SE:12:59144770-59146336:59146362-59146847:+ |  |
| Nsfl1c | SE | -0.102189624 | 0.018981019 | ENSMUSG00000027455;SE:2:151500789-151502455:151502460-151503014:+ |  |
| Heg1 | SE | 0.1636137803 | 0.018981019 | ENSMUSG00000075254;SE:16:33684855-33706916:33707206-33710400:+ |  |
| Wsb1 | SE | 0.1086074266 | 0.01998002 | ENSMUSG00000017677;SE:11:79246301-79248391:79248659-79250981:- |  |
| Atp5c1 | SE | 0.1498016238 | 0.01998002 | ENSMUSG00000025781;SE:2:10056161-10056770:10056806-10058996:- |  |
| Atp11a | SE | -0.2862068321 | 0.01998002 | ENSMUSG00000031441;SE:8:12859479-12861640:12861724-12861912:+ |  |
| Adgrg6 | SE | 0.1436765819 | 0.01998002 | ENSMUSG00000039116;SE:10:14450664-14456138:14456221-14458080:- |  |
| Ttc19 | SE | -0.1903286133 | 0.01998002 | ENSMUSG00000042298;SE:11:62283707-62284131:62284169-62285003:+ |  |
| Lrrc40 | SE | 0.3436155908 | 0.01998002 | ENSMUSG00000063052;SE:3:158040622-158041620:158041659-158043561:+ |  |
| Serpinh1 | SE | 0.1773056601 | 0.01998002 | ENSMUSG00000070436;SE:7:99349464-99351824:99351867-99353053:- |  |
| Myl6 | SE | 0.3254257535 | 0.0202297702 | ENSMUSG00000090841;SE:10:128492402-128492602:128492709-128493539:- |  |
| Tcof1 | SE | 0.1937482942 | 0.020979021 | ENSMUSG00000024613;SE:18:60822990-60827340:60827447-60828365:- |  |
| Atrip | SE | -0.2183091843 | 0.020979021 | ENSMUSG00000025646;SE:9:109060512-109060629:109060709-109061110:- |  |
| Pphln1 | SE | 0.1548908161 | 0.020979021 | ENSMUSG00000036167;SE:15:93410783-93420284:93420490-93424014:+ |  |
| Gosr1 | SE | -0.1068621655 | 0.021978022 | ENSMUSG00000010392;SE:11:76754800-76761235:76761355-76763520:- |  |
| Iws1 | SE | 0.1672137655 | 0.0224775225 | ENSMUSG00000024384;SE:18:32093229-32093539:32093697-32097150:+ |  |
| Mob4 | SE | -0.1056089724 | 0.0224775225 | ENSMUSG00000025979;SE:1:55136739-55145124:55145224-55148319:+ |  |
| Cdc42bpa | SE | -0.1639971195 | 0.0224775225 | ENSMUSG00000026490;SE:1:180117743-180123924:180123962-180134297:+ |  |
| Asph | SE | -0.1741188602 | 0.0224775225 | ENSMUSG00000028207;SE:4:9598301-9598733:9598780-9601307:- |  |
| Acer3 | SE | 0.1624132256 | 0.0224775225 | ENSMUSG00000030760;SE:7:98226500-98230450:98230613-98243949:- |  |
| Picalm | SE | -0.1264742332 | 0.0224775225 | ENSMUSG00000039361;SE:7:90191753-90194273:90194296-90195655:+ |  |
| Maml1 | SE | 0.1167358505 | 0.0224775225 | ENSMUSG00000050567;SE:11:50258828-50260977:50261073-50263250:- |  |
| Rps27 | SE | 0.1252452567 | 0.0224775225 | ENSMUSG00000090733;SE:3:90213301-90213381:90213493-90213610:- |  |
| Socs6 | SE | 0.1658964182 | 0.0227272727 | ENSMUSG00000056153;SE:18:88870918-88886992:88887065-88887209:- |  |
| Ddx51 | SE | -0.1607984598 | 0.022977023 | ENSMUSG00000029504;SE:5:110656917-110657088:110657151-110657251:+ |  |
| Tpm1 | SE | 0.1430011982 | 0.022977023 | ENSMUSG00000032366;SE:9:67031514-67032466:67032541-67032794:- |  |
| AC175538.1 | SE | 0.3016583433 | 0.022977023 | ENSMUSG00000114470;SE:13:115088961-115092621:115092802-115101471:- |  |
| Taf6l | SE | 0.2094345493 | 0.023976024 | ENSMUSG00000003680;SE:19:8783994-8785122:8785178-8786210:- |  |
| Cd68 | SE | 0.4225658352 | 0.023976024 | ENSMUSG00000018774;SE:11:69665366-69665503:69665936-69666020:- |  |
| Pon3 | SE | -0.1237826246 | 0.023976024 | ENSMUSG00000029759;SE:6:5246333-5254516:5254586-5256153:- |  |
| Dmwd | SE | -0.1186893042 | 0.023976024 | ENSMUSG00000030410;SE:7:19081301-19081919:19081993-19082276:+ |  |
| Opa1 | SE | 0.1543054946 | 0.023976024 | ENSMUSG00000038084;SE:16:29588359-29588823:29588876-29589681:+ |  |
| Arglu1 | SE | 0.1290277056 | 0.023976024 | ENSMUSG00000040459;SE:8:8681467-8681841:8681948-8683748:- |  |
| Naa25 | SE | -0.1501225243 | 0.023976024 | ENSMUSG00000042719;SE:5:121398062-121407193:121407278-121408197:+ |  |
| Rnmt | SE | -0.2035960497 | 0.0244755245 | ENSMUSG00000009535;SE:18:68313846-68313988:68314152-68317994:+ |  |
| Phkb | SE | 0.2898991037 | 0.0244755245 | ENSMUSG00000036879;SE:8:85841103-85843021:85843112-85875634:+ |  |
| Gtpbp8 | SE | -0.2181953311 | 0.0246420246 | ENSMUSG00000022668;SE:16:44743868-44745283:44745493-44745999:- |  |
| Gtpbp8 | SE | -0.1451638771 | 0.0246420246 | ENSMUSG00000022668;SE:16:44743868-44745395:44745493-44745999:- |  |
| Adgrg2 | SE | 0.2572831089 | 0.0247252747 | ENSMUSG00000031298;SE:X:160438313-160456005:160456037-160461343:+ |  |
| Adgrg2 | SE | -0.2078510433 | 0.0247252747 | ENSMUSG00000031298;SE:X:160456037-160458835:160458873-160461343:+ |  |
| Adgrg2 | SE | -0.1414329179 | 0.0247252747 | ENSMUSG00000031298;SE:X:160461381-160463328:160463369-160465578:+ |  |
| Endov | SE | 0.1341062483 | 0.0247752248 | ENSMUSG00000039850;SE:11:119499661-119499846:119499885-119500591:+ |  |
| Lrp8 | SE | 0.2019281415 | 0.024975025 | ENSMUSG00000028613;SE:4:107843344-107846239:107846619-107847444:+ |  |
| Lrp8 | SE | 0.1896166449 | 0.024975025 | ENSMUSG00000028613;SE:4:107869150-107870012:107870188-107872273:+ |  |
| Ldah | SE | 0.3173614956 | 0.024975025 | ENSMUSG00000037669;SE:12:8220755-8238443:8238615-8268364:+ |  |
| Scube3 | SE | 0.1035952274 | 0.024975025 | ENSMUSG00000038677;SE:17:28157189-28158881:28158928-28160812:+ |  |
| Rbfox2 | SE | -0.1148451711 | 0.0253080253 | ENSMUSG00000033565;SE:15:77086155-77091812:77091854-77097940:- |  |
| Smc5 | SE | -0.1056537832 | 0.025974026 | ENSMUSG00000024943;SE:19:23214740-23215155:23215199-23218405:- |  |
| Midn | SE | 0.1026438379 | 0.025974026 | ENSMUSG00000035621;SE:10:80151723-80153591:80153719-80153816:+ |  |
| Rsrp1 | SE | 0.1564737245 | 0.026973027 | ENSMUSG00000037266;SE:4:134925432-134925777:134925821-134926735:+ |  |
| Mpp6 | SE | 0.1770345976 | 0.026973027 | ENSMUSG00000038388;SE:6:50110464-50143719:50143784-50145774:+ |  |
| Sptan1 | SE | 0.1146002699 | 0.0274725275 | ENSMUSG00000057738;SE:2:30000062-30001100:30001159-30002231:+ |  |
| Spopl | SE | -0.1193869079 | 0.0277222777 | ENSMUSG00000026771;SE:2:23545274-23545444:23545579-23571843:- |  |
| Lsm4 | SE | 0.1492925942 | 0.0283050283 | ENSMUSG00000031848;SE:8:70676596-70677758:70677876-70677954:+ |  |
| Lsm4 | SE | 0.1062738485 | 0.0283050283 | ENSMUSG00000031848;SE:8:70676596-70677778:70677858-70677954:+ |  |
| Lsm4 | SE | 0.1266629006 | 0.0283050283 | ENSMUSG00000031848;SE:8:70676596-70677778:70677876-70677954:+ |  |
| Sms | SE | -0.1578199394 | 0.0284715285 | ENSMUSG00000071708;SE:X:157454913-157455994:157456108-157460327:- |  |
| Bcl2l13 | SE | -0.1070053703 | 0.028971029 | ENSMUSG00000009112;SE:6:120848616-120862855:120862962-120865568:+ |  |
| Ppp2r5e | SE | -0.1057952523 | 0.02997003 | ENSMUSG00000021051;SE:12:75485224-75493414:75493515-75515602:- |  |
| Vrk1 | SE | 0.3054833858 | 0.02997003 | ENSMUSG00000021115;SE:12:106070615-106071570:106071641-106073619:+ |  |
| Pex2 | SE | 0.1932252032 | 0.02997003 | ENSMUSG00000040374;SE:3:5561764-5562669:5562732-5563165:- |  |
| Usp48 | SE | 0.1954874528 | 0.02997003 | ENSMUSG00000043411;SE:4:137635008-137636934:137636987-137637760:+ |  |
| Pdp1 | SE | -0.1175315675 | 0.02997003 | ENSMUSG00000049225;SE:4:11962233-11965614:11965648-11965884:- |  |
| Scnm1 | SE | 0.1715293217 | 0.02997003 | ENSMUSG00000092607;SE:3:95129883-95130164:95130358-95132825:- |  |
| Slc25a51 | SE | -0.1114802295 | 0.0312187812 | ENSMUSG00000045973;SE:4:45400179-45404727:45404835-45408695:- |  |
| Abcc4 | SE | 0.1416942726 | 0.0314685315 | ENSMUSG00000032849;SE:14:118632187-118654150:118654374-118668347:- |  |
| Trub2 | SE | 0.2118124984 | 0.0314685315 | ENSMUSG00000039826;SE:2:29782201-29783312:29783373-29784953:- |  |
| Kdm5c | SE | -0.137277618 | 0.031968032 | ENSMUSG00000025332;SE:X:152237710-152239630:152239752-152240026:+ |  |
| sep-02 | SE | 0.3293177485 | 0.031968032 | ENSMUSG00000026276;SE:1:93479068-93489128:93489154-93491157:+ |  |
| Tmem104 | SE | -0.2442332211 | 0.031968032 | ENSMUSG00000045980;SE:11:115205583-115228421:115228514-115243370:+ |  |
| 0610010K14Rik | SE | 0.1212666141 | 0.0324675325 | ENSMUSG00000020831;SE:11:70236211-70236877:70236978-70237531:- |  |
| Porcn | SE | 0.3714323031 | 0.0324675325 | ENSMUSG00000031169;SE:X:8203835-8204196:8204388-8205565:- |  |
| Col18a1 | SE | -0.1062357834 | 0.032967033 | ENSMUSG00000001435;SE:10:77055827-77057369:77057401-77058699:- |  |
| 9430038I01Rik | SE | -0.1649029093 | 0.0334665335 | ENSMUSG00000040139;SE:7:137376351-137376955:137377117-137387371:- |  |
| Gm20498 | SE | -0.1286227056 | 0.0337162837 | ENSMUSG00000021139;SE:12:81481002-81489175:81489244-81504544:- |  |
| Gm20498 | SE | -0.1271197029 | 0.0337162837 | ENSMUSG00000021139;SE:12:81504639-81510829:81510965-81532716:- |  |
| Ptprk | SE | -0.1369705662 | 0.033966034 | ENSMUSG00000019889;SE:10:28560165-28562107:28562142-28566495:+ |  |
| Phactr2 | SE | -0.2032949232 | 0.033966034 | ENSMUSG00000062866;SE:10:13253879-13255310:13255321-13261766:- |  |
| Wbp1l | SE | 0.149950964 | 0.034965035 | ENSMUSG00000047731;SE:19:46599293-46606712:46606759-46644353:+ |  |
| Adgrg2 | SE | -0.1596437545 | 0.0351648352 | ENSMUSG00000031298;SE:X:160456037-160458835:160458882-160461343:+ |  |
| Sipa1l1 | SE | 0.1267645704 | 0.0352147852 | ENSMUSG00000042700;SE:12:82234528-82265957:82266025-82311349:+ |  |
| Ablim1 | SE | 0.2663287151 | 0.035964036 | ENSMUSG00000025085;SE:19:57044972-57046925:57047065-57049462:- |  |
| Ablim1 | SE | 0.4460148791 | 0.035964036 | ENSMUSG00000025085;SE:19:57044972-57046925:57047071-57049462:- |  |
| Ablim1 | SE | -0.2753453012 | 0.035964036 | ENSMUSG00000025085;SE:19:57049509-57051130:57051234-57061253:- |  |
| Papss1 | SE | -0.1074934634 | 0.035964036 | ENSMUSG00000028032;SE:3:131564983-131567502:131567678-131579311:+ |  |
| Akap13 | SE | 0.1687499097 | 0.035964036 | ENSMUSG00000066406;SE:7:75677479-75679820:75679873-75685117:+ |  |
| Dtnb | SE | -0.352538505 | 0.035964036 | ENSMUSG00000071454;SE:12:3751907-3754096:3754116-3772587:+ |  |
| Lifr | SE | 0.1229641171 | 0.0364635365 | ENSMUSG00000054263;SE:15:7129826-7130901:7131038-7154387:+ |  |
| Ift22 | SE | -0.1107862414 | 0.036963037 | ENSMUSG00000007987;SE:5:136911200-136911302:136911391-136911689:+ |  |
| Hars2 | SE | -0.1122467525 | 0.036963037 | ENSMUSG00000019143;SE:18:36789284-36789367:36789609-36790134:+ |  |
| Preb | SE | -0.153722224 | 0.0372960373 | ENSMUSG00000045302;SE:5:30955737-30955930:30956089-30956262:- |  |
| Preb | SE | -0.1361238785 | 0.0372960373 | ENSMUSG00000045302;SE:5:30956089-30956262:30956334-30957888:- |  |
| Pacs2 | SE | -0.1581160471 | 0.0374625375 | ENSMUSG00000021143;SE:12:113044313-113045478:113045567-113047029:+ |  |
| Mapre2 | SE | -0.17288652 | 0.0374625375 | ENSMUSG00000024277;SE:18:23804221-23832853:23832980-23853654:+ |  |
| Tm7sf2 | SE | -0.154238652 | 0.0374625375 | ENSMUSG00000024799;SE:19:6067022-6067123:6067319-6067669:- |  |
| Ppp6c | SE | -0.1274118757 | 0.0374625375 | ENSMUSG00000026753;SE:2:39204974-39206652:39206717-39210958:- |  |
| Nusap1 | SE | 0.1201901851 | 0.0374625375 | ENSMUSG00000027306;SE:2:119630505-119633720:119633818-119635478:+ |  |
| Ilf3 | SE | 0.1402106598 | 0.0374625375 | ENSMUSG00000032178;SE:9:21387797-21388112:21388150-21388524:+ |  |
| Sec16b | SE | -0.1128878899 | 0.037962038 | ENSMUSG00000026589;SE:1:157506860-157507386:157507553-157524785:+ |  |
| Ccp110 | SE | -0.1326413408 | 0.037962038 | ENSMUSG00000033904;SE:7:118732453-118732887:118732972-118735268:+ |  |
| Itpa | SE | -0.4173583868 | 0.037962038 | ENSMUSG00000074797;SE:2:130671620-130672060:130672338-130674239:+ |  |
| Brd8 | SE | 0.1127318171 | 0.038961039 | ENSMUSG00000003778;SE:18:34608055-34608418:34608627-34609805:- |  |
| Tbl2 | SE | -0.1675894893 | 0.038961039 | ENSMUSG00000005374;SE:5:135154498-135156368:135156519-135157532:+ |  |
| Cbx5 | SE | -0.116578923 | 0.038961039 | ENSMUSG00000009575;SE:15:103213254-103215022:103215125-103215268:- |  |
| Cdk2 | SE | 0.1351266237 | 0.03996004 | ENSMUSG00000025358;SE:10:128699675-128699976:128700119-128701343:- |  |
| Gtf2a2 | SE | -0.1563070304 | 0.03996004 | ENSMUSG00000033543;SE:9:70012661-70014293:70014491-70015292:+ |  |
| Myo5a | SE | -0.1002813223 | 0.03996004 | ENSMUSG00000034593;SE:9:75194137-75196126:75196200-75197643:+ |  |
| Fgfr1op | SE | 0.1323006887 | 0.03996004 | ENSMUSG00000069135;SE:17:8173008-8175519:8175578-8182228:+ |  |
| Nmt2 | SE | -0.1044892444 | 0.040959041 | ENSMUSG00000026643;SE:2:3305439-3306983:3307032-3309467:+ |  |
| Guf1 | SE | -0.1225677731 | 0.0412087912 | ENSMUSG00000029208;SE:5:69565562-69566361:69566504-69567128:+ |  |
| Arfip2 | SE | 0.4220439958 | 0.0412087912 | ENSMUSG00000030881;SE:7:105637278-105637652:105637725-105637863:- |  |
| Ints7 | SE | -0.1301501828 | 0.0412087912 | ENSMUSG00000037461;SE:1:191575951-191582399:191582528-191583179:+ |  |
| Ints7 | SE | 0.1443122157 | 0.0412087912 | ENSMUSG00000037461;SE:1:191575951-191583179:191583325-191586524:+ |  |
| Rbm25 | SE | -0.2729238019 | 0.041958042 | ENSMUSG00000010608;SE:12:83639191-83642402:83642519-83642629:+ |  |
| Mtr | SE | -0.1954507824 | 0.041958042 | ENSMUSG00000021311;SE:13:12247985-12249845:12249914-12250602:- |  |
| Tmem33 | SE | -0.1020762509 | 0.041958042 | ENSMUSG00000037720;SE:5:67261033-67263730:67263824-67264270:+ |  |
| Tmem33 | SE | -0.1138895232 | 0.041958042 | ENSMUSG00000037720;SE:5:67263824-67264270:67264457-67267302:+ |  |
| Abi1 | SE | -0.1785524358 | 0.041958042 | ENSMUSG00000058835;SE:2:22962501-22963214:22963228-22971083:- |  |
| Polr1a | SE | 0.1039299114 | 0.0424575425 | ENSMUSG00000049553;SE:6:71924697-71924968:71925039-71926284:+ |  |
| Sipa1 | SE | 0.5352650571 | 0.0424575425 | ENSMUSG00000056917;SE:19:5661111-5661596:5661626-5662588:- |  |
| Cenpa | SE | -0.2437605787 | 0.0427072927 | ENSMUSG00000029177;SE:5:30673059-30673267:30673443-30674038:+ |  |
| Map2k7 | SE | -0.115889984 | 0.042957043 | ENSMUSG00000002948;SE:8:4239033-4240688:4240735-4243304:+ |  |
| Zfp518b | SE | 0.1424996821 | 0.042957043 | ENSMUSG00000046572;SE:5:38682782-38683193:38683258-38684679:- |  |
| Entpd5 | SE | 0.2577390305 | 0.0434565435 | ENSMUSG00000021236;SE:12:84396958-84399340:84399412-84405406:- |  |
| Sipa1 | SE | -0.1422150846 | 0.0437062937 | ENSMUSG00000056917;SE:19:5661111-5662588:5662841-5663604:- |  |
| Sipa1 | SE | 0.1880851698 | 0.0437062937 | ENSMUSG00000056917;SE:19:5661626-5662588:5662841-5663604:- |  |
| Ppig | SE | 0.1449299824 | 0.043956044 | ENSMUSG00000042133;SE:2:69723363-69731491:69731580-69731746:+ |  |
| Tsen2 | SE | 0.1055751984 | 0.043956044 | ENSMUSG00000042389;SE:6:115548030-115549913:115549994-115553991:+ |  |
| Ywhag | SE | -0.3309166894 | 0.043956044 | ENSMUSG00000051391;SE:5:135930949-135932517:135932642-135934323:- |  |
| Dnajc1 | SE | -0.1890224568 | 0.044955045 | ENSMUSG00000026740;SE:2:18308949-18316358:18316404-18316899:- |  |
| Zbtb49 | SE | 0.3059546717 | 0.044955045 | ENSMUSG00000029127;SE:5:38210664-38211846:38212088-38213308:- |  |
| Zbtb49 | SE | -0.2711139369 | 0.044955045 | ENSMUSG00000029127;SE:5:38210664-38213308:38214383-38216447:- |  |
| Cox5b | SE | 0.3234961519 | 0.044955045 | ENSMUSG00000061518;SE:1:36692284-36692431:36692550-36693197:+ |  |
| Btrc | SE | -0.1824439816 | 0.045954046 | ENSMUSG00000025217;SE:19:45363886-45423133:45423240-45456533:+ |  |
| sep-11 | SE | 0.1184037498 | 0.0460789211 | ENSMUSG00000058013;SE:5:93167687-93173470:93173537-93176040:+ |  |
| Ubap1 | SE | -0.1207081734 | 0.0464535465 | ENSMUSG00000028437;SE:4:41379870-41387175:41387357-41388096:+ |  |
| Acp2 | SE | -0.1156901294 | 0.046953047 | ENSMUSG00000002103;SE:2:91205908-91206010:91206108-91206188:+ |  |
| Mia2 | SE | 0.2007332157 | 0.0474525475 | ENSMUSG00000021000;SE:12:59137786-59144699:59144770-59146847:+ |  |
| Usp3 | SE | 0.129757212 | 0.0474525475 | ENSMUSG00000032376;SE:9:66547955-66566843:66566903-66592784:- |  |
| Slc25a36 | SE | 0.1875545154 | 0.0474525475 | ENSMUSG00000032449;SE:9:97085123-97090093:97090174-97093077:- |  |
| 2010111I01Rik | SE | -0.2613605723 | 0.047952048 | ENSMUSG00000021458;SE:13:63298856-63300605:63300793-63301122:+ |  |
| Samd4 | SE | 0.1238406232 | 0.047952048 | ENSMUSG00000021838;SE:14:47016795-47052850:47053113-47064220:+ |  |
| Gmeb1 | SE | -0.1876445996 | 0.047952048 | ENSMUSG00000028901;SE:4:132251832-132252431:132252493-132261491:- |  |
| Endov | SE | -0.1299277351 | 0.047952048 | ENSMUSG00000039850;SE:11:119491956-119499527:119499661-119499846:+ |  |
| Zfp68 | SE | 0.1171388988 | 0.047952048 | ENSMUSG00000058291;SE:5:138612165-138616470:138616559-138617055:- |  |
| Ttc19 | SE | -0.2413127794 | 0.0487012987 | ENSMUSG00000042298;SE:11:62313178-62315877:62315968-62327898:+ |  |
| Mtch2 | SE | 0.1337798294 | 0.048951049 | ENSMUSG00000027282;SE:2:90849704-90852816:90852866-90853051:+ |  |
| Phkb | SE | 0.1170875121 | 0.048951049 | ENSMUSG00000036879;SE:8:85841103-85843004:85843112-85875634:+ |  |
| Bet1l | SE | 0.2283632384 | 0.04995005 | ENSMUSG00000025484;SE:7:140854623-140854765:140854801-140855074:- |  |
| Brd3 | SE | 0.1336317475 | 0.04995005 | ENSMUSG00000026918;SE:2:27453074-27453400:27453450-27454313:- |  |
| Hnrnph1 | RI | -0.2556387479 | 0 | ENSMUSG00000007850;RI:11:50379821:50379964-50382537:50382715:+ |  |
| Cdkn1b | RI | -0.18221675 | 0.0024975025 | ENSMUSG00000003031;RI:6:134921956:134922083-134924243:134925513:+ |  |
| Lss | RI | -0.1969407817 | 0.003996004 | ENSMUSG00000033105;RI:10:76535559:76535667-76536244:76536365:+ |  |
| Arglu1 | RI | -0.1819996431 | 0.0044955045 | ENSMUSG00000040459;RI:8:8681384:8681467-8683748:8683973:- |  |
| Eif5b | RI | 0.1501732556 | 0.007992008 | ENSMUSG00000026083;RI:1:38033068:38033157-38034029:38034103:+ |  |
| Eif5b | RI | 0.1501732556 | 0.007992008 | ENSMUSG00000026083;RI:1:38050187:38050325-38051123:38051284:+ |  |
| Srsf5 | RI | -0.1235960667 | 0.0081585082 | ENSMUSG00000021134;RI:12:80947796:80947865-80949095:80949168:+ |  |
| Elk3 | RI | -0.1379706432 | 0.008991009 | ENSMUSG00000008398;RI:10:93264880:93265010-93265337:93265680:- |  |
| Rbm5 | RI | -0.3324595749 | 0.0094905095 | ENSMUSG00000032580;RI:9:107756909:107756992-107760322:107760391:- |  |
| Pomt2 | RI | 0.2271627334 | 0.0094905095 | ENSMUSG00000034126;RI:12:87111337:87111442-87111524:87111583:- |  |
| Zranb2 | RI | -0.1133219602 | 0.00999001 | ENSMUSG00000028180;RI:3:157541700:157541869-157543158:157543244:+ |  |
| Glo1 | RI | -0.1067737038 | 0.010989011 | ENSMUSG00000024026;RI:17:30592866:30593074-30594115:30594226:- |  |
| Sat2 | RI | -0.2318216779 | 0.010989011 | ENSMUSG00000069835;RI:11:69622052:69622115-69622256:69622332:+ |  |
| Gnb4 | RI | 0.2362014527 | 0.011988012 | ENSMUSG00000027669;RI:3:32589709:32589910-32589997:32590063:- |  |
| Miip | RI | -0.3312339845 | 0.011988012 | ENSMUSG00000029022;RI:4:147862438:147862567-147862897:147862949:- |  |
| Npepl1 | RI | -0.1830218306 | 0.011988012 | ENSMUSG00000039263;RI:2:174120544:174120667-174120948:174121124:+ |  |
| Dusp22 | RI | -0.323497206 | 0.011988012 | ENSMUSG00000069255;RI:13:30708677:30708749-30710706:30711231:+ |  |
| Sdccag3 | RI | -0.1978130554 | 0.012987013 | ENSMUSG00000026927;RI:2:26385498:26385609-26385990:26386063:- |  |
| Clk1 | RI | -0.2280350096 | 0.014985015 | ENSMUSG00000026034;RI:1:58419667:58419733-58421083:58421308:- |  |
| Tial1 | RI | -0.1667794183 | 0.014985015 | ENSMUSG00000030846;RI:7:128443807:128443946-128444281:128444404:- |  |
| Selenoh | RI | -0.5138870159 | 0.014985015 | ENSMUSG00000076437;RI:2:84670245:84670390-84670483:84670676:- |  |
| Ubp1 | RI | -0.148832464 | 0.015984016 | ENSMUSG00000009741;RI:9:113955963:113956068-113956707:113956813:+ |  |
| Cdk11b | RI | -0.1617649663 | 0.0162337662 | ENSMUSG00000029062;RI:4:155625531:155625654-155625727:155625773:+ |  |
| Luc7l | RI | -0.2872684794 | 0.017982018 | ENSMUSG00000024188;RI:17:26253974:26254044-26255031:26255125:+ |  |
| Zranb2 | RI | -0.2108870775 | 0.0181068931 | ENSMUSG00000028180;RI:3:157541700:157541821-157543158:157543244:+ |  |
| Cinp | RI | -0.6417430492 | 0.020979021 | ENSMUSG00000021276;RI:12:110878696:110879039-110879685:110879814:- |  |
| Surf1 | RI | -0.2601293247 | 0.023976024 | ENSMUSG00000015790;RI:2:26915596:26915749-26915955:26916006:- |  |
| Idh3g | RI | -0.170771328 | 0.0266400266 | ENSMUSG00000002010;RI:X:73782006:73782103-73782675:73782716:- |  |
| Taf1d | RI | -0.2746829044 | 0.027972028 | ENSMUSG00000031939;RI:9:15306873:15306990-15307728:15307821:+ |  |
| Rpl3 | RI | -0.1301380898 | 0.027972028 | ENSMUSG00000060036;RI:15:80080896:80081211-80081615:80081783:- |  |
| Mlycd | RI | 0.1759807697 | 0.0284715285 | ENSMUSG00000074064;RI:8:119401481:119401593-119402434:119402590:+ |  |
| Ppp1cc | RI | -0.2512845738 | 0.02997003 | ENSMUSG00000004455;RI:5:122172732:122172803-122173146:122173280:+ |  |
| Rsf1 | RI | 0.1671352633 | 0.02997003 | ENSMUSG00000035623;RI:7:97669769:97669873-97670803:97670882:+ |  |
| Gga2 | RI | -0.1321604294 | 0.0304695305 | ENSMUSG00000030872;RI:7:121986722:121987664-121988274:121989801:- |  |
| Rnf126 | RI | -0.1043463553 | 0.031968032 | ENSMUSG00000035890;RI:10:79761430:79761499-79761565:79761633:- |  |
| Tgif2 | RI | -0.1734370589 | 0.035964036 | ENSMUSG00000062175;RI:2:156853129:156853228-156853346:156855570:+ |  |
| Arhgef40 | RI | -0.2723317687 | 0.03996004 | ENSMUSG00000004562;RI:14:52004879:52004899-52004989:52005035:+ |  |
| Guf1 | RI | -0.1203055204 | 0.0412087912 | ENSMUSG00000029208;RI:5:69567128:69567261-69568396:69568497:+ |  |
| Snrnp48 | RI | -0.1023503756 | 0.0414585415 | ENSMUSG00000021431;RI:13:38217374:38217495-38220688:38220776:+ |  |
| Tmem208 | RI | -0.1034901423 | 0.041958042 | ENSMUSG00000014856;RI:8:105328322:105328458-105328608:105328692:+ |  |
| Gramd1a | RI | -0.2363342056 | 0.044955045 | ENSMUSG00000001248;RI:7:31134414:31134517-31135597:31135739:- |  |
| 1810022K09Rik | RI | -0.1703264998 | 0.045954046 | ENSMUSG00000078784;RI:3:14606289:14606408-14607177:14607293:- |  |
| Cdan1 | RI | 0.2093450444 | 0.0464535465 | ENSMUSG00000027284;RI:2:120729689:120729802-120729930:120730099:- |  |
| Acp2 | RI | 0.1000198477 | 0.046953047 | ENSMUSG00000002103;RI:2:91206188:91206277-91206717:91206849:+ |  |
| Nubp1 | RI | -0.499544296 | 0.047952048 | ENSMUSG00000022503;RI:16:10419653:10419685-10420974:10421128:+ |  |
| H2afy | MX | -0.4490674747 | 0 | ENSMUSG00000015937;MX:13:56084285-56088252:56088342-56096124:56084285-56089758:56089857-56096124:- |  |
| Thsd7a | MX | 0.2670998059 | 0.003996004 | ENSMUSG00000032625;MX:6:12348330-12349710:12349715-12351985:12348330-12351612:12351617-12351985:- |  |
| Flot2 | MX | -0.148282312 | 0.008991009 | ENSMUSG00000061981;MX:11:78049559-78053363:78053453-78055757:78049559-78054785:78054875-78055757:+ |  |
| Rbm39 | MX | -0.1976639309 | 0.0110603682 | ENSMUSG00000027620;MX:2:156177385-156177752:156177906-156179177:156177385-156178880:156178952-156179177:- |  |
| Ubr2 | MX | 0.1941528036 | 0.0124875125 | ENSMUSG00000023977;MX:17:46976090-46980320:46980418-46981354:46976090-46980489:46980587-46981354:- |  |
| Sar1a | MX | -0.1805178407 | 0.0134865135 | ENSMUSG00000020088;MX:10:61680413-61682099:61682148-61684880:61680413-61684234:61684331-61684880:+ |  |
| Sar1a | MX | -0.196268678 | 0.0134865135 | ENSMUSG00000020088;MX:10:61680413-61684061:61684187-61684880:61680413-61684234:61684331-61684880:+ |  |
| Dab2 | MX | 0.2283199385 | 0.0153180153 | ENSMUSG00000022150;MX:15:6424250-6424635:6424697-6430954:6424250-6429296:6429949-6430954:+ |  |
| Rbfox2 | MX | -0.1060386091 | 0.015984016 | ENSMUSG00000033565;MX:15:77086155-77091812:77091854-77097940:77086155-77094451:77094490-77097940:- |  |
| Tpm2 | MX | -0.1006583792 | 0.017982018 | ENSMUSG00000028464;MX:4:43518464-43518644:43518719-43519227:43518464-43519003:43519078-43519227:- |  |
| Rhot1 | MX | 0.1660119507 | 0.0186480186 | ENSMUSG00000017686;MX:11:80254862-80255862:80255957-80265742:80254862-80257514:80257636-80265742:+ |  |
| Cd99l2 | MX | 0.2741567807 | 0.021978022 | ENSMUSG00000035776;MX:X:71441145-71449939:71450007-71492522:71441145-71450611:71450673-71492522:- |  |
| Mob4 | MX | 0.1364946878 | 0.0224775225 | ENSMUSG00000025979;MX:1:55131359-55136677:55136739-55148319:55131359-55145124:55145224-55148319:+ |  |
| Pkm | MX | -0.1159534495 | 0.023976024 | ENSMUSG00000032294;MX:9:59672073-59675029:59675195-59678044:59672073-59675568:59675734-59678044:+ |  |
| Tmub2 | MX | 0.1681795122 | 0.0262237762 | ENSMUSG00000034757;MX:11:102285088-102285664:102285718-102287308:102285088-102285980:102286116-102287308:+ |  |
| Lrrc40 | MX | 0.2205516031 | 0.027972028 | ENSMUSG00000063052;MX:3:158040622-158041620:158041659-158043561:158040622-158041863:158041933-158043561:+ |  |
| Rbm25 | MX | 0.3645836601 | 0.02997003 | ENSMUSG00000010608;MX:12:83632417-83639128:83639191-83642629:83632417-83642402:83642519-83642629:+ |  |
| Nbr1 | MX | 0.2698521043 | 0.02997003 | ENSMUSG00000017119;MX:11:101576409-101577194:101577251-101580585:101576409-101577932:101578037-101580585:+ |  |
| Mtdh | MX | 0.5317093449 | 0.0314685315 | ENSMUSG00000022255;MX:15:34114108-34114799:34114975-34117880:34114108-34116311:34116376-34117880:+ |  |
| Endov | MX | -0.504125957 | 0.0352980353 | ENSMUSG00000039850;MX:11:119491956-119499527:119499661-119500591:119491956-119499846:119499885-119500591:+ |  |
| Nkiras1 | MX | -0.5349903492 | 0.038961039 | ENSMUSG00000021772;MX:14:18271323-18276696:18276806-18278304:18271323-18276826:18276968-18278304:+ |  |
| Sipa1 | MX | 0.1857472963 | 0.0437062937 | ENSMUSG00000056917;MX:19:5661111-5661596:5661626-5663604:5661111-5662588:5662841-5663604:- |  |
| Stoml2 | MX | 0.1378846578 | 0.044955045 | ENSMUSG00000028455;MX:4:43030298-43030436:43030535-43031329:43030298-43030971:43031108-43031329:- |  |
| Spcs2 | MX | 0.1003038962 | 0.0466200466 | ENSMUSG00000035227;MX:7:99849059-99849157:99849252-99858764:99849059-99856413:99856514-99858764:- |  |
| Tcf25 | AL | 0.2146074379 | 0 | ENSMUSG00000001472;AL:8:123400697-123401076:123401231:123400697-123403147:123403806:+ |  |
| Tcf25 | AL | 0.2101945463 | 0 | ENSMUSG00000001472;AL:8:123400697-123401076:123401892:123400697-123403147:123403806:+ |  |
| Ube2j1 | AL | 0.2956069191 | 0 | ENSMUSG00000028277;AL:4:33043986-33045080:33045199:33043986-33049682:33052363:+ |  |
| Tpm1 | AL | 0.2977220238 | 0 | ENSMUSG00000032366;AL:9:67022592:67023441-67031029:67024324:67024565-67031029:- |  |
| Tpm1 | AL | 0.3345179349 | 0 | ENSMUSG00000032366;AL:9:67022592:67023441-67031029:67027989:67028167-67031029:- |  |
| Tpm1 | AL | 0.3605720506 | 0 | ENSMUSG00000032366;AL:9:67022592:67023441-67031029:67028848:67029742-67031029:- |  |
| Tpm1 | AL | 0.2057718921 | 0 | ENSMUSG00000032366;AL:9:67022615:67023441-67031029:67027989:67028167-67031029:- |  |
| Cab39l | AL | -0.217907039 | 0.0024975025 | ENSMUSG00000021981;AL:14:59539205-59546986:59548903:59539205-59585504:59585764:+ |  |
| Tcf25 | AL | 0.6363152617 | 0.0028305028 | ENSMUSG00000001472;AL:8:123400697-123401058:123401152:123400697-123403150:123403403:+ |  |
| Pam | AL | 0.1705759155 | 0.002997003 | ENSMUSG00000026335;AL:1:97821721:97821988-97831455:97825659:97826040-97831455:- |  |
| Tpm1 | AL | 0.1743575206 | 0.0038295038 | ENSMUSG00000032366;AL:9:67022615:67023441-67031029:67024324:67024565-67031029:- |  |
| Tpm1 | AL | 0.2181069109 | 0.0038295038 | ENSMUSG00000032366;AL:9:67022615:67023441-67031029:67028848:67029742-67031029:- |  |
| Tcf25 | AL | 0.5834193322 | 0.0063686314 | ENSMUSG00000001472;AL:8:123400697-123401058:123401152:123400697-123403150:123403835:+ |  |
| Sorcs1 | AL | -0.251154467 | 0.006993007 | ENSMUSG00000043531;AL:19:50147660:50151351-50153054:50152692:50152920-50153054:- |  |
| Endov | AL | 0.4261117831 | 0.0106560107 | ENSMUSG00000039850;AL:11:119505167-119505588:119505962:119505167-119507202:119507691:+ |  |
| Endov | AL | 0.4323446052 | 0.0106560107 | ENSMUSG00000039850;AL:11:119505167-119505588:119505962:119505167-119507202:119511423:+ |  |
| Endov | AL | -0.3962395119 | 0.0114171543 | ENSMUSG00000039850;AL:11:119505167-119505588:119505962:119505167-119507202:119507751:+ |  |
| Endov | AL | 0.4088485575 | 0.0114171543 | ENSMUSG00000039850;AL:11:119505167-119505588:119505962:119505167-119507202:119511437:+ |  |
| Szrd1 | AL | -0.1499465237 | 0.0124875125 | ENSMUSG00000040842;AL:4:141113001:141115864-141118527:141116567:141116640-141118527:- |  |
| Tcf25 | AL | 0.155167475 | 0.016983017 | ENSMUSG00000001472;AL:8:123400697-123401076:123401231:123400697-123403150:123403403:+ |  |
| Tcf25 | AL | 0.1306043922 | 0.016983017 | ENSMUSG00000001472;AL:8:123400697-123401076:123401231:123400697-123403150:123403806:+ |  |
| Tcf25 | AL | 0.1456413152 | 0.016983017 | ENSMUSG00000001472;AL:8:123400697-123401076:123401231:123400697-123403150:123403835:+ |  |
| Tcf25 | AL | 0.1475355876 | 0.016983017 | ENSMUSG00000001472;AL:8:123400697-123401076:123401892:123400697-123403150:123403403:+ |  |
| Tcf25 | AL | 0.1259394336 | 0.016983017 | ENSMUSG00000001472;AL:8:123400697-123401076:123401892:123400697-123403150:123403806:+ |  |
| Tcf25 | AL | 0.1430257203 | 0.016983017 | ENSMUSG00000001472;AL:8:123400697-123401076:123401892:123400697-123403150:123403835:+ |  |
| Zfp950 | AL | 0.2838241072 | 0.016983017 | ENSMUSG00000074733;AL:19:61117124:61117288-61127007:61118131:61120452-61127007:- |  |
| Slc35b2 | AL | 0.2565160691 | 0.0174825175 | ENSMUSG00000037089;AL:17:45565079-45565200:45565448:45565079-45566309:45567671:+ |  |
| Slc35b2 | AL | 0.1445530006 | 0.0174825175 | ENSMUSG00000037089;AL:17:45565079-45565664:45566055:45565079-45566309:45567670:+ |  |
| Slc35b2 | AL | 0.2173756605 | 0.0174825175 | ENSMUSG00000037089;AL:17:45565079-45565664:45566055:45565079-45566309:45567671:+ |  |
| Frmd5 | AL | -0.3373978103 | 0.017982018 | ENSMUSG00000027238;AL:2:121545529:121548023-121548889:121548304:121548709-121548889:- |  |
| Dcun1d5 | AL | -0.1191189549 | 0.017982018 | ENSMUSG00000032002;AL:9:7203555-7205298:7205639:7203555-7206720:7207040:+ |  |
| Slc35b2 | AL | 0.1035288014 | 0.017982018 | ENSMUSG00000037089;AL:17:45565079-45565200:45565448:45565079-45566309:45567670:+ |  |
| Mff | AL | -0.2477140693 | 0.0187312687 | ENSMUSG00000026150;AL:1:82735507-82741818:82741884:82735507-82750520:82750581:+ |  |
| Wdr26 | AL | -0.1256160295 | 0.0194805195 | ENSMUSG00000038733;AL:1:181173228:181177831-181181304:181180407:181180648-181181304:- |  |
| Cnot8 | AL | -0.11796215 | 0.01998002 | ENSMUSG00000020515;AL:11:58115375-58117207:58117258:58115375-58117439:58118594:+ |  |
| Atp11a | AL | 0.2707567816 | 0.01998002 | ENSMUSG00000031441;AL:8:12859479-12861912:12862205:12859479-12864920:12868728:+ |  |
| Dcun1d5 | AL | -0.1010324013 | 0.0202297702 | ENSMUSG00000032002;AL:9:7205400-7206073:7206489:7205400-7206720:7208205:+ |  |
| Zfp64 | AL | -0.1234884529 | 0.0214785215 | ENSMUSG00000027551;AL:2:168925361:168926933-168935211:168934813:168934939-168935211:- |  |
| Frmd5 | AL | -0.3884234374 | 0.0224775225 | ENSMUSG00000027238;AL:2:121545529:121547648-121548889:121548304:121548709-121548889:- |  |
| AC175538.1 | AL | -0.3335528115 | 0.022977023 | ENSMUSG00000114470;AL:13:115088356:115088961-115101471:115090054:115090304-115101471:- |  |
| Endov | AL | 0.2831551719 | 0.0230880231 | ENSMUSG00000039850;AL:11:119505167-119505588:119505962:119505167-119507202:119509579:+ |  |
| Dnajc19 | AL | -0.4910098737 | 0.023976024 | ENSMUSG00000027679;AL:3:34056020:34058114-34078775:34077324:34077524-34078775:- |  |
| Polr1e | AL | 0.5347955725 | 0.0274725275 | ENSMUSG00000028318;AL:4:45031498-45031941:45032365:45031498-45036250:45036565:+ |  |
| 1500011B03Rik | AL | -0.1361729965 | 0.0284715285 | ENSMUSG00000072694;AL:5:114808196:114809331-114813780:114812793:114813205-114813780:- |  |
| Cr1l | AL | -0.184107578 | 0.02997003 | ENSMUSG00000016481;AL:1:195097382:195098841-195106818:195103790:195103907-195106818:- |  |
| Ociad1 | AL | 0.2171608798 | 0.02997003 | ENSMUSG00000029152;AL:5:73306753-73310274:73310416:73306753-73313443:73313941:+ |  |
| BC030336 | AL | 0.4017986597 | 0.02997003 | ENSMUSG00000046096;AL:7:120726332-120730477:120730494:120726332-120732365:120734271:+ |  |
| Gm14410 | AL | -0.1687935687 | 0.0304695305 | ENSMUSG00000078870;AL:2:177183453:177184016-177195436:177193357:177194278-177195436:- |  |
| Slc25a51 | AL | 0.2438442619 | 0.0312187812 | ENSMUSG00000045973;AL:4:45399241:45400179-45408695:45407172:45408152-45408695:- |  |
| Samhd1 | AL | -0.1326173082 | 0.0324675325 | ENSMUSG00000027639;AL:2:157097533:157099503-157101721:157101360:157101443-157101721:- |  |
| Aebp2 | AL | -0.2491610705 | 0.033966034 | ENSMUSG00000030232;AL:6:140650782-140651316:140652826:140650782-140653758:140654418:+ |  |
| Steap3 | AL | -0.2060453085 | 0.0346320346 | ENSMUSG00000026389;AL:1:120190757:120190827-120234242:120226653:120227928-120234242:- |  |
| Endov | AL | 0.2608875439 | 0.0348742167 | ENSMUSG00000039850;AL:11:119505167-119505588:119505962:119505167-119507202:119507389:+ |  |
| Plgrkt | AL | 0.2261490306 | 0.034965035 | ENSMUSG00000016495;AL:19:29348677:29349005-29358317:29351034:29351174-29358317:- |  |
| Cinp | AL | -0.1942643489 | 0.034965035 | ENSMUSG00000021276;AL:12:110872610:110874129-110876841:110875034:110875276-110876841:- |  |
| Ppm1b | AL | -0.1318688363 | 0.034965035 | ENSMUSG00000061130;AL:17:85005566-85013566:85014776:85005566-85015409:85017128:+ |  |
| 2610044O15Rik8 | AL | -0.154287033 | 0.035964036 | ENSMUSG00000071302;AL:8:129217543:129217749-129221544:129220095:129220151-129221544:- |  |
| Agap3 | AL | -0.4106169887 | 0.0362137862 | ENSMUSG00000023353;AL:5:24479789-24480123:24480188:24479789-24482266:24483184:+ |  |
| Emc8 | AL | 0.1732329906 | 0.0374625375 | ENSMUSG00000031819;AL:8:120657833:120658248-120667757:120660647:120660860-120667757:- |  |
| Gtf2a2 | AL | -0.1983336678 | 0.03996004 | ENSMUSG00000033543;AL:9:70016923-70019389:70019486:70016923-70022437:70022737:+ |  |
| Csnk1d | AL | 0.1011180279 | 0.0412920413 | ENSMUSG00000025162;AL:11:120961749:120963906-120967995:120967612:120967736-120967995:- |  |
| Lrrk1 | AL | 0.3545547686 | 0.041958042 | ENSMUSG00000015133;AL:7:66226912:66226941-66261738:66258752:66260093-66261738:- |  |
| Vti1a | AL | 0.1509992031 | 0.0434565435 | ENSMUSG00000024983;AL:19:55499304-55576474:55577001:55499304-55623817:55626561:+ |  |
| Vti1a | AL | 0.2121773106 | 0.0434565435 | ENSMUSG00000024983;AL:19:55499304-55576474:55577001:55499304-55623817:55627309:+ |  |
| Plgrkt | AL | 0.2062619287 | 0.0437062937 | ENSMUSG00000016495;AL:19:29348677:29349005-29358317:29351128:29351174-29358317:- |  |
| Coa7 | AL | 0.4309841051 | 0.043956044 | ENSMUSG00000048351;AL:4:108332351-108332538:108332643:108332351-108338123:108341544:+ |  |
| Tpgs2 | AL | -0.1494926454 | 0.045954046 | ENSMUSG00000024269;AL:18:25127223:25129903-25140446:25136037:25139139-25140446:- |  |
| Eps15l1 | AL | 0.1694352052 | 0.0487012987 | ENSMUSG00000006276;AL:8:72341001:72341524-72367904:72345967:72346171-72367904:- |  |
| Eps15l1 | AL | -0.2669507482 | 0.0487012987 | ENSMUSG00000006276;AL:8:72345967:72346171-72367904:72358068:72358445-72367904:- |  |
| Fam220a | AL | 0.2813051916 | 0.0494505495 | ENSMUSG00000083012;AL:5:143549134-143551542:143551933:143549134-143562757:143564531:+ |  |
| Dguok | AL | 0.1765002188 | 0.04995005 | ENSMUSG00000014554;AL:6:83480217:83480436-83481347:83480865:83480942-83481347:- |  |
| Dguok | AL | 0.6386352452 | 0.04995005 | ENSMUSG00000014554;AL:6:83480309:83480436-83481347:83480865:83480942-83481347:- |  |
| Slc6a8 | A5 | -0.4735993714 | 0 | ENSMUSG00000019558;A5:X:73680071-73680132:73680047-73680132:+ |  |
| Wipi2 | A5 | -0.187415022 | 0 | ENSMUSG00000029578;A5:5:142666251-142666860:142666191-142666860:+ |  |
| Ovca2 | A5 | -0.5594670411 | 0 | ENSMUSG00000038268;A5:11:75178387-75178612:75178387-75178630:- |  |
| Chpt1 | A5 | 0.4782934599 | 0 | ENSMUSG00000060002;A5:10:88472984-88475333:88472984-88475426:- |  |
| Gtf2i | A5 | 0.1907101076 | 0 | ENSMUSG00000060261;A5:5:134295613-134314383:134295613-134314621:- |  |
| Rps12 | A5 | -0.2372527603 | 0 | ENSMUSG00000061983;A5:10:23786004-23786625:23786004-23786764:- |  |
| Cryab | A5 | -0.1668946244 | 0.001998002 | ENSMUSG00000032060;A5:9:50753095-50753186:50753084-50753186:+ |  |
| Slc6a8 | A5 | -0.2908146554 | 0.002997003 | ENSMUSG00000019558;A5:X:73680056-73680132:73680047-73680132:+ |  |
| Elovl7 | A5 | -0.2012173488 | 0.002997003 | ENSMUSG00000021696;A5:13:108267380-108270633:108267334-108270633:+ |  |
| E2f1 | A5 | -0.2598907368 | 0.002997003 | ENSMUSG00000027490;A5:2:154564508-154569067:154564508-154569489:- |  |
| Nudt21 | A5 | 0.2845282172 | 0.002997003 | ENSMUSG00000031754;A5:8:94032502-94036626:94032502-94036777:- |  |
| Rpl12 | A5 | -0.1574367707 | 0.002997003 | ENSMUSG00000038900;A5:2:32961960-32962049:32961839-32962049:+ |  |
| Fam149a | A5 | -0.2464440299 | 0.002997003 | ENSMUSG00000070044;A5:8:45341284-45342382:45341284-45342391:- |  |
| Gls | A5 | 0.1927480015 | 0.0034965035 | ENSMUSG00000026103;A5:1:52187794-52188679:52187794-52188685:- |  |
| Ptpn1 | A5 | -0.2354186977 | 0.0044955045 | ENSMUSG00000027540;A5:2:167932556-167963628:167932329-167963628:+ |  |
| Gtf3c6 | A5 | 0.2066986583 | 0.004995005 | ENSMUSG00000019837;A5:10:40257293-40257566:40257293-40257579:- |  |
| Sike1 | A5 | 0.2059269281 | 0.004995005 | ENSMUSG00000027854;A5:3:102997409-102998490:102997385-102998490:+ |  |
| Sbsn | A5 | 0.2869046649 | 0.004995005 | ENSMUSG00000046056;A5:7:30751915-30754809:30751699-30754809:+ |  |
| Hmga1 | A5 | -0.2241855618 | 0.004995005 | ENSMUSG00000046711;A5:17:27559703-27560928:27559670-27560928:+ |  |
| Wdr12 | A5 | 0.2539374703 | 0.0052447552 | ENSMUSG00000026019;A5:1:60097727-60097990:60097727-60098045:- |  |
| D17Wsu92e | A5 | 0.1221749793 | 0.0054945055 | ENSMUSG00000056692;A5:17:27753995-27767911:27753995-27768136:- |  |
| Gtpbp8 | A5 | -0.2444445095 | 0.005994006 | ENSMUSG00000022668;A5:16:44745493-44745999:44745493-44746200:- |  |
| Agap1 | A5 | -0.2804864441 | 0.005994006 | ENSMUSG00000055013;A5:1:89609599-89627761:89609583-89627761:+ |  |
| Sbsn | A5 | 0.3815245889 | 0.0062437562 | ENSMUSG00000046056;A5:7:30753469-30754809:30751699-30754809:+ |  |
| Hypk | A5 | -0.4469592935 | 0.006993007 | ENSMUSG00000027245;A5:2:121457701-121457992:121457291-121457992:+ |  |
| Mras | A5 | -0.172353408 | 0.0074925075 | ENSMUSG00000032470;A5:9:99411602-99436310:99411602-99436881:- |  |
| Srsf5 | A5 | -0.1400623163 | 0.0081585082 | ENSMUSG00000021134;A5:12:80945798-80946576:80945668-80946576:+ |  |
| Srsf5 | A5 | -0.1276698416 | 0.0081585082 | ENSMUSG00000021134;A5:12:80945798-80946576:80945672-80946576:+ |  |
| Ccdc80 | A5 | -0.2117926499 | 0.0087412587 | ENSMUSG00000022665;A5:16:45093668-45094029:45093597-45094029:+ |  |
| Cry1 | A5 | 0.1750326693 | 0.008991009 | ENSMUSG00000020038;A5:10:85148798-85151213:85148798-85151228:- |  |
| Pigp | A5 | -0.1914833598 | 0.008991009 | ENSMUSG00000022940;A5:16:94370192-94370366:94370192-94370605:- |  |
| Enpp4 | A5 | -0.2952116641 | 0.008991009 | ENSMUSG00000023961;A5:17:44102664-44105649:44102664-44105711:- |  |
| Hspb11 | A5 | -0.1349840436 | 0.008991009 | ENSMUSG00000063172;A5:4:107253995-107262467:107253962-107262467:+ |  |
| Faf2 | A5 | -0.1542251752 | 0.00999001 | ENSMUSG00000025873;A5:13:54641553-54645245:54641496-54645245:+ |  |
| Tpx2 | A5 | -0.1487031155 | 0.00999001 | ENSMUSG00000027469;A5:2:152848297-152854921:152848109-152854921:+ |  |
| Gatad2a | A5 | -0.1637576214 | 0.0104895105 | ENSMUSG00000036180;A5:8:69916448-69916560:69916448-69916569:- |  |
| Socs6 | A5 | -0.2246635275 | 0.0104895105 | ENSMUSG00000056153;A5:18:88870918-88887209:88870918-88887213:- |  |
| Dlk1 | A5 | -0.4493081301 | 0.010989011 | ENSMUSG00000040856;A5:12:109459892-109460118:109459832-109460118:+ |  |
| Rbm39 | A5 | 0.2722416718 | 0.0110603682 | ENSMUSG00000027620;A5:2:156177385-156177633:156177385-156177752:- |  |
| Mtch1 | A5 | -0.1500127237 | 0.011988012 | ENSMUSG00000024012;A5:17:29334005-29336167:29334005-29336218:- |  |
| Pqbp1 | A5 | 0.177946479 | 0.011988012 | ENSMUSG00000031157;A5:X:7898636-7898771:7898636-7898966:- |  |
| Chmp2a | A5 | 0.1663399176 | 0.011988012 | ENSMUSG00000033916;A5:7:13034042-13034502:13034042-13034635:- |  |
| St3gal5 | A5 | -0.1006600652 | 0.011988012 | ENSMUSG00000056091;A5:6:72097994-72128264:72097782-72128264:+ |  |
| 5730455P16Rik | A5 | 0.1420641993 | 0.0124875125 | ENSMUSG00000057181;A5:11:80376883-80377754:80376883-80377842:- |  |
| Unc13a | A5 | 0.5589970168 | 0.012987013 | ENSMUSG00000034799;A5:8:71662535-71662840:71662535-71662855:- |  |
| Armc6 | A5 | -0.1802906811 | 0.0134865135 | ENSMUSG00000002343;A5:8:70231406-70233946:70231406-70234110:- |  |
| Cstf1 | A5 | -0.1205215537 | 0.0134865135 | ENSMUSG00000027498;A5:2:172371027-172372929:172370856-172372929:+ |  |
| Skil | A5 | -0.1429692537 | 0.013986014 | ENSMUSG00000027660;A5:3:31113640-31116816:31113502-31116816:+ |  |
| Dph3 | A5 | -0.2141913809 | 0.014985015 | ENSMUSG00000021905;A5:14:32084981-32085410:32084981-32085552:- |  |
| Anp32e | A5 | -0.122676938 | 0.0157342657 | ENSMUSG00000015749;A5:3:95938039-95944307:95938003-95944307:+ |  |
| Hnrnpd | A5 | -0.3278168248 | 0.015984016 | ENSMUSG00000000568;A5:5:99961333-99962098:99961333-99962134:- |  |
| Zfp949 | A5 | -0.3713228279 | 0.015984016 | ENSMUSG00000032425;A5:9:88548508-88552716:88548300-88552716:+ |  |
| Dcaf15 | A5 | -0.1718013043 | 0.015984016 | ENSMUSG00000037103;A5:8:84097508-84097623:84097508-84097737:- |  |
| Tssc4 | A5 | -0.1504628811 | 0.015984016 | ENSMUSG00000045752;A5:7:143069850-143069936:143069820-143069936:+ |  |
| Akap13 | A5 | 0.257237098 | 0.015984016 | ENSMUSG00000066406;A5:7:75611560-75614921:75611548-75614921:+ |  |
| Cdk11b | A5 | -0.1096032609 | 0.0162337662 | ENSMUSG00000029062;A5:4:155625773-155626774:155625654-155626774:+ |  |
| Traf2 | A5 | 0.1463479833 | 0.016983017 | ENSMUSG00000026942;A5:2:25537149-25538845:25537149-25538866:- |  |
| Spry1 | A5 | 0.1580963541 | 0.016983017 | ENSMUSG00000037211;A5:3:37640105-37640520:37640064-37640520:+ |  |
| Luc7l | A5 | -0.3176716136 | 0.017982018 | ENSMUSG00000024188;A5:17:26254388-26255031:26254044-26255031:+ |  |
| C1s1 | A5 | -0.128285918 | 0.017982018 | ENSMUSG00000038521;A5:6:124541488-124542193:124541488-124542268:- |  |
| Selenoi | A5 | -0.126947374 | 0.0186480186 | ENSMUSG00000075703;A5:5:30257820-30263071:30257761-30263071:+ |  |
| Hemk1 | A5 | -0.3189818344 | 0.018981019 | ENSMUSG00000032579;A5:9:107329432-107330710:107329432-107330773:- |  |
| Rpl15 | A5 | 0.1268884858 | 0.0194805195 | ENSMUSG00000012405;A5:14:18270460-18270823:18270460-18270959:- |  |
| Golga3 | A5 | 0.174145067 | 0.0194805195 | ENSMUSG00000029502;A5:5:110184607-110185786:110184487-110185786:+ |  |
| Mau2 | A5 | 0.2440829377 | 0.01998002 | ENSMUSG00000031858;A5:8:70022669-70023607:70022669-70023614:- |  |
| Man1a | A5 | 0.1362332942 | 0.021978022 | ENSMUSG00000003746;A5:10:54075270-54075504:54075270-54075526:- |  |
| Ccnt2 | A5 | -0.1453189627 | 0.021978022 | ENSMUSG00000026349;A5:1:127798196-127799390:127797886-127799390:+ |  |
| Tmem176a | A5 | 0.1261130976 | 0.0224775225 | ENSMUSG00000023367;A5:6:48841710-48842204:48841589-48842204:+ |  |
| Rps27 | A5 | 0.1281977712 | 0.0224775225 | ENSMUSG00000090733;A5:3:90213301-90213381:90213301-90213610:- |  |
| Arpp19 | A5 | 0.1124983495 | 0.023976024 | ENSMUSG00000007656;A5:9:75037723-75037907:75037719-75037907:+ |  |
| Snrnp25 | A5 | -0.6095037525 | 0.023976024 | ENSMUSG00000040767;A5:11:32207050-32207574:32207043-32207574:+ |  |
| Efl1 | A5 | -0.1031860292 | 0.0244755245 | ENSMUSG00000038563;A5:7:82648678-82649526:82648649-82649526:+ |  |
| Pttg1 | A5 | -0.1706042416 | 0.024975025 | ENSMUSG00000020415;A5:11:43425681-43426174:43425681-43426189:- |  |
| Zmym5 | A5 | -0.1577165624 | 0.024975025 | ENSMUSG00000040123;A5:14:56796810-56797668:56796810-56797685:- |  |
| AU022252 | A5 | -0.2434386499 | 0.024975025 | ENSMUSG00000078584;A5:4:119232469-119232583:119232469-119232636:- |  |
| Nras | A5 | -0.3575988517 | 0.0258491508 | ENSMUSG00000027852;A5:3:103058830-103058905:103058629-103058905:+ |  |
| Smc5 | A5 | 0.1063700836 | 0.025974026 | ENSMUSG00000024943;A5:19:23214170-23214642:23214170-23214645:- |  |
| Vps72 | A5 | -0.2206951164 | 0.026973027 | ENSMUSG00000008958;A5:3:95111249-95118225:95111095-95118225:+ |  |
| Mul1 | A5 | 0.1689674821 | 0.026973027 | ENSMUSG00000041241;A5:4:138438408-138438951:138438362-138438951:+ |  |
| Lrrc14 | A5 | -0.2350713682 | 0.0274725275 | ENSMUSG00000033728;A5:15:76710927-76712867:76710836-76712867:+ |  |
| Zxdc | A5 | -0.1361349092 | 0.0274725275 | ENSMUSG00000034430;A5:6:90382520-90384168:90381900-90384168:+ |  |
| Dcun1d3 | A5 | -0.4141888114 | 0.0274725275 | ENSMUSG00000048787;A5:7:119859915-119861747:119859915-119861803:- |  |
| Btf3l4 | A5 | 0.1068774372 | 0.027972028 | ENSMUSG00000028568;A5:4:108830583-108833143:108830583-108833508:- |  |
| Nr2f2 | A5 | -0.1328403774 | 0.0284715285 | ENSMUSG00000030551;A5:7:70358290-70359814:70358290-70359888:- |  |
| Fbxo22 | A5 | 0.1459317269 | 0.0284715285 | ENSMUSG00000032309;A5:9:55209188-55209353:55209118-55209353:+ |  |
| Lamb2 | A5 | 0.1012147091 | 0.0284715285 | ENSMUSG00000052911;A5:9:108479974-108480058:108479969-108480058:+ |  |
| Mettl13 | A5 | -0.2264972647 | 0.02997003 | ENSMUSG00000026694;A5:1:162544385-162545768:162544385-162546246:- |  |
| Shroom3 | A5 | 0.3494803115 | 0.02997003 | ENSMUSG00000029381;A5:5:92962630-92964356:92962363-92964356:+ |  |
| Ankrd39 | A5 | -0.2219948739 | 0.02997003 | ENSMUSG00000079610;A5:1:36542865-36546910:36542865-36547086:- |  |
| Ankrd39 | A5 | 0.2254960603 | 0.0304695305 | ENSMUSG00000079610;A5:1:36539534-36541824:36539534-36541859:- |  |
| Lrrcc1 | A5 | -0.2290562039 | 0.0314685315 | ENSMUSG00000027550;A5:3:14540114-14545496:14540066-14545496:+ |  |
| Sbsn | A5 | -0.1292587424 | 0.0316350316 | ENSMUSG00000046056;A5:7:30753469-30754809:30751915-30754809:+ |  |
| Tlcd1 | A5 | -0.2244195742 | 0.031968032 | ENSMUSG00000019437;A5:11:78179221-78179370:78179056-78179370:+ |  |
| Washc1 | A5 | -0.1255641672 | 0.0324675325 | ENSMUSG00000024101;A5:17:66111731-66113863:66111649-66113863:+ |  |
| Lrrc14 | A5 | -0.2501012128 | 0.032967033 | ENSMUSG00000033728;A5:15:76711530-76712867:76710836-76712867:+ |  |
| Taf9 | A5 | -0.2838170718 | 0.032967033 | ENSMUSG00000052293;A5:13:100651828-100654191:100651786-100654191:+ |  |
| Ube2m | A5 | 0.1385175083 | 0.033966034 | ENSMUSG00000005575;A5:7:13036660-13037555:13036660-13037595:- |  |
| Steap3 | A5 | -0.2063975346 | 0.0346320346 | ENSMUSG00000026389;A5:1:120244267-120269983:120244267-120270125:- |  |
| Sipa1l1 | A5 | 0.1476080223 | 0.0352147852 | ENSMUSG00000042700;A5:12:82449404-82449901:82449391-82449901:+ |  |
| Taf1c | A5 | -0.1674364003 | 0.0354645355 | ENSMUSG00000031832;A5:8:119601780-119602758:119601780-119602831:- |  |
| Smarcb1 | A5 | -0.1106218885 | 0.035964036 | ENSMUSG00000000902;A5:10:75915193-75916773:75915193-75916800:- |  |
| Cul3 | A5 | -0.1034535396 | 0.035964036 | ENSMUSG00000004364;A5:1:80304193-80322859:80304193-80322987:- |  |
| P2ry2 | A5 | -0.3267894143 | 0.036963037 | ENSMUSG00000032860;A5:7:101003754-101011712:101003754-101011930:- |  |
| Ldlrad3 | A5 | 0.1507357781 | 0.036963037 | ENSMUSG00000048058;A5:2:102070058-102113533:102070058-102113629:- |  |
| Emc8 | A5 | -0.1050868549 | 0.0374625375 | ENSMUSG00000031819;A5:8:120658248-120658528:120658248-120658552:- |  |
| Hccs | A5 | -0.1039730779 | 0.037962038 | ENSMUSG00000031352;A5:X:169319590-169319976:169319590-169320115:- |  |
| Setd6 | A5 | -0.2069065711 | 0.037962038 | ENSMUSG00000031671;A5:8:95717062-95717921:95717041-95717921:+ |  |
| Impdh2 | A5 | -0.1945333962 | 0.037962038 | ENSMUSG00000062867;A5:9:108561857-108562174:108561698-108562174:+ |  |
| Smarcc2 | A5 | 0.1170196487 | 0.0384615385 | ENSMUSG00000025369;A5:10:128488359-128488896:128488017-128488896:+ |  |
| Tshz1 | A5 | -0.1213269332 | 0.0384615385 | ENSMUSG00000046982;A5:18:84016241-84085364:84016241-84086015:- |  |
| Brd8 | A5 | 0.2591586087 | 0.038961039 | ENSMUSG00000003778;A5:18:34608055-34608418:34608055-34609805:- |  |
| Oaz2 | A5 | 0.1234122126 | 0.038961039 | ENSMUSG00000040652;A5:9:65686271-65686273:65686268-65686273:+ |  |
| Mlx | A5 | -0.1914340417 | 0.0412087912 | ENSMUSG00000017801;A5:11:101087529-101087754:101087314-101087754:+ |  |
| Mlx | A5 | -0.1179572738 | 0.0412087912 | ENSMUSG00000017801;A5:11:101087529-101087754:101087367-101087754:+ |  |
| Cd151 | A5 | -0.1090891409 | 0.041958042 | ENSMUSG00000025510;A5:7:141467584-141468419:141467466-141468419:+ |  |
| Mynn | A5 | -0.1140475323 | 0.041958042 | ENSMUSG00000037730;A5:3:30603800-30607037:30603670-30607037:+ |  |
| Sirpa | A5 | 0.1826846889 | 0.041958042 | ENSMUSG00000037902;A5:2:129593075-129593604:129593004-129593604:+ |  |
| Cenpa | A5 | -0.2952968354 | 0.0427072927 | ENSMUSG00000029177;A5:5:30673443-30674038:30673059-30674038:+ |  |
| Uvssa | A5 | 0.226435476 | 0.044955045 | ENSMUSG00000037355;A5:5:33379002-33379226:33378917-33379226:+ |  |
| Rhno1 | A5 | -0.131271378 | 0.044955045 | ENSMUSG00000048668;A5:6:128359354-128362368:128359354-128362839:- |  |
| Cdan1 | A5 | -0.2424484912 | 0.0464535465 | ENSMUSG00000027284;A5:2:120720989-120722610:120720989-120722657:- |  |
| Pigg | A5 | -0.162824411 | 0.046953047 | ENSMUSG00000029263;A5:5:108332192-108332684:108332168-108332684:+ |  |
| Luc7l | A5 | -0.1172312469 | 0.047952048 | ENSMUSG00000024188;A5:17:26254388-26255031:26253091-26255031:+ |  |
| Mtch2 | A5 | -0.1191687256 | 0.048951049 | ENSMUSG00000027282;A5:2:90847385-90849371:90847363-90849371:+ |  |
| Rac3 | A5 | 0.1738867847 | 0.04995005 | ENSMUSG00000018012;A5:11:120721641-120722231:120721620-120722231:+ |  |
| Nipa2 | A5 | -0.1440813131 | 0.04995005 | ENSMUSG00000030452;A5:7:55961572-55962200:55961572-55962396:- |  |
| Arfip2 | A5 | -0.163739221 | 0.04995005 | ENSMUSG00000030881;A5:7:105637278-105637863:105637278-105637940:- |  |
| Mdc1 | A5 | 0.1114033061 | 0.04995005 | ENSMUSG00000061607;A5:17:35841765-35844432:35841679-35844432:+ |  |
| Zwilch | A3 | -0.7324736817 | 0 | ENSMUSG00000032400;A3:9:64162763-64165363:64162759-64165363:- |  |
| Pdcd6ip | A3 | 0.2026773277 | 0 | ENSMUSG00000032504;A3:9:113685438-113687625:113685423-113687625:- |  |
| Dlk1 | A3 | 0.5700730105 | 0 | ENSMUSG00000040856;A3:12:109459892-109460043:109459892-109460118:+ |  |
| Armcx3 | A3 | 0.2781186187 | 0 | ENSMUSG00000049047;A3:X:134756730-134757236:134756730-134757264:+ |  |
| Sdad1 | A3 | -0.255624949 | 0.0014985015 | ENSMUSG00000029415;A3:5:92298261-92300062:92298258-92300062:- |  |
| Fbxo28 | A3 | -0.3051235588 | 0.0014985015 | ENSMUSG00000047539;A3:1:182318005-182326254:182317958-182326254:- |  |
| Col12a1 | A3 | 0.1808942544 | 0.001998002 | ENSMUSG00000032332;A3:9:79601179-79602200:79600007-79602200:- |  |
| Hnrnph1 | A3 | -0.2602799017 | 0.003996004 | ENSMUSG00000007850;A3:11:50383314-50383680:50383314-50383831:+ |  |
| Zkscan1 | A3 | -0.2523268123 | 0.003996004 | ENSMUSG00000029729;A3:5:138085231-138092920:138085231-138092926:+ |  |
| Gspt1 | A3 | 0.1664226922 | 0.003996004 | ENSMUSG00000062203;A3:16:11239099-11240666:11239096-11240666:- |  |
| Sec22b | A3 | -0.1216512648 | 0.0044955045 | ENSMUSG00000027879;A3:3:97901420-97907221:97901420-97907225:+ |  |
| Fau | A3 | 0.1579400292 | 0.0044955045 | ENSMUSG00000038274;A3:19:6058001-6058243:6058001-6058246:+ |  |
| Helz | A3 | 0.2883749967 | 0.004995005 | ENSMUSG00000020721;A3:11:107626714-107627350:107626714-107627353:+ |  |
| Zranb2 | A3 | -0.3457992728 | 0.004995005 | ENSMUSG00000028180;A3:3:157536386-157536448:157536386-157536483:+ |  |
| Ptpn1 | A3 | 0.1614918707 | 0.0067432567 | ENSMUSG00000027540;A3:2:167965110-167967697:167965110-167967729:+ |  |
| Hypk | A3 | -0.4753574568 | 0.006993007 | ENSMUSG00000027245;A3:2:121457291-121457646:121457291-121457992:+ |  |
| Ppil2 | A3 | 0.1851317913 | 0.0074925075 | ENSMUSG00000022771;A3:16:17087213-17088745:17087115-17088745:- |  |
| Gnptg | A3 | -0.1626665103 | 0.0074925075 | ENSMUSG00000035521;A3:17:25235480-25235534:25235459-25235534:- |  |
| Aup1 | A3 | -0.1997162423 | 0.0074925075 | ENSMUSG00000068328;A3:6:83055048-83055129:83055048-83055506:+ |  |
| Nolc1 | A3 | 0.1478107357 | 0.007992008 | ENSMUSG00000015176;A3:19:46078758-46078873:46078758-46078876:+ |  |
| Nolc1 | A3 | 0.1573299954 | 0.007992008 | ENSMUSG00000015176;A3:19:46079258-46079908:46079258-46079911:+ |  |
| Ppp2r2a | A3 | -0.2687162529 | 0.007992008 | ENSMUSG00000022052;A3:14:67016653-67019716:67015258-67019716:- |  |
| Bclaf1 | A3 | -0.1873149696 | 0.007992008 | ENSMUSG00000037608;A3:10:20322116-20322963:20322116-20322969:+ |  |
| Rwdd4a | A3 | -0.1685521825 | 0.008991009 | ENSMUSG00000031568;A3:8:47537359-47542692:47537359-47542696:+ |  |
| Ankrd40 | A3 | -0.1664921669 | 0.00999001 | ENSMUSG00000020864;A3:11:94338521-94339553:94338521-94339633:+ |  |
| Zranb2 | A3 | -0.1129314824 | 0.00999001 | ENSMUSG00000028180;A3:3:157536386-157536478:157536386-157536483:+ |  |
| Rps11 | A3 | 0.133940149 | 0.010989011 | ENSMUSG00000003429;A3:7:45122558-45122829:45122548-45122829:- |  |
| Mad2l2 | A3 | -0.162099722 | 0.010989011 | ENSMUSG00000029003;A3:4:148144727-148145104:148144727-148145126:+ |  |
| Dlk1 | A3 | 0.3662612726 | 0.010989011 | ENSMUSG00000040856;A3:12:109459892-109460043:109459892-109460046:+ |  |
| Ino80d | A3 | -0.2895935034 | 0.0114885115 | ENSMUSG00000040865;A3:1:63093531-63093640:63093440-63093640:- |  |
| Rbmx | A3 | 0.1976513007 | 0.011988012 | ENSMUSG00000031134;A3:X:57391701-57392852:57391698-57392852:- |  |
| Zranb2 | A3 | -0.1323526705 | 0.0124875125 | ENSMUSG00000028180;A3:3:157536386-157536448:157536386-157536478:+ |  |
| Slc25a36 | A3 | -0.3968870703 | 0.0124875125 | ENSMUSG00000032449;A3:9:97092605-97093077:97090174-97093077:- |  |
| D030056L22Rik | A3 | 0.1000175402 | 0.0124875125 | ENSMUSG00000047044;A3:19:18713659-18717219:18713659-18717222:+ |  |
| Cast | A3 | -0.122906392 | 0.012987013 | ENSMUSG00000021585;A3:13:74704205-74713646:74704202-74713646:- |  |
| Tmem209 | A3 | 0.2203399717 | 0.012987013 | ENSMUSG00000029782;A3:6:30508842-30509685:30508802-30509685:- |  |
| Gemin5 | A3 | -0.1765169928 | 0.012987013 | ENSMUSG00000037275;A3:11:58122378-58124932:58122375-58124932:- |  |
| Rc3h1 | A3 | -0.1585594192 | 0.0134865135 | ENSMUSG00000040423;A3:1:160963840-160964950:160963840-160964977:+ |  |
| Ccdc93 | A3 | -0.2059454396 | 0.013986014 | ENSMUSG00000026339;A3:1:121461979-121462233:121461979-121462236:+ |  |
| Cdc42se1 | A3 | -0.4994404444 | 0.0142357642 | ENSMUSG00000046722;A3:3:95232328-95232448:95232328-95232549:+ |  |
| Psip1 | A3 | -0.1447901086 | 0.014985015 | ENSMUSG00000028484;A3:4:83462553-83463595:83462473-83463595:- |  |
| Tial1 | A3 | -0.1609613608 | 0.014985015 | ENSMUSG00000030846;A3:7:128448770-128454936:128448719-128454936:- |  |
| Casp4 | A3 | 0.1645702707 | 0.014985015 | ENSMUSG00000033538;A3:9:5308914-5321258:5308914-5321426:+ |  |
| Smurf1 | A3 | -0.1269832697 | 0.014985015 | ENSMUSG00000038780;A3:5:144880709-144881618:144880700-144881618:- |  |
| Rbfox2 | A3 | 0.1074044429 | 0.015984016 | ENSMUSG00000033565;A3:15:77098084-77099240:77098072-77099240:- |  |
| Prkag2 | A3 | 0.1401233128 | 0.016983017 | ENSMUSG00000028944;A3:5:24889188-24908323:24889185-24908323:- |  |
| Mrpl3 | A3 | -0.1940941523 | 0.0174825175 | ENSMUSG00000032563;A3:9:105053512-105054421:105053512-105054425:+ |  |
| Mrpl3 | A3 | 0.206924051 | 0.0174825175 | ENSMUSG00000032563;A3:9:105053512-105054425:105053512-105054445:+ |  |
| Stk38l | A3 | 0.1382751047 | 0.017982018 | ENSMUSG00000001630;A3:6:146775491-146775573:146775491-146775594:+ |  |
| Surf1 | A3 | -0.418255291 | 0.017982018 | ENSMUSG00000015790;A3:2:26915769-26915955:26915749-26915955:- |  |
| Wdr26 | A3 | 0.13618032 | 0.017982018 | ENSMUSG00000038733;A3:1:181203224-181209051:181203160-181209051:- |  |
| Xlr | A3 | -0.1611874123 | 0.017982018 | ENSMUSG00000054626;A3:X:53796268-53797632:53796220-53797632:- |  |
| Cdv3 | A3 | -0.1318433303 | 0.0187312687 | ENSMUSG00000032803;A3:9:103356029-103356280:103355313-103356280:- |  |
| Ncoa3 | A3 | -0.1988971922 | 0.018981019 | ENSMUSG00000027678;A3:2:166067645-166068329:166067645-166068332:+ |  |
| Skap2 | A3 | -0.100372442 | 0.018981019 | ENSMUSG00000059182;A3:6:52003755-52012335:52003734-52012335:- |  |
| Dlk1 | A3 | 0.1020849644 | 0.0192307692 | ENSMUSG00000040856;A3:12:109459892-109460112:109459892-109460118:+ |  |
| Prelid3b | A3 | -0.250943244 | 0.0194805195 | ENSMUSG00000016257;A3:2:174468399-174472832:174468387-174472832:- |  |
| 1600012H06Rik | A3 | -0.1976853734 | 0.0194805195 | ENSMUSG00000050088;A3:17:14943319-14943567:14943319-14943572:+ |  |
| Lrrc40 | A3 | -0.3005166905 | 0.01998002 | ENSMUSG00000063052;A3:3:158040622-158041586:158040622-158041620:+ |  |
| Snx13 | A3 | 0.13326457 | 0.020979021 | ENSMUSG00000020590;A3:12:35112504-35119733:35112504-35119766:+ |  |
| Nfx1 | A3 | 0.1228801362 | 0.020979021 | ENSMUSG00000028423;A3:4:41009269-41011527:41009269-41012058:+ |  |
| 1600012H06Rik | A3 | -0.1459326322 | 0.020979021 | ENSMUSG00000050088;A3:17:14943319-14943502:14943319-14943572:+ |  |
| Kcnj15 | A3 | -0.4891657753 | 0.0212287712 | ENSMUSG00000062609;A3:16:95257734-95258202:95257734-95258247:+ |  |
| Luc7l | A3 | -0.1755720958 | 0.021978022 | ENSMUSG00000024188;A3:17:26253091-26253974:26253091-26255031:+ |  |
| Hspbp1 | A3 | 0.1488312041 | 0.021978022 | ENSMUSG00000063802;A3:7:4684566-4684894:4684482-4684894:- |  |
| Hspbp1 | A3 | 0.1310798475 | 0.021978022 | ENSMUSG00000063802;A3:7:4684569-4684894:4684482-4684894:- |  |
| Surf1 | A3 | 0.1592410997 | 0.023976024 | ENSMUSG00000015790;A3:2:26915749-26915955:26915729-26915955:- |  |
| Surf1 | A3 | -0.1819105648 | 0.023976024 | ENSMUSG00000015790;A3:2:26915769-26915955:26915729-26915955:- |  |
| Foxp1 | A3 | 0.1460425295 | 0.023976024 | ENSMUSG00000030067;A3:6:99010057-99015424:99010054-99015424:- |  |
| Spast | A3 | -0.1284939997 | 0.024975025 | ENSMUSG00000024068;A3:17:74339445-74351950:74339445-74351953:+ |  |
| Ehbp1 | A3 | 0.1544875305 | 0.025974026 | ENSMUSG00000042302;A3:11:22146700-22151067:22146625-22151067:- |  |
| Pcnx | A3 | -0.1374351043 | 0.0262237762 | ENSMUSG00000021140;A3:12:81919380-81928242:81919380-81928245:+ |  |
| Pcnx | A3 | -0.1723875585 | 0.0262237762 | ENSMUSG00000021140;A3:12:81948415-81950101:81948415-81950116:+ |  |
| Fam89b | A3 | -0.1416737691 | 0.026973027 | ENSMUSG00000024939;A3:19:5728878-5729237:5728829-5729237:- |  |
| Ssr2 | A3 | -0.2011747136 | 0.026973027 | ENSMUSG00000041355;A3:3:88581038-88583737:88581038-88583800:+ |  |
| Wdr73 | A3 | -0.163083036 | 0.027972028 | ENSMUSG00000025722;A3:7:80900453-80900644:80900436-80900644:- |  |
| Kcnj15 | A3 | 0.4525505015 | 0.0283050283 | ENSMUSG00000062609;A3:16:95257734-95258247:95257734-95258351:+ |  |
| Usp48 | A3 | -0.1669861903 | 0.02997003 | ENSMUSG00000043411;A3:4:137623388-137625157:137623388-137625160:+ |  |
| Rbmx | A3 | -0.1490078937 | 0.030969031 | ENSMUSG00000031134;A3:X:57391698-57392852:57391682-57392852:- |  |
| Psme1 | A3 | -0.5651287523 | 0.031968032 | ENSMUSG00000022216;A3:14:55580427-55580595:55580427-55580744:+ |  |
| Mterf2 | A3 | -0.1763560214 | 0.032967033 | ENSMUSG00000049038;A3:10:85126486-85127962:85126465-85127962:- |  |
| 0610010K14Rik | A3 | 0.1985544073 | 0.0346320346 | ENSMUSG00000020831;A3:11:70235468-70235696:70235464-70235696:- |  |
| Eif4a2 | A3 | -0.1241100276 | 0.034965035 | ENSMUSG00000022884;A3:16:23107820-23108585:23107820-23108588:+ |  |
| Wdr4 | A3 | 0.4160910697 | 0.034965035 | ENSMUSG00000024037;A3:17:31503727-31509821:31503666-31509821:- |  |
| Fam220a | A3 | -0.2333175758 | 0.035964036 | ENSMUSG00000083012;A3:5:143549134-143551409:143549134-143551542:+ |  |
| Tm7sf2 | A3 | -0.2108549836 | 0.0374625375 | ENSMUSG00000024799;A3:19:6063101-6063324:6063073-6063324:- |  |
| Baz1a | A3 | -0.4355830039 | 0.0374625375 | ENSMUSG00000035021;A3:12:54927713-54929527:54927624-54929527:- |  |
| Socs6 | A3 | 0.1587067502 | 0.0384615385 | ENSMUSG00000056153;A3:18:88887065-88887209:88887052-88887209:- |  |
| Apoa2 | A3 | 0.3003964115 | 0.038961039 | ENSMUSG00000005681;A3:1:171225121-171225284:171225121-171225299:+ |  |
| Cbx5 | A3 | -0.1333131921 | 0.038961039 | ENSMUSG00000009575;A3:15:103215125-103215268:103215121-103215268:- |  |
| Rps12 | A3 | -0.197021996 | 0.0391275391 | ENSMUSG00000061983;A3:10:23787044-23787156:23786880-23787156:- |  |
| Fam135a | A3 | -0.460968953 | 0.03996004 | ENSMUSG00000026153;A3:1:24026823-24028200:24026711-24028200:- |  |
| Rngtt | A3 | -0.107550677 | 0.03996004 | ENSMUSG00000028274;A3:4:33499074-33500238:33499074-33500299:+ |  |
| Aen | A3 | -0.1194904999 | 0.03996004 | ENSMUSG00000030609;A3:7:78896014-78902230:78896014-78902344:+ |  |
| Trp53rkb | A3 | 0.297386822 | 0.0404595405 | ENSMUSG00000042854;A3:2:166794068-166795126:166794068-166795382:+ |  |
| Arfip2 | A3 | 0.1313895908 | 0.0412087912 | ENSMUSG00000030881;A3:7:105638344-105639009:105638327-105639009:- |  |
| Snrnp48 | A3 | -0.1035666498 | 0.0414585415 | ENSMUSG00000021431;A3:13:38211141-38214605:38211141-38216325:+ |  |
| Serpinb8 | A3 | -0.1160863048 | 0.0414585415 | ENSMUSG00000026315;A3:1:107605936-107606921:107605936-107607010:+ |  |
| Trub1 | A3 | -0.1642164434 | 0.0424575425 | ENSMUSG00000025086;A3:19:57485212-57485352:57485212-57487265:+ |  |
| Syn1 | A3 | -0.3493896032 | 0.0424575425 | ENSMUSG00000037217;A3:X:20861609-20862499:20861571-20862499:- |  |
| Wdr73 | A3 | -0.134531533 | 0.042957043 | ENSMUSG00000025722;A3:7:80893341-80893649:80893266-80893649:- |  |
| Cd2bp2 | A3 | -0.1884692136 | 0.043956044 | ENSMUSG00000042502;A3:7:127195467-127195915:127195461-127195915:- |  |
| Arhgef40 | A3 | -0.1115348805 | 0.044955045 | ENSMUSG00000004562;A3:14:52004742-52004879:52004742-52004989:+ |  |
| Dusp12 | A3 | -0.1434059988 | 0.044955045 | ENSMUSG00000026659;A3:1:170881041-170885067:170881028-170885067:- |  |
| Stoml2 | A3 | 0.1217033877 | 0.044955045 | ENSMUSG00000028455;A3:4:43031129-43031329:43031108-43031329:- |  |
| Etv6 | A3 | -0.1398086137 | 0.044955045 | ENSMUSG00000030199;A3:6:134036138-134137285:134036138-134137288:+ |  |
| Nkiras1 | A3 | -0.425507783 | 0.0457042957 | ENSMUSG00000021772;A3:14:18271323-18276696:18271323-18276826:+ |  |
| Snx15 | A3 | -0.2616852007 | 0.045954046 | ENSMUSG00000024787;A3:19:6124188-6128049:6123950-6128049:- |  |
| Nmb | A3 | -0.2804835275 | 0.0474525475 | ENSMUSG00000025723;A3:7:80902503-80904134:80902498-80904134:- |  |
| Usp3 | A3 | 0.1032031119 | 0.0474525475 | ENSMUSG00000032376;A3:9:66542626-66543946:66542613-66543946:- |  |
| Atf6b | A3 | 0.1102469296 | 0.047952048 | ENSMUSG00000015461;A3:17:34647739-34648219:34647739-34648230:+ |  |
| Pif1 | A3 | -0.2262579064 | 0.047952048 | ENSMUSG00000041064;A3:9:65590054-65591687:65590054-65591720:+ |  |
| Supt20 | A3 | -0.1716597011 | 0.048951049 | ENSMUSG00000027751;A3:3:54715689-54716638:54715689-54717576:+ |  |
| Midn | A3 | -0.1058732488 | 0.048951049 | ENSMUSG00000035621;A3:10:80151723-80153517:80151723-80153591:+ |  |
| Trip12 | A3 | -0.1040741974 | 0.0494505495 | ENSMUSG00000026219;A3:1:84759127-84760763:84759046-84760763:- |  |
| Trip12 | A3 | -0.1040741974 | 0.0494505495 | ENSMUSG00000026219;A3:1:84777507-84782827:84777489-84782827:- |  |

**Supplementary Table S2**

**List of 299 genes with splicing events significantly changed between control and *Snai1^-/-^***. RNA samples from Control and *Snai1^-/-^* MEFs treated with 5ng/mL TGFβ for 3 h were paired-end sequenced in CRG Sequencing Unit until reaching 80M reads/sample. The SANJUAN software was run with a threshold of 0.15 delta Percentage Spliced In. Significant events are shown in the list.

| **Gene_Name(s)** | **COMPET_TYPE** | **High_Confidence_Junction** | **Competing_Junction** |
| --- | --- | --- | --- |
| Mapk12 | COMP_5'SS_2789_CE | chr15_89133150_89137400_- | chr15_89133150_89134611_- |
| Slc22a17 | COMP_5'SS_616 | chr14_54907630_54908501_- | chr14_54907630_54907885_- |
| Grk6 | COMP_3'SS_-2 | chr13_55459147_55459781_+ | chr13_55459147_55459783_+ |
| Tpx2 | COMP_5'SS_-188 | chr2_152848109_152854920_+ | chr2_152848297_152854920_+ |
| Lama2 | COMP_5'SS_-1811_CE | chr10_27018527_27021460_- | chr10_27018527_27023271_- |
| Fam19a5 | COMP_5'SS_29611_CE | chr15_87655097_87681479_+ | chr15_87625486_87681479_+ |
| BC037034 | COMP_5'SS_-249_CE/RET_INTRON | chr5_138260989_138261552_- | chr5_138260989_138261801_- |
| Gale | COMP_3'SS_-116 | chr4_135966116_135966253_+ | chr4_135966116_135966369_+ |
| Ap1g1 | COMP_5'SS_946_CE | chr8_109830621_109832716_+ | chr8_109829675_109832716_+ |
| Med23 | COMP_5'SS_572_CE | chr10_24883221_24888399_+ | chr10_24882649_24888399_+ |
| Adk | COMP_3'SS_-38645_CE | chr14_21052700_21053721_+ | chr14_21052700_21092366_+ |
| Ppp2cb | nonME_COMP/RET_INTRON | chr8_33610864_33611747_+ | chr8_33611921_33615449_+ |
| Huwe1 | COMP_5'SS_1775_CE | chrX_151859688_151860166_+ | chrX_151857913_151860166_+ |
| Ndufa9 | nonME_COMP/RET_INTRON | chr6_126836321_126840497_- | chr6_126834496_126836218_- |
| Rsrp1 | COMP_5'SS_389_CE | chr4_134925821_134926734_+ | chr4_134925432_134926734_+ |
| Wbp1l | COMP_3'SS_-37641_CE | chr19_46599293_46606711_+ | chr19_46599293_46644352_+ |
| Srebf2 | nonME_COMP/RET_INTRON | chr15_82199820_82203184_+ | chr15_82203296_82203696_+ |
| Cast | COMP_5'SS_-9957_CE | chr13_74754370_74770545_- | chr13_74754370_74780502_- |
| Ilf3 | COMP_3'SS_-412_CE | chr9_21387797_21388111_+ | chr9_21387797_21388523_+ |
| 2010111I01Rik | DUAL_COMP | chr13_63298744_63298901_+ | chr13_63298856_63301778_+ |
| Gatad2a | COMP_3'SS_369_CE/RET_INTRON | chr8_69912238_69912922_- | chr8_69911869_69912922_- |
| Dnajc18 | COMP_5'SS_-614_CE | chr18_35700990_35702411_- | chr18_35700990_35703025_- |
| Serpinh1 | DUAL_COMP | chr7_99349455_99353052_- | chr7_99349464_99352965_- |
| Golga2 | COMP_3'SS_-846_CE | chr2_32297577_32297753_+ | chr2_32297577_32298599_+ |
| Nav3 | COMP_5'SS_891 | chr10_109718366_109719956_- | chr10_109718366_109719065_- |
| Rsrc2 | COMP_3'SS_-1281_CE | chr5_123745589_123749288_- | chr5_123746870_123749288_- |
| Dtnb | COMP_5'SS_2209_CE | chr12_3754116_3772586_+ | chr12_3751907_3772586_+ |
| Adgrg2 | COMP_5'SS_1253_CE | chrX_160492752_160496304_+ | chrX_160491499_160496304_+ |
| Akap7 | COMP_3'SS_846 | chr10_25171248_25220610_- | chr10_25170402_25220610_- |
| Asph | COMP_3'SS_479_CE | chr4_9598780_9601306_- | chr4_9598301_9601306_- |
| Eif4a2 | COMP_5'SS_565 | chr16_23112461_23113166_+ | chr16_23111896_23113166_+ |
| Med23 | COMP_3'SS_-5196_CE | chr10_24882649_24883203_+ | chr10_24882649_24888399_+ |
| P4ha2 | COMP_3'SS_-108 | chr11_54125816_54126237_+ | chr11_54125816_54126345_+ |
| Clstn1 | COMP_5'SS_11944_CE | chr4_149626116_149627211_+ | chr4_149614172_149627211_+ |
| Rbfox2 | COMP_5'SS_11952_CE | chr15_77231437_77257441_- | chr15_77231437_77245489_- |
| Abi3bp | COMP_5'SS_7527_CE | chr16_56613720_56642488_+ | chr16_56606193_56642488_+ |
| Jak3 | COMP_5'SS_-571_CE | chr8_71682413_71683331_+ | chr8_71682984_71683331_+ |
| Adgrg6 | COMP_3'SS_5557_CE | chr10_14456221_14458079_- | chr10_14450664_14458079_- |
| Rbfox2 | COMP_3'SS_-2636_CE | chr15_77091854_77097939_- | chr15_77094490_77097939_- |
| Rrbp1 | COMP_3'SS_15657_CE | chr2_143990266_144010998_- | chr2_143974609_144010998_- |
| Rps24 | COMP_5'SS_1956_CE | chr14_24495449_24495774_+ | chr14_24493493_24495774_+ |
| Agbl3 | COMP_5'SS_11 | chr6_34798332_34799132_+ | chr6_34798321_34799132_+ |
| Rbfox2 | COMP_3'SS_8335_CE | chr15_77094490_77097939_- | chr15_77086155_77097939_- |
| Fance | DUAL_COMP | chr17_28320856_28326151_+ | chr17_28322771_28326093_+ |
| Tmem161b | COMP_5'SS_60 | chr13_84284185_84286684_+ | chr13_84284125_84286684_+ |
| Dst | COMP_5'SS_9657_CE | chr1_34238903_34242618_+ | chr1_34229246_34242618_+ |
| Card19 | COMP_5'SS_-4075_CE | chr13_49203789_49203928_- | chr13_49203789_49208003_- |
| A630095E13Rik | COMP_5'SS_83 | chr9_36635984_36637286_- | chr9_36635984_36637203_- |
| Odf2 | COMP_5'SS_57 | chr2_29893569_29901076_+ | chr2_29893512_29901076_+ |
| Ankib1 | COMP_3'SS_18 | chr5_3694893_3700355_- | chr5_3694875_3700355_- |
| Mtmr2 | COMP_5'SS_11088_CE/RET_INTRON | chr9_13760488_13782276_+ | chr9_13749400_13782276_+ |
| Idh3g | COMP_3'SS_206 | chrX_73782309_73782674_- | chrX_73782103_73782674_- |
| BC023829 | COMP_3'SS_-3091_CE | chrX_70467203_70476977_- | chrX_70470294_70476977_- |
| Bcl2l12 | COMP_5'SS_552_CE | chr7_44993031_44994362_- | chr7_44993031_44993810_- |
| Eps15 | COMP_5'SS_108 | chr4_109321325_109322069_+ | chr4_109321217_109322069_+ |
| Bdh2 | COMP_5'SS_2092_CE | chr3_135299025_135300673_+ | chr3_135296933_135300673_+ |
| Sytl2 | COMP_3'SS_1311_CE | chr7_90392453_90396473_+ | chr7_90392453_90395162_+ |
| Acly | COMP_5'SS_-1043_CE | chr11_100498762_100503159_- | chr11_100498762_100504202_- |
| Slc7a6os | COMP_3'SS_8 | chr8_106202359_106204330_- | chr8_106202351_106204330_- |
| Mdm4 | COMP_3'SS_-831_CE | chr1_133003890_133009136_- | chr1_133004721_133009136_- |
| Pitpnb | COMP_5'SS_2494_CE/RET_INTRON | chr5_111385604_111386502_+ | chr5_111383110_111386502_+ |
| Trub2 | COMP_5'SS_-1641_CE/RET_INTRON | chr2_29782201_29783311_- | chr2_29782201_29784952_- |
| Baiap2 | COMP_5'SS_-2499_CE | chr11_120000586_120006358_+ | chr11_120003085_120006358_+ |
| Atxn2 | COMP_5'SS_1941_CE | chr5_121813553_121814337_+ | chr5_121811612_121814337_+ |
| Sh3pxd2a | COMP_5'SS_23164_CE | chr19_47314166_47343439_- | chr19_47314166_47320275_- |
| Ppt1 | UNIDNT_COMP/RET_INTRON | chr4_122848535_122853590_+ | NA |
| Nrg1 | COMP_5'SS_4517_CE | chr8_31826355_31837669_- | chr8_31826355_31833152_- |
| Kdm2b | COMP_5'SS_327 | chr5_122881058_122881963_- | chr5_122881058_122881636_- |
| Ilf3 | COMP_5'SS_-353_CE | chr9_21387797_21388523_+ | chr9_21388150_21388523_+ |
| S100a6 | COMP_3'SS_8493_CE | chr3_90614373_90623726_+ | chr3_90614373_90615233_+ |
| Tpm2 | COMP_5'SS_-359_CE | chr4_43518464_43518643_- | chr4_43518464_43519002_- |
| Fhl3 | COMP_5'SS_3080_CE | chr4_124703931_124705612_+ | chr4_124700851_124705612_+ |
| Ubr4 | COMP_3'SS_-1138_CE | chr4_139431761_139432481_+ | chr4_139431761_139433619_+ |
| Golga2 | COMP_5'SS_257_CE/RET_INTRON | chr2_32297834_32298599_+ | chr2_32297577_32298599_+ |
| Arid5b | COMP_5'SS_49540_CE | chr10_68135106_68186025_- | chr10_68135106_68136485_- |
| Aplp2 | COMP_3'SS_3055_CE/RET_INTRON | chr9_31167728_31168707_- | chr9_31164673_31168707_- |
| Mtmr2 | COMP_3'SS_-21859_CE | chr9_13749400_13760417_+ | chr9_13749400_13782276_+ |
| Flnc | COMP_3'SS_-400/RET_INTRON | chr6_29452380_29452798_+ | chr6_29452380_29453198_+ |
| Hspbp1 | COMP_3'SS_-84 | chr7_4684482_4684893_- | chr7_4684566_4684893_- |
| Aplp2 | COMP_5'SS_-1147_CE/RET_INTRON | chr9_31164673_31167560_- | chr9_31164673_31168707_- |
| Adgrg2 | COMP_5'SS_1988_CE | chrX_160463369_160465577_+ | chrX_160461381_160465577_+ |
| Rbm10 | COMP_5'SS_-1754_CE | chrX_20639502_20642743_+ | chrX_20641256_20642743_+ |
| Ankrd39 | COMP_5'SS_-176 | chr1_36542865_36546909_- | chr1_36542865_36547085_- |
| Abi1 | COMP_3'SS_727_CE | chr2_22963228_22971082_- | chr2_22962501_22971082_- |
| H2afy | COMP_5'SS_-1506_CE | chr13_56084285_56088251_- | chr13_56084285_56089757_- |
| Rap1gap | COMP_3'SS_-946_CE | chr4_137721957_137723745_+ | chr4_137721957_137724691_+ |
| Psmg4 | COMP_5'SS_2887_CE | chr13_34166136_34177980_+ | chr13_34163249_34177980_+ |
| Susd6 | COMP_5'SS_-23033_CE | chr12_80828243_80868426_+ | chr12_80851276_80868426_+ |
| Supt20 | COMP_3'SS_-701 | chr3_54715689_54716637_+ | chr3_54715689_54717338_+ |
| Mien1 | UNIDNT_COMP/RET_INTRON | chr11_98438744_98438857_- | NA |
| Ubr4 | COMP_5'SS_753_CE | chr4_139432514_139433619_+ | chr4_139431761_139433619_+ |
| Rps24 | COMP_3'SS_-345_CE | chr14_24493493_24495429_+ | chr14_24493493_24495774_+ |
| Sec16a | COMP_3'SS_-825_CE | chr2_26412913_26414370_- | chr2_26413738_26414370_- |
| Tcf19 | COMP_5'SS_48 | chr17_35516430_35516758_- | chr17_35516430_35516710_- |
| Pus1 | COMP_5'SS_-322 | chr5_110779897_110780239_- | chr5_110779897_110780561_- |
| Ewsr1 | DUAL_COMP | chr11_5078579_5082283_- | chr11_5079519_5082252_- |
| Il6st | COMP_5'SS_4755_CE | chr13_112470681_112472586_+ | chr13_112465926_112472586_+ |
| Tmem183a | COMP_3'SS_702_CE | chr1_134353137_134354660_- | chr1_134352435_134354660_- |
| Enah | nonME_COMP/RET_INTRON | chr1_181919629_181921657_- | chr1_181921768_181923559_- |
| Pex2 | COMP_3'SS_968_CE/RET_INTRON | chr3_5562732_5563164_- | chr3_5561764_5563164_- |
| Ppp1r12a | COMP_3'SS_-568_CE/RET_INTRON | chr10_108251944_108252756_+ | chr10_108251944_108253324_+ |
| Flnb | COMP_5'SS_-3389_CE | chr14_7919357_7923454_+ | chr14_7922746_7923454_+ |
| Ikbkg | COMP_5'SS_-5 | chrX_74424710_74427761_+ | chrX_74424715_74427761_+ |
| Adgrg6 | COMP_5'SS_1807_CE | chr10_14405438_14409672_- | chr10_14405438_14407865_- |
| Lama2 | COMP_3'SS_2945_CE | chr10_27021472_27023271_- | chr10_27018527_27023271_- |
| Aox1 | COMP_3'SS_-1144_CE | chr1_58070163_58072033_+ | chr1_58070163_58073177_+ |
| Picalm | COMP_5'SS_4765_CE/RET_INTRON | chr7_90182368_90189149_+ | chr7_90177603_90189149_+ |
| Acly | COMP_3'SS_4427_CE | chr11_100503189_100504202_- | chr11_100498762_100504202_- |
| Atp5c1 | COMP_3'SS_645_CE/RET_INTRON | chr2_10056806_10058995_- | chr2_10056161_10058995_- |
| 2610005L07Rik | UNIDNT_COMP/RET_INTRON | chr8_20399468_20401376_- | NA |
| Flywch1 | COMP_5'SS_1180_CE | chr17_23763318_23771414_- | chr17_23763318_23770234_- |
| Cuedc2 | COMP_3'SS_-5710_CE | chr19_46332701_46338568_- | chr19_46338411_46338568_- |
| Anln | COMP_5'SS_-1673_CE | chr9_22339088_22349246_- | chr9_22339088_22350919_- |
| Slc25a26 | COMP_3'SS_16078_CE | chr6_94535768_94592411_+ | chr6_94535768_94576333_+ |
| Shkbp1 | COMP_3'SS_1844_CE | chr7_27347172_27348331_- | chr7_27345328_27348331_- |
| Hist1h2bc | COMP_5'SS_1233_CE | chr13_23685854_23692175_+ | chr13_23684621_23692175_+ |
| Tcof1 | COMP_3'SS_4457_CE | chr18_60827447_60828364_- | chr18_60822990_60828364_- |
| Trnau1ap | COMP_5'SS_-4001_CE | chr4_132325260_132325351_- | chr4_132325260_132329352_- |
| Flnb | COMP_5'SS_3356_CE | chr14_7922713_7923454_+ | chr14_7919357_7923454_+ |
| Cemip | COMP_3'SS_79623_CE | chr7_84083044_84086257_- | chr7_84003421_84086257_- |
| Nfib | COMP_3'SS_-13580_CE | chr4_82296811_82327728_- | chr4_82310391_82327728_- |
| Nav2 | COMP_3'SS_-401 | chr7_49572453_49575246_+ | chr7_49572453_49575647_+ |
| Scrib | COMP_5'SS_-931_CE | chr15_76061223_76061738_- | chr15_76061223_76062669_- |
| Ccnt2 | COMP_5'SS_-310 | chr1_127797886_127799389_+ | chr1_127798196_127799389_+ |
| Ccdc136 | COMP_3'SS_-5917_CE | chr6_29399609_29399953_+ | chr6_29399609_29405870_+ |
| Map3k7 | COMP_5'SS_2438_CE | chr4_31994954_32002088_+ | chr4_31992516_32002088_+ |
| Acaca | COMP_5'SS_-9801_CE | chr11_84280510_84292890_+ | chr11_84290311_84292890_+ |
| Tbccd1 | COMP_5'SS_-740 | chr16_22842107_22856754_- | chr16_22842107_22857494_- |
| Anln | COMP_3'SS_10212_CE | chr9_22349300_22350919_- | chr9_22339088_22350919_- |
| B4galnt1 | UNIDNT_COMP/RET_INTRON | chr10_127167881_127169743_+ | NA |
| Abi1 | COMP_5'SS_-7869_CE | chr2_22962501_22963213_- | chr2_22962501_22971082_- |
| Sytl2 | COMP_5'SS_2829_CE/RET_INTRON | chr7_90395282_90396473_+ | chr7_90392453_90396473_+ |
| Fgfr1op | COMP_5'SS_2570_CE | chr17_8175578_8182227_+ | chr17_8173008_8182227_+ |
| Lats1 | UNIDNT_COMP/RET_INTRON | chr10_7703120_7705459_+ | NA |
| Tial1 | COMP_5'SS_-1390_CE | chr7_128446270_128446730_- | chr7_128446270_128448120_- |
| Kcnj15 | COMP_5'SS_-12435_CE | chr16_95279461_95295590_+ | chr16_95291896_95295590_+ |
| Ythdf3 | COMP_5'SS_13656_CE | chr3_16203219_16203825_+ | chr3_16189563_16203825_+ |
| Macf1 | COMP_3'SS_3047_CE | chr4_123364074_123365272_- | chr4_123361027_123365272_- |
| Adar | COMP_5'SS_-78 | chr3_89745523_89745819_+ | chr3_89745601_89745819_+ |
| Iffo2 | COMP_5'SS_-26214_CE | chr4_139575597_139603212_+ | chr4_139601811_139603212_+ |
| Map3k7 | COMP_3'SS_-7215_CE | chr4_31992516_31994873_+ | chr4_31992516_32002088_+ |
| Fbxw17 | COMP_5'SS_151 | chr13_50420028_50423201_+ | chr13_50419877_50423201_+ |
| Myl6 | COMP_3'SS_731_CE | chr10_128491764_128492058_- | chr10_128491033_128492058_- |
| Pcmt1 | COMP_5'SS_-47/RET_INTRON | chr10_7630976_7638008_- | chr10_7630976_7638055_- |
| Ctage5 | COMP_5'SS_1592_CE | chr12_59146362_59146846_+ | chr12_59144770_59146846_+ |
| Rhot1 | COMP_3'SS_-8228_CE/RET_INTRON | chr11_80254862_80257513_+ | chr11_80254862_80265741_+ |
| Uggt1 | COMP_3'SS_472_CE | chr1_36176799_36177517_- | chr1_36176327_36177517_- |
| Pex2 | COMP_5'SS_-496_CE/RET_INTRON | chr3_5561764_5562668_- | chr3_5561764_5563164_- |
| 1110038B12Rik | COMP_3'SS_372 | chr17_34950847_34951884_- | chr17_34950475_34951884_- |
| Cdk12 | COMP_5'SS_4431_CE/RET_INTRON | chr11_98250100_98263469_+ | chr11_98245669_98263469_+ |
| Fbxl12 | COMP_5'SS_-593 | chr9_20639198_20641430_- | chr9_20639198_20642023_- |
| Chd9 | COMP_3'SS_-41272_CE | chr8_90876708_90890976_+ | chr8_90876708_90932248_+ |
| Cables2 | COMP_5'SS_-961/RET_INTRON | chr2_180261681_180262116_- | chr2_180261681_180263077_- |
| Actr5 | COMP_3'SS_19 | chr2_158635426_158636026_+ | chr2_158635426_158636007_+ |
| Meaf6 | COMP_3'SS_4222_CE | chr4_125103031_125107651_+ | chr4_125103031_125103429_+ |
| Dst | COMP_3'SS_-2979_CE | chr1_34295536_34296741_+ | chr1_34295536_34299720_+ |
| Adk | COMP_5'SS_-23621_CE | chr14_21052700_21092366_+ | chr14_21076321_21092366_+ |
| Ssh3 | COMP_3'SS_4 | chr19_4267978_4268544_- | chr19_4267974_4268544_- |
| Wwp2 | COMP_5'SS_4548_CE | chr8_107545346_107545443_+ | chr8_107540798_107545443_+ |
| Dhdds | COMP_5'SS_142 | chr4_134000393_134000866_- | chr4_134000393_134000724_- |
| Ntan1 | COMP_3'SS_-454 | chr16_13832434_13834680_+ | chr16_13832434_13835134_+ |
| Itpr1 | COMP_3'SS_3712_CE | chr6_108363705_108369010_+ | chr6_108363705_108365298_+ |
| Macf1 | COMP_5'SS_-1216_CE | chr4_123361027_123364056_- | chr4_123361027_123365272_- |
| Tcf25,Gm20388 | COMP_3'SS_-2071_CE | chr8_123400697_123401075_+ | chr8_123400697_123403146_+ |
| Ankrd10 | COMP_5'SS_-4742_CE | chr8_11619297_11623696_- | chr8_11619297_11628438_- |
| E2f1 | COMP_5'SS_-422 | chr2_154564508_154569066_- | chr2_154564508_154569488_- |
| Evi5 | COMP_3'SS_-11062_CE | chr5_107799260_107812357_- | chr5_107810322_107812357_- |
| Cask | COMP_3'SS_-1459_CE | chrX_13550982_13554877_- | chrX_13552441_13554877_- |
| Fam214a | COMP_3'SS_-50356_CE | chr9_74953830_74953908_+ | chr9_74953830_75004264_+ |
| Phf19 | COMP_5'SS_-659/RET_INTRON | chr2_34898159_34898923_- | chr2_34898159_34899582_- |
| Mdm1 | COMP_3'SS_-361 | chr10_118152274_118156833_+ | chr10_118152274_118157194_+ |
| Cxadr | COMP_5'SS_3844_CE | chr16_78333002_78333361_+ | chr16_78329158_78333361_+ |
| Etl4 | COMP_5'SS_-8590_CE | chr2_20798783_20807901_+ | chr2_20807373_20807901_+ |
| Camk2d | COMP_5'SS_-1208_CE | chr3_126840365_126842757_+ | chr3_126841573_126842757_+ |
| H2afy | COMP_3'SS_1515_CE | chr13_56089857_56096123_- | chr13_56088342_56096123_- |
| Uspl1 | COMP_3'SS_637/RET_INTRON | chr5_149184806_149188344_+ | chr5_149184806_149187707_+ |
| Enpp2 | COMP_3'SS_-2171_CE | chr15_54875760_54882129_- | chr15_54877931_54882129_- |
| Zfp950 | COMP_3'SS_2102_CE | chr19_61120452_61127006_- | chr19_61118350_61127006_- |
| Arhgef11 | COMP_5'SS_-1048_CE | chr3_87733504_87735344_+ | chr3_87734552_87735344_+ |
| Col5a1 | COMP_3'SS_-130 | chr2_28025705_28028966_+ | chr2_28025705_28029096_+ |
| Mff | COMP_3'SS_3437_CE | chr1_82735507_82750519_+ | chr1_82735507_82747082_+ |
| Myl6 | COMP_5'SS_-339_CE | chr10_128491033_128491719_- | chr10_128491033_128492058_- |
| Prrx1 | COMP_5'SS_3784_CE | chr1_163248396_163257760_- | chr1_163248396_163253976_- |
| Abcd4 | COMP_5'SS_2698_CE | chr12_84605384_84608569_- | chr12_84605384_84605871_- |
| Srsf11 | COMP_3'SS_-409 | chr3_158022950_158026724_- | chr3_158023359_158026724_- |
| Dnal4 | COMP_3'SS_832_CE | chr15_79765853_79774358_- | chr15_79765021_79774358_- |
| Taf1d | COMP_3'SS_-855_CE | chr9_15306283_15306872_+ | chr9_15306283_15307727_+ |
| Huwe1 | COMP_3'SS_-712_CE/RET_INTRON | chrX_151857913_151859454_+ | chrX_151857913_151860166_+ |
| 9430038I01Rik | COMP_5'SS_10416_CE | chr7_137376351_137387370_- | chr7_137376351_137376954_- |
| Arhgap12 | COMP_3'SS_-4070_CE | chr18_6052923_6057516_- | chr18_6056993_6057516_- |
| Angel2 | COMP_3'SS_4577_CE | chr1_190928758_190937443_+ | chr1_190928758_190932866_+ |
| Picalm | COMP_3'SS_-6931_CE/RET_INTRON | chr7_90177603_90182218_+ | chr7_90177603_90189149_+ |
| Cask | COMP_5'SS_-1426_CE | chrX_13549361_13550946_- | chrX_13549361_13552372_- |
| Rcor3 | COMP_5'SS_-14643_CE | chr1_192101155_192101625_- | chr1_192101155_192116268_- |
| Lpcat2 | COMP_5'SS_18143_CE | chr8_92908969_92909186_+ | chr8_92890826_92909186_+ |
| Col12a1 | COMP_3'SS_1172_CE | chr9_79601179_79602199_- | chr9_79600007_79602199_- |
| Nexn | COMP_3'SS_-674_CE | chr3_152253129_152255284_- | chr3_152253803_152255284_- |
| Zfp598 | COMP_5'SS_9 | chr17_24678713_24679036_+ | chr17_24678704_24679036_+ |
| Tsku | COMP_3'SS_-5851_CE | chr7_98353616_98361241_- | chr7_98359467_98361241_- |
| Dnm1l | COMP_5'SS_-1007_CE | chr16_16316778_16318444_- | chr16_16316778_16319451_- |
| Tpm1 | COMP_5'SS_-4546_CE/RET_INTRON | chr9_67036128_67043577_- | chr9_67036128_67048123_- |
| Adgra2 | COMP_3'SS_319 | chr8_27119851_27120765_+ | chr8_27119851_27120446_+ |
| Eps8 | COMP_5'SS_-2318_CE | chr6_137519516_137519834_- | chr6_137519516_137522152_- |
| Rian | COMP_3'SS_-3563_CE | chr12_109627153_109634275_+ | chr12_109627153_109637838_+ |
| Rpusd1 | COMP_5'SS_544 | chr17_25728391_25728522_+ | chr17_25727847_25728522_+ |
| Mdm1 | DUAL_COMP | chr10_118148115_118150821_+ | chr10_118146971_118150788_+ |
| Pphln1 | COMP_3'SS_-3730_CE | chr15_93410783_93420283_+ | chr15_93410783_93424013_+ |
| Bdp1 | COMP_3'SS_-57 | chr13_100030932_100035083_- | chr13_100030989_100035083_- |
| Tmem183a | COMP_5'SS_-1686_CE | chr1_134352435_134352974_- | chr1_134352435_134354660_- |
| Ip6k2 | DUAL_COMP | chr9_108796783_108797206_+ | chr9_108796396_108797283_+ |
| Kif21a | COMP_3'SS_5247_CE | chr15_90964995_90965466_- | chr15_90959748_90965466_- |
| Kif21a | COMP_5'SS_-492_CE | chr15_90959748_90964974_- | chr15_90959748_90965466_- |
| Ubap2l | UNIDNT_COMP/RET_INTRON | chr3_90006617_90008255_- | NA |
| Spag9 | COMP_5'SS_1155_CE | chr11_94113301_94114172_+ | chr11_94112146_94114172_+ |
| Suco | COMP_5'SS_-1645_CE | chr1_161845358_161845559_- | chr1_161845358_161847204_- |
| Usp48 | COMP_3'SS_-831 | chr4_137635008_137636933_+ | chr4_137635008_137637764_+ |
| Kif21a | COMP_5'SS_1971_CE | chr15_90952818_90956294_- | chr15_90952818_90954323_- |
| Akap12 | COMP_5'SS_20852_CE | chr10_4334709_4353141_+ | chr10_4313857_4353141_+ |
| Erap1 | UNIDNT_COMP/RET_INTRON | chr13_74662429_74663408_+ | NA |
| Pam | COMP_3'SS_-11288_CE | chr1_97853232_97884125_- | chr1_97864520_97884125_- |
| Nek4 | COMP_5'SS_3932_CE | chr14_30974496_30975222_+ | chr14_30970564_30975222_+ |
| Abi3bp | COMP_5'SS_-2636_CE | chr16_56611084_56642488_+ | chr16_56613720_56642488_+ |
| Gatad2a | COMP_5'SS_-759_CE/RET_INTRON | chr8_69911869_69912163_- | chr8_69911869_69912922_- |
| Meaf6 | COMP_3'SS_671_CE | chr4_125103031_125108322_+ | chr4_125103031_125107651_+ |
| Tpm2 | COMP_3'SS_359/RET_INTRON | chr4_43519078_43519226_- | chr4_43518719_43519226_- |
| BC031181 | COMP_5'SS_-114 | chr18_75005900_75008650_+ | chr18_75006014_75008650_+ |
| Trio | COMP_5'SS_-17555_CE | chr15_27774173_27788074_- | chr15_27774173_27805629_- |
| Dnal4 | COMP_5'SS_-8572_CE/RET_INTRON | chr15_79765021_79765786_- | chr15_79765021_79774358_- |
| Rsrp1 | COMP_3'SS_-958_CE | chr4_134925432_134925776_+ | chr4_134925432_134926734_+ |
| Serpinh1 | COMP_5'SS_-1614_CE | chr7_99349464_99350209_- | chr7_99349464_99351823_- |
| Ncor1 | COMP_3'SS_-919 | chr11_62359015_62366963_- | chr11_62359934_62366963_- |
| Fn1 | COMP_5'SS_-1310_CE/RET_INTRON | chr1_71602497_71603659_- | chr1_71602497_71604969_- |
| Snx13 | COMP_3'SS_-33 | chr12_35112504_35119732_+ | chr12_35112504_35119765_+ |
| Sidt2 | COMP_3'SS_-642_CE | chr9_45947055_45947835_- | chr9_45947697_45947835_- |
| Suco | COMP_3'SS_222_CE | chr1_161845580_161847204_- | chr1_161845358_161847204_- |
| Nucb2 | COMP_5'SS_-5141_CE | chr7_116504484_116521836_+ | chr7_116509625_116521836_+ |
| Prrc2c | COMP_5'SS_-2592_CE | chr1_162673656_162674098_- | chr1_162673656_162676690_- |
| Dzip1 | COMP_5'SS_528 | chr14_118923167_118924424_- | chr14_118923167_118923896_- |
| Kdm6b | COMP_5'SS_-4241_CE | chr11_69408781_69409291_- | chr11_69408781_69413532_- |
| Taf1d | COMP_5'SS_707_CE | chr9_15306990_15307727_+ | chr9_15306283_15307727_+ |
| Miip | COMP_3'SS_187_CE/RET_INTRON | chr4_147863136_147865188_- | chr4_147862949_147865188_- |
| Gigyf1 | COMP_3'SS_-190 | chr5_137518612_137518687_+ | chr5_137518612_137518877_+ |
| RP23-414I24.8, Zfp865, Ccdc106 | COMP_3'SS_3772_CE | chr7_5020655_5032762_+ | chr7_5020655_5028990_+ |
| Zbtb38 | COMP_3'SS_59575_CE | chr9_96748604_96751301_- | chr9_96689029_96751301_- |
| Hexa | nonME_COMP/RET_INTRON | chr9_59562026_59562295_+ | chr9_59561077_59561942_+ |
| Pkd1 | COMP_5'SS_704_CE | chr17_24587292_24587399_+ | chr17_24586588_24587399_+ |
| Msh3 | COMP_3'SS_-91 | chr13_92351360_92353224_- | chr13_92351451_92353224_- |
| Card19 | COMP_5'SS_1263_CE | chr13_49203789_49205191_- | chr13_49203789_49203928_- |
| Adgrb2 | COMP_5'SS_440 | chr4_130019720_130021218_+ | chr4_130019280_130021218_+ |
| Col5a1 | COMP_5'SS_-130 | chr2_28029035_28032590_+ | chr2_28029165_28032590_+ |
| Arl13b | COMP_3'SS_-6082_CE | chr16_62795708_62802719_- | chr16_62801790_62802719_- |
| Fn1 | COMP_3'SS_1432_CE/RET_INTRON | chr1_71603929_71604969_- | chr1_71602497_71604969_- |
| Endov | COMP_5'SS_-224 | chr11_119499661_119500590_+ | chr11_119499885_119500590_+ |
| Lrrc16a | COMP_5'SS_2932_CE | chr13_24013460_24022495_- | chr13_24013460_24019563_- |
| Nprl3 | COMP_3'SS_-3687_CE | chr11_32255568_32263024_- | chr11_32259255_32263024_- |
| Prrc2a | COMP_3'SS_-26 | chr17_35150682_35150816_- | chr17_35150708_35150816_- |
| Slc9a3r2 | COMP_3'SS_268/RET_INTRON | chr17_24640657_24641708_- | chr17_24640389_24641708_- |
| Rasa4 | COMP_5'SS_-368 | chr5_136101689_136102234_+ | chr5_136102057_136102234_+ |
| Gsn | COMP_5'SS_-15818_CE | chr2_35266903_35283904_+ | chr2_35282721_35283904_+ |
| Mtmr2 | COMP_5'SS_1328_CE | chr9_13783710_13785851_+ | chr9_13782382_13785851_+ |
| Abcd4 | DUAL_COMP | chr12_84605963_84606099_- | chr12_84605384_84608569_- |
| Smek2 | nonME_COMP/RET_INTRON | chr11_29188574_29194572_+ | chr11_29194650_29196173_+ |
| Atp6v0a1 | COMP_3'SS_-21 | chr11_101021182_101026659_+ | chr11_101021182_101026680_+ |
| Ltbp3 | COMP_5'SS_4 | chr19_5740985_5741334_+ | chr19_5740981_5741334_+ |
| Ubr5 | nonME_COMP/RET_INTRON | chr15_37971892_37972902_- | chr15_37970347_37971747_- |
| Spag9 | COMP_3'SS_-910_CE | chr11_94112146_94113262_+ | chr11_94112146_94114172_+ |
| Pcgf3 | COMP_5'SS_156 | chr5_108461607_108472071_+ | chr5_108461451_108472071_+ |
| Kirrel3 | COMP_3'SS_3221_CE | chr9_35023652_35028331_+ | chr9_35023652_35025110_+ |
| Isca2 | COMP_5'SS_231/RET_INTRON | chr12_84773908_84774542_+ | chr12_84773677_84774542_+ |
| Mark2 | COMP_3'SS_704 | chr19_7281959_7282725_- | chr19_7281255_7282725_- |
| Neurl4 | COMP_5'SS_-267 | chr11_69903452_69903864_+ | chr11_69903719_69903864_+ |
| Rbm25 | DUAL_COMP | chr12_83639191_83642628_+ | chr12_83632417_83642401_+ |
| Slc38a7 | COMP_3'SS_-4139_CE | chr8_95848768_95853485_- | chr8_95852907_95853485_- |
| Btaf1 | COMP_3'SS_-637_CE | chr19_36981821_36982811_+ | chr19_36981821_36983448_+ |
| Ppip5k2 | COMP_3'SS_-1373_CE/RET_INTRON | chr1_97719906_97723669_- | chr1_97721279_97723669_- |
| Zfp655 | COMP_5'SS_-1789_CE | chr5_145233602_145235736_+ | chr5_145235391_145235736_+ |
| P3h3 | COMP_3'SS_-57 | chr6_124854390_124855080_- | chr6_124854447_124855080_- |
| Uggt1 | COMP_5'SS_742_CE | chr1_36176327_36177517_- | chr1_36176327_36176775_- |
| Pitpnb | COMP_3'SS_-983_CE/RET_INTRON | chr5_111383110_111385519_+ | chr5_111383110_111386502_+ |
| Vcan | COMP_3'SS_-12296_CE | chr13_89693501_89712204_- | chr13_89705797_89712204_- |
| Tm9sf2 | DUAL_COMP | chr14_122118542_122122206_+ | chr14_122118599_122122215_+ |
| Adgrg6 | COMP_5'SS_-1942_CE | chr10_14450664_14456137_- | chr10_14450664_14458079_- |
| Prrc2c | COMP_3'SS_-2185_CE | chr1_162673656_162676690_- | chr1_162675841_162676690_- |
| Iqce | COMP_5'SS_-621/RET_INTRON | chr5_140691670_140692793_- | chr5_140691670_140693414_- |
| Slc22a17 | COMP_5'SS_193 | chr14_54909825_54912241_- | chr14_54909825_54912048_- |
| Sdccag3 | COMP_5'SS_-94 | chr2_26387671_26388491_- | chr2_26387671_26388585_- |
| Pkd1 | COMP_3'SS_221_CE | chr17_24586588_24587399_+ | chr17_24586588_24587178_+ |
| Map2 | COMP_5'SS_635_CE | chr1_66417185_66420134_+ | chr1_66416550_66420134_+ |
| Mief1 | COMP_3'SS_-1123_CE | chr15_80234181_80234681_+ | chr15_80234181_80235804_+ |
| Atp13a1 | COMP_3'SS_-68 | chr8_69802142_69803459_+ | chr8_69802142_69803527_+ |
| Smpd4 | COMP_3'SS_-3014_CE/RET_INTRON | chr16_17632057_17635481_+ | chr16_17632057_17638495_+ |
| Psmc2 | UNIDNT_COMP/RET_INTRON | chr5_21803272_21803365_+ | NA |
| Brd1 | COMP_3'SS_-393_CE | chr15_88700877_88707033_- | chr15_88701270_88707033_- |
| Fnbp4 | COMP_3'SS_-1155_CE | chr2_90768700_90774749_+ | chr2_90768700_90775904_+ |
| Mief1 | COMP_5'SS_-599_CE | chr15_80234181_80235804_+ | chr15_80234780_80235804_+ |
| Usp48 | DUAL_COMP | chr4_137636987_137637759_+ | chr4_137635008_137637764_+ |
| Rbm25 | COMP_5'SS_10102_CE | chr12_83642519_83642628_+ | chr12_83632417_83642628_+ |
| Ptprk | COMP_5'SS_218_CE | chr10_28570191_28573367_+ | chr10_28569973_28573367_+ |
